# The Bladder Microbiome, Metabolome, Cytokines, and Phenotypes in Patients with Systemic Lupus Erythematosus

**DOI:** 10.1128/spectrum.00212-22

**Published:** 2022-08-01

**Authors:** Fengping Liu, Jingjie Du, Qixiao Zhai, Jialin Hu, Aaron W. Miller, Tianli Ren, Yangkun Feng, Peng Jiang, Lei Hu, Jiayi Sheng, Chaoqun Gu, Ren Yan, Longxian Lv, Alan J. Wolfe, Ninghan Feng

**Affiliations:** a Wuxi School of Medicine, Jiangnan University, Wuxi, Jiangsu, China; b Department of Microbiology and Immunology, Stritch School of Medicine, Loyola University Chicago, Maywood, Illinois, USA; c State Key Laboratory of Food Science and Technology and School of Food Science and Technology, Jiangnan University, Wuxi, Jiangsu, China; d Department of Urology, Affiliated Wuxi No. 2 Hospital, Nanjing Medical University, Wuxi, Jiangsu, China; e Glickman Urological and Kidney Institute, Cleveland, Ohio, USA; f Department of Rheumatology, Affiliated Wuxi No. 2 Hospital, Nanjing Medical University, Wuxi, China; g School of Medicine, Nantong University, Nantong, Jiangsu, China; h Collaborative Innovation Center for Diagnosis and Treatment of Infectious Diseases, State Key Laboratory for Diagnosis and Treatment of Infectious Diseases, The First Affiliated Hospital, School of Medicine, Zhejiang University, Hangzhou, Zhejiang, China; Wayne State University

**Keywords:** bladder microbiome, complement, disease profile, systemic lupus erythematosus, urinary cytokines, urinary metabolome

## Abstract

Emerging studies reveal unique bacterial communities in the human bladder, with alteration of composition associated to disease states. Systemic lupus erythematosus (SLE) is a complex autoimmune disease that is characterized by frequent impairment of the kidney. Here, we explored the bladder microbiome, metabolome, and cytokine profiles in SLE patients, as well as correlations between microbiome and metabolome, cytokines, and disease profiles. We recruited a group of 50 SLE patients and 50 individually matched asymptomatic controls. We used transurethral catheterization to collect urine samples, 16S rRNA gene sequencing to profile bladder microbiomes, and liquid chromatography-tandem mass spectrometry to perform untargeted metabolomic profiling. Compared to controls, SLE patients possessed unique bladder microbial communities and increased alpha diversity. These differences were accompanied by differences in urinary metabolomes, cytokines, and patients’ disease profiles. The SLE-enriched genera, including *Bacteroides*, were positively correlated with several SLE-enriched metabolites, including olopatadine. The SLE-depleted genera, such as Pseudomonas, were negatively correlated to SLE-depleted cytokines, including interleukin-8. Alteration of the bladder microbiome was associated with disease profile. For example, the genera *Megamonas* and *Phocaeicola* were negatively correlated with serum complement component 3, and Streptococcus was positively correlated with IgG. Our present study reveals associations between the bladder microbiome and the urinary metabolome, cytokines, and disease phenotypes. Our results could help identify biomarkers for SLE.

**IMPORTANCE** Contrary to dogma, the human urinary bladder possesses its own unique bacterial community with alteration of composition associated with disease states. Systemic lupus erythematosus (SLE) is a complex autoimmune disease often characterized by kidney impairment. Here, we explored the bladder microbiome, metabolome, and cytokine profiles in SLE patients, as well as correlations between the microbiome and metabolome, cytokines, and disease profiles. Compared to controls, SLE patients possessed a unique bladder microbial community and elevated alpha diversity. These differences were accompanied by differences in bladder metabolomes, cytokines, and patients’ disease profiles. SLE-enriched genera were positively correlated with several SLE-enriched metabolites. SLE-depleted genera were negatively correlated to SLE-depleted cytokines. Alteration of the bladder microbiome was associated with disease profile. Thus, our study reveals associations between the bladder microbiome and the bladder metabolome, cytokines, and disease phenotypes. These results could help identify biomarkers for SLE.

## INTRODUCTION

Systemic lupus erythematosus (SLE) is a complex autoimmune disease with a chronic relapsing-remitting course that can damage multiple organs and range from mild to life-threatening illness (1). The kidney is one of the most commonly impaired organs, and lupus nephritis (LN) has been reported in approximately 50% of SLE patients (2); 10 to 30% of LN patients progress to kidney failure that requires kidney replacement therapy (2), and the mortality rate within 5 years of onset directly attributed to kidney disease is 5% to 25% of patients with proliferative LN (2). Currently, no cure for SLE has been found and the pathogenesis of SLE is currently poorly understood.

Alteration of microbial compositions in the gut, oral mucosa, or tegument has been reported to be associated with SLE disease manifestations (3–15). Specifically, SLE patients exhibit compositional alterations to the gut microbiome, characterized by lower bacterial diversity (3, 4, 6–8, 14), decreased *Firmicutes*/*Bacteroidetes* (F/B) ratio (5, 7, 14, 15), and increased abundance of *Lactobacillaceae* (6, 8, 11). Similarly, SLE patients exhibit reduced microbial diversity and altered microbial communities in their gums and skin (10, 12). However, the relationship of SLE on microbial communities and metabolic output in the bladder and other urogenital niches has not been studied.

Once considered sterile, the bladder is now known to possess microbial communities (bladder microbiome) in individuals with and without urinary tract infections (16). Furthermore, disruption (dysbiosis) of the bladder microbiome is associated with urinary tract disorders, especially urgency urinary incontinence and urinary tract infection (17). However, most studies of bladder microbiome involve only US participants; only a few reports involve Chinese participants (18, 19).

Immune response and metabolic output can bridge the gap between the microbiome and SLE phenotypes. Urine is often used to assess metabolic status of the body (20). For example, Yan and coworkers (21) found 23 metabolites dramatically increased in SLE patients’ feces compared to healthy controls, including valine, cysteine, and uracil. Also, as renal impairment is one of the most serious manifestations of SLE and urine cytokines derived directly from the diseased kidney accumulate in the urine, the level of inflammatory factors in urine may be used as an indicator of chronic inflammation and disease progression. Brugos and coworkers (22) found that interleukin-1 (IL-1) and tumor necrosis factor-α (TNF-α) were elevated significantly in the urine of patients without renal disease, while interferon-γ (IFN-γ) was elevated in the urine of LN patients. However, the relationship between immunity, metabolism, and microbiome in the bladder of SLE patients is unclear.

Given that SLE often affects the kidney, and LN is a common manifestation that leads to irreversible renal impairment, we hypothesize that the bladder microbiomes of individuals with and without SLE differ, and the differences correlate with specific urinary metabolites and cytokines, along with patients’ clinical profiles. To test this hypothesis, we analyzed urine obtained by transurethral catheterization from participants with and without SLE. We also compared the gut and vaginal microbiomes of a subset of SLE patients to their bladder microbiome to determine whether these adjacent microbiomes might influence the composition of the bladder microbiome.

## RESULTS

### Demographics.

In the cross-sectional study, we assessed the bladder microbiome, metabolite profile, and cytokine profile of a total of 50 SLE patients and 50 sex-, age-, body mass index (BMI)-, and comorbid disease-matched asymptomatic controls (Controls) (Table 1). Both groups were 88% (*n* = 44) female and 12% (*n* = 6) male. As expected, the SLE groups had lower serum concentrations of complement component 3 (C3) and component 4 (C4) but higher serum concentrations of IgG and uric acid; they also had more urinary white blood cells, red blood cells, and leukocyte esterase (*P < *0.05 for all comparisons). Of the 50 SLE patients, 38 (76%) had LN (Table S6). The SLE group also had significantly elevated intake of calcium and zinc (Table S7); these were listed as confounding factors in the downstream analyses.

**TABLE 1:** Characteristics of controls and SLE patients^*a*^

Parameters	Value for cohort (*n*^*b*^)^*c*^ or statistic		*P* value^*d*^
	Controls (*n* = 50)	SLE (*n* = 50)	
Female sex, *n* (%)	44 (88.00)	44 (88.00)	1.000
Age (yr)	49.22 ± 15.19	45.00 ± 14.61	0.160
Body-mass index (kg/m^2^)	23.98 ± 3.23	23.99 ± 2.65	0.992
Duration of SLE (yr)	NA	7.58 ± 5.97	NA
History of smoking, n (%)	0 (0.00)	0 (0.00)	1.000
History of drinking, *n* (%)	1 (2.00)	0 (0.00)	1.000
Comorbidity
Diabetes, *n* (%)	5 (10.00)	5 (10.00)	1.000
Hypertension, *n* (%)	10 (20.00)	10 (20.00)	1.000
Immunological features
Complement 3 (g/L)	1.20 ± 0.64	0.76 ± 0.23	< 0.001
Complement 4 (g/L)	0.30 ± 0.28	0.16 ± 0.06	< 0.001
IgA (g/L)	2.59 ± 0.85	2.60 ± 1.08	0.930
IgG (g/L)	10.98 ± 1.88	15.97 ± 7.86	< 0.001
IgM (g/L)	1.17 ± 0.51	1.01 ± 0.51	0.105
ESR (mm/h)^*e*^	NA	30.02 ± 22.56	NA
Renal function
Serum creatinine (μmol/L)	55.23 ± 10.75	65.79 ± 40.62	0.087
Blood urea nitrogen (mmol/L)	5.73 ± 5.99	5.46 ± 2.11	0.766
Serum uric acid (umol/L)	268.04 ± 72.56	318.86 ± 103.66	0.006
Estimated glomerular filtration rate (mL/min/1.73 m^2^)	114.40 ± 20.08	108.84 ± 38.26	0.374
Urinary creatinine (mg/dL)	1.37 ± 0.19	1.42 ± 0.25	0.268
Urine analysis
White blood cells (/μL)	2.22 ± 6.38	9.32 ± 22.40	< 0.001
Red blood cells (/μL)	0.61 ± 3.03	28.00 ± 131.45	< 0.001
Nitrites positive, *n* (%)	1 (2.00)	2 (4.00)	1.000
Leucocyte esterase, *n* (%)	0 (0)	12 (24.00)	< 0.001
LN
LN, *n* (%)	NA	38 (76.00)	NA
Duration of LN (yr)	NA	5.30 ± 3.60	NA
SLEDAI
Score	NA	16.16 ± 24.31	NA
Low SLEDAI [<8; *n* (%)]		8 (16.00)	NA
High SLEDAI [≥8; *n* (%)]		42 (84.00)	NA

a ESR, erythrocyte sedimentation rate; Ig, immunoglobulin; LN, lupus nephritis; NA, not applicable; SLEDAI, systemic lupus erythematosus disease activity index.

b *n*, number of subjects.

c Mean ± SD or *n* (%).

d Pearson Chi-square or Fisher’s exact test was used with categorical variables; Student's *t* test on normalized continuous variables and Wilcoxon rank-sum test were used on unnormalized continuous variables.

e ESR detection threshold was 15 mm/h.

### Bladder microbiome is altered in SLE patients.

**FIG 1:**
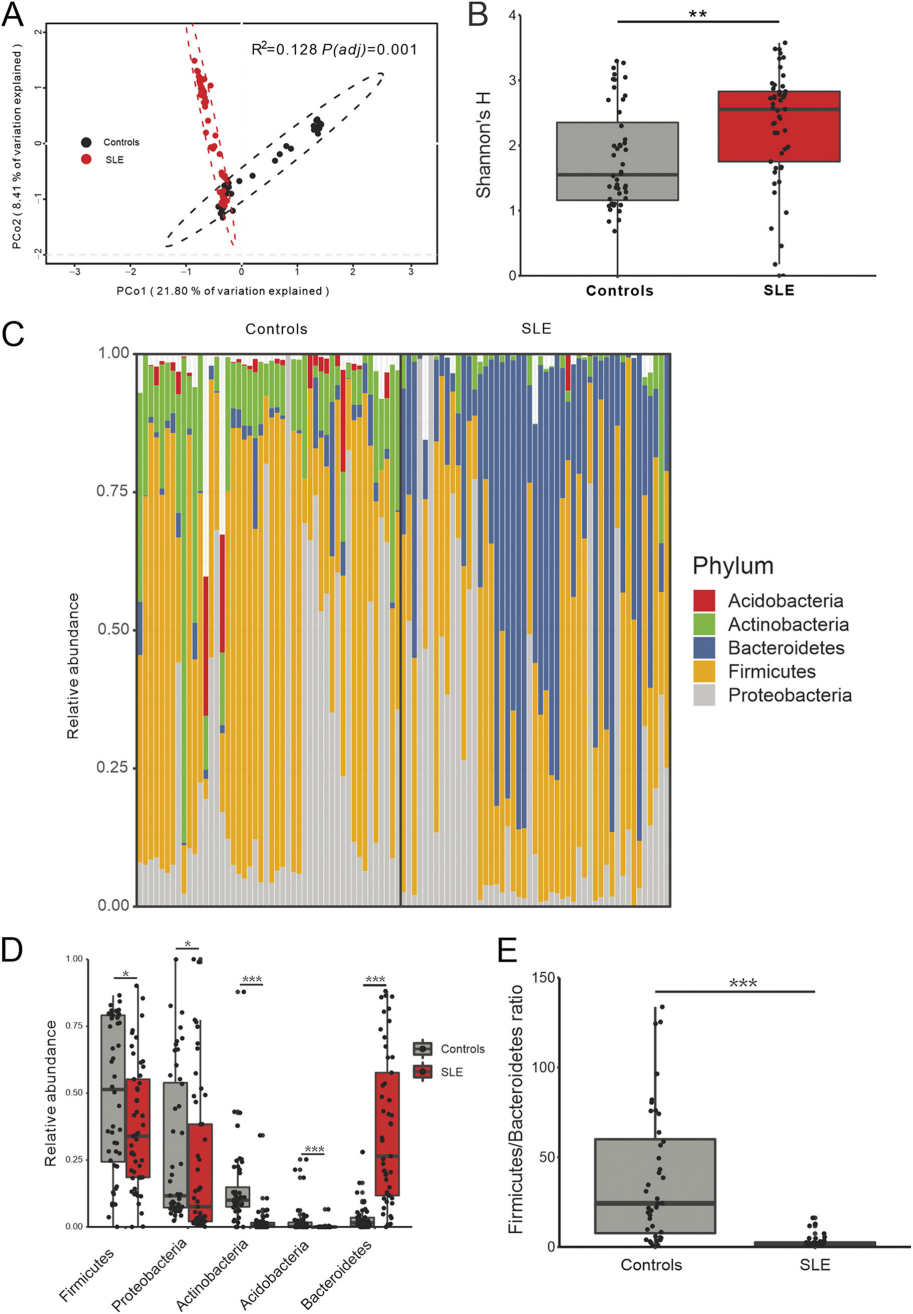
Bacterial composition, diversity and phylum difference between controls and SLE group. (A) PCoA based on Bray-Curtis distances at species level showed different microbial compositions between groups of SLE patients and controls. The 95% confidence ellipse is drawn for each group. Permutational multivariate analysis of variance (PERMANOVA) was performed for statistical comparisons of samples in the two groups. *P* value was adjusted by the Benjamini and Hochberg false discovery rate (FDR). (B) Bacterial diversity measured by Shannon index was calculated at the bacterial species level. Wilcoxon rank-sum test was performed and adjusted by Benjamini and Hochberg false discovery rate (FDR). **, *P*_adj_ < 0.01. (C) Microbial profile at the phylum level. Only phyla with more than 1% average relative abundances in all samples are shown. (D) Bacterial phyla that were differentially abundant between controls and SLE patients. *P* value was calculated using Wilcoxon rank-sum test and adjusted by Benjamini and Hochberg FDR. *, *P*_adj_ < 0.05 and ***, *P*_adj_ < 0.001. (E) *Firmicutes/Bacteroidetes* ratio differed in controls and SLE patients. *P* value was calculated using Wilcoxon rank-sum test and adjusted by Benjamini and Hochberg FDR. ***, *P*_adj_ < 0.001.

Of the 100 urine samples, we detected bacteria in 96% (48/50) controls and 98% (49/50) SLE samples. To test whether the bladder microbiome differs between SLE patients and asymptomatic controls, we first assessed the microbial community structure using all microbial species present within the bladder microbiome of each sample. Principal Coordinate Analysis (PCoA) of Bray-Curtis dissimilarities revealed differential clustering between the control and SLE groups (Fig. 1A; *R*^2^ = 0.128, adjusted *P* value [*P*_adj_] = 0.001), reflecting a dysbiotic urobiome in SLE patients. To determine whether the bacterial communities were affected by medication usage, we separated the SLE patients into subgroups based on their dosages of hydroxychloroquine and prednisone and performed PCoA; we found no differences between/among the subgroups (Fig. S2A and S2B; *R*^2^ = 0.013, *P*_adj_ = 0.886; *R*^2^ = 0.020, *P*_adj_ = 0.428). The disruption of microbial composition between the controls and SLE group was highlighted by the observation that species diversity (as measured by the Shannon’s H Index) was significantly elevated in the SLE group (Fig. 1B; *P*_adj_ < 0.05), likely due to increased evenness (Fig. S3A; *P*_adj_ < 0.05), as there was no significant difference in species richness (Fig. S3B).

Since we observed a clear difference in diversity, we assessed taxonomic signatures at the phylum level. The relative abundances of the five most abundant phyla (>1% average relative abundance of all urine samples) are displayed in Fig. 1C. The phyla *Firmicutes*, *Proteobacteria*, *Acidobacteria*, and *Actinobacteria* were significantly more abundant in controls, whereas *Bacteroidetes* was significantly more abundant in the SLE group (Fig. 1D; *P*_adj_ < 0.05). The *Firmicutes/Bacteroidetes* ratio was reduced significantly in SLE patients (Fig. 1E; *P*_adj_ < 0.05), consistent with previous studies of the gut microbiome of SLE patients (5, 23).

**FIG 2:**
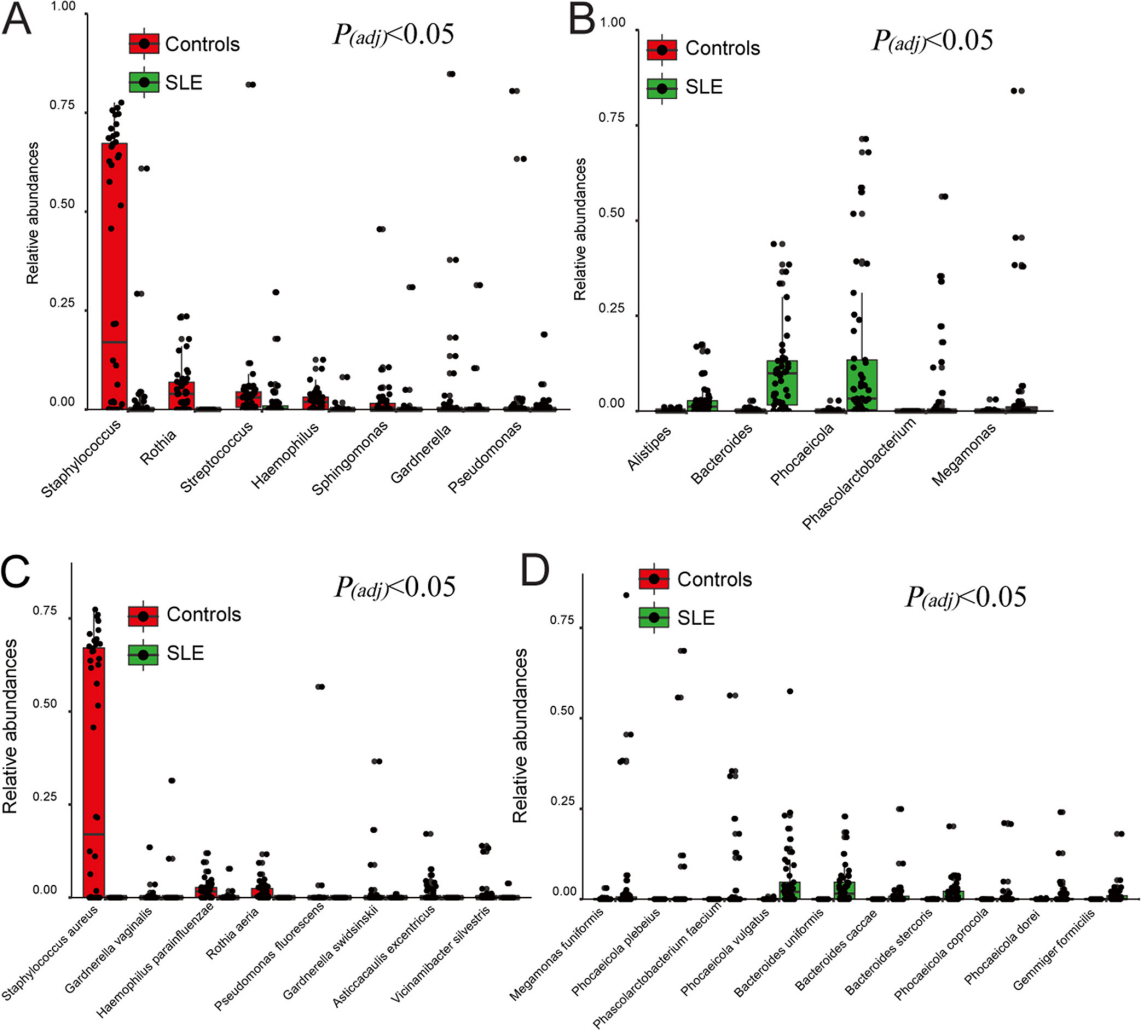
Bacterial genera and species are different between controls and SLE group. (A) Bacterial genera that were more abundant in controls compared to SLE patients (*P*_adj_ < 0.05). *P* value was calculated using Wilcoxon rank-sum test and adjusted by Benjamini and Hochberg false discovery rate (FDR). (B) Bacterial genera that were less abundant in controls compared to SLE patients (*P*_adj_ < 0.05). *P* value was calculated using Wilcoxon rank-sum test and adjusted by Benjamini and Hochberg FDR. (C) Bacterial species that were more abundant in controls compared to SLE patients (*P*_adj_ < 0.05). *P* value was calculated using Wilcoxon rank-sum test and adjusted by Benjamini and Hochberg FDR. (D) Bacterial species that were less abundant in controls compared to SLE patients (*P*_adj_ < 0.05). *P* value was calculated using Wilcoxon rank-sum test and adjusted by Benjamini and FDR.

At the genus level, PCoA based on the Bray-Curtis Dissimilarity Index also revealed differential clustering of bladder microbiomes from controls relative to SLE patients (Fig. S4A; *R*^2^ = 0.153, *P*_adj_ = 0.001). The 15 most abundant genera (>1% average relative abundance of all urine samples) are displayed in Fig. S4B. Among them, seven genera, (Staphylococcus, *Rothia*, Streptococcus, *Haemophillus*, *Sphingomonas*, *Gardnerella*, and Pseudomonas) were significantly more abundant in controls (Fig. 2A; *P*_adj_ < 0.05), especially Staphylococcus, which often predominated. In contrast, five genera (*Alistipes*, *Bacteroides*, *Phocaeicola*, *Phascolarctobacterium*, and *Megamonas*) were significantly more abundant in the SLE group (Fig. 2B; *P*_adj_ < 0.05). However, when we adjusted for the confounding factors, such as nutrient intake and medication usage, using Multivariate Association with Linear Modes (MaAslin) analysis, we found that *Alistipes* and Blautia wexlerae were affected by calcium intake, and B. wexlerae was also affected by prednisone use (Table S8; *P*_adj_ < 0.001); thus, *Alistipes* and B. wexlerae were removed from the downstream interaction analysis. The bacterial species >0.5% average relative abundances of all urine samples are displayed in Fig. S5; 8 species were significantly more abundant in controls, especially S. aureus (Fig. 2C), whereas 10 species were significantly more abundant in the SLE groups (Fig. 2D).

Next, we divided SLE patients into lupus nephritis (LN) and non-LN subgroups (Table S9); the LN and non-LN groups matched demographically, except for age. Based on PCoA, the bladder microbiome of these subgroups did not differ (*R*^2^ = 0.022, *P*_adj_ = 0.377), but both differed from controls (Fig. S6A; *R*^2^ = 0.072, *P*_adj_ = 0.002; *R*^2^ = 0.128, *P*_adj_ = 0.002, respectively). As measured by Shannon’s H Index, microbial diversity also did not differ between these two subgroups, but the SLE patients with LN had more microbial diversity than controls (Fig. S6B; *P*_adj_ < 0.05).

Finally, as a pilot analysis, we compared the bladder microbiome with the vaginal and gut microbiomes using only the subset (*n* = 15) of SLE patients who provided all three sample types. amplicon sequence variant (ASV) features and its corresponding bacterial species in feces, urine, and vagina in SLE patients are shown in Table S10. PCoA based on species showed that the SLE microbiome of the bladder differed from those of the vagina and gut (Fig. S7A; *R*^2^ = 0.105, *P*_adj_ = 0.001). By Bray-Curtis Dissimilarity Index, the SLE bladder microbiome more closely resembled the vaginal microbiome than it did the gut microbiome (Fig. S7B; *P*_adj_ < 0.001, and *P*_adj_ < 0.01, respectively). However, the predominant species in the bladder microbiome were dissimilar to those of both the gut and vagina (Fig. S7C).

### Urinary metabolome is altered in SLE patients.

**FIG 3:**
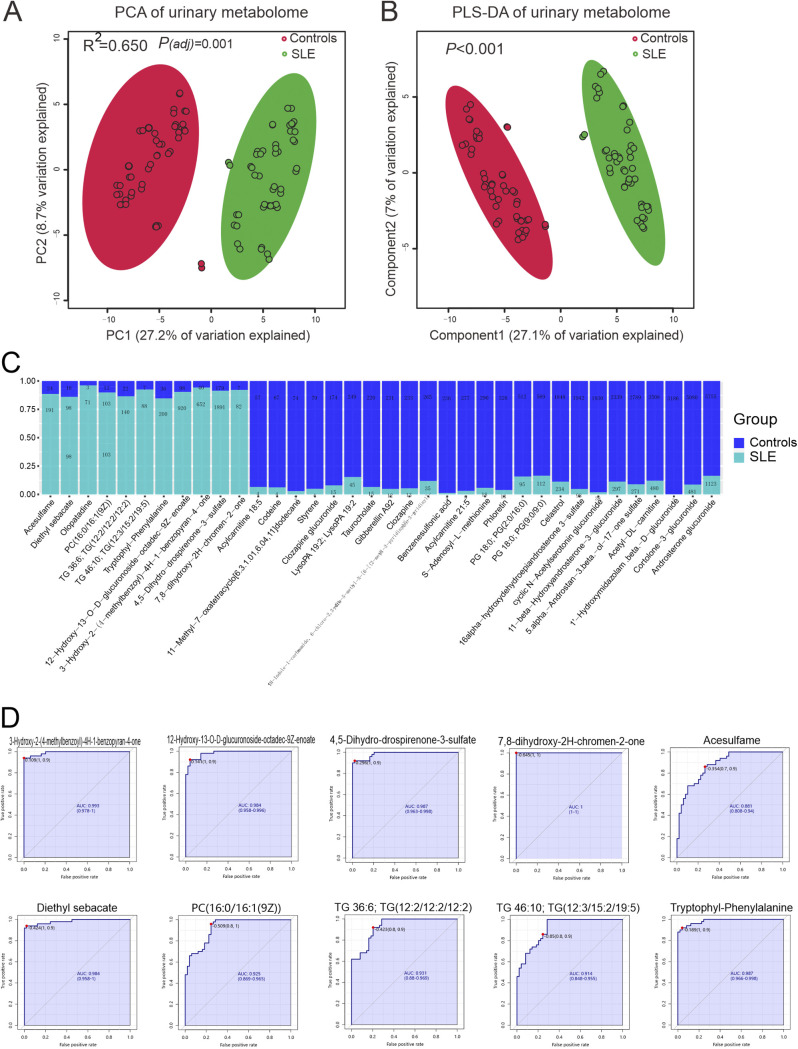
Urinary metabolome differed in SLE patients. (A) Separation of urinary metabolome between patients with SLE and controls, revealed by principal-component analysis (PCA). The explained variances are shown in brackets. Anosim was performed for statistical comparisons of samples in two groups. The 95% confidence ellipse is drawn for each group. (B) Partial least square-discriminant analysis (PLS-DA) plot. Scores plot between the selected PCs. The explained variances are shown in brackets. PERMANOVA was used to test statistical comparisons of ions in SLE and control groups. (C) The metabolites showing significant difference between the control and SLE groups. The metabolites described in the graph met the following criteria: *P*_adj_ < 0.05 in Wilcoxon rank-sum test variable importance in projection (VIP > 1) in PLS-DA; and fold change (FC) >2 or <0.5. (D) Receiver operating characteristic curve (ROC) curve for validation of metabolomic classification of control and SLE patients. Sensitivity is on the *y* axis, and specificity is on the *x* axis. The area-under-the-curve (AUC) is in blue.

To test whether the urinary metabolome differs between SLE patients and controls, we performed untargeted metabolomics on the urine samples. Based on principal component analysis (PCA) using all 1,076 metabolites detected (Table S5), the metabolic composition of SLE patients differed significantly from controls (Fig. 3A; *R*^2^ = 0.650, *P = *0.001). Partial least-squares discriminant analysis yielded similar results (Fig. 3B; *P < *0.001). Also, we tested the effect of medication usage on the metabolome and found no differences between/among the dosage subgroups (Fig. S8A and 8B; *R*^2^ = 0.004, *P = *0.420; *R*^2^ = 0.058, *P = *0.892). These results suggested that the metabolome differed between controls and SLE patients, and the difference was not due to medication.

Of the 1,076 annotated metabolites, 120 metabolites were significantly more abundant in the SLE group, whereas 124 were significantly less abundant (Fig. S9A; *P < *0.05). Among the top 25 most abundant metabolites in the heatmap, 13 were visually less abundant in SLE group (Fig. S9B). After adjusting for confounding factors, including nutrient intake of calcium and zinc, 38 metabolites with variable importance in the projection (VIP) greater than 1 and with fold change less than 0.5 or greater than 2 differed significantly between controls and SLE patients, such as acesulfame, diethyl sebacate, and olopatadine (Table S11 to S14; *P < *0.05, Fig. 3C). To determine whether these 38 metabolites were affected by medication usage, we compared the metabolites according to dosage, and found no difference (Table S15 and S16; *P*_adj_ > 0.05). Since urinary hydroxychloroquine and desethylchloroquine are metabolites of the medication hydroxychloroquine (24), binary regression analysis was used to determine whether they were affected by hydroxychloroquine intake. It showed that hydroxychloroquine intake was a confounding factor of urinary hydroxychloroquine and desethylchloroquine (Table S17); thus, they were removed in the downstream analysis. To look for potential biomarkers that could distinguish SLE from controls, we performed classical eceiver operating characteristic (ROC) curve analysis. From this analysis, 10 metabolites, including acesulfame and diethyl sebacate, had an area under the curve (AUC) value >0.85, indicating they could be biomarkers of SLE (Fig. 3D).

Like the bladder microbiome, the metabolic composition of the LN and non-LN subgroups did not differ, but each differed significantly from the composition of controls (Fig. S10; Controls versus LN, *R*^2^ = 0.255, *P = *0.002; Controls versus non-LN, *R*^2^ = 0.173, *P = *0.002; LN versus non-LN, *R*^2^ = 0.010, *P = *0.909). No metabolites had an AUC >0.85 when we performed classical ROC curve analysis to compare LN and non-LN. However, when we compared the metabolic differences between controls and LN, 427 metabolites were differentially abundant (*P < *0.05); 185/427 had fold change greater than 2 or less than 0.5 and 121/185 metabolites with VIP greater than 1. Next, we performed classical ROC curve analysis to compare LN and controls using the 121 metabolites with VIP >1; we found 53 metabolites that had an AUC >0.85 (Table S18 to S21). When the non-LN and control groups were compared, 239 metabolites were differentially abundant (*P < *0.05), 132/239 metabolites with fold change greater than 2 or less than 0.5, and 101/132 metabolites with VIP greater than 1. Thirty-four metabolites had an AUC above 0.85 when we compared non-LN patients with controls using ROC curve analysis (Table S22 to S25).

### Bladder microbiome was associated with urinary metabolome.

**FIG 4:**
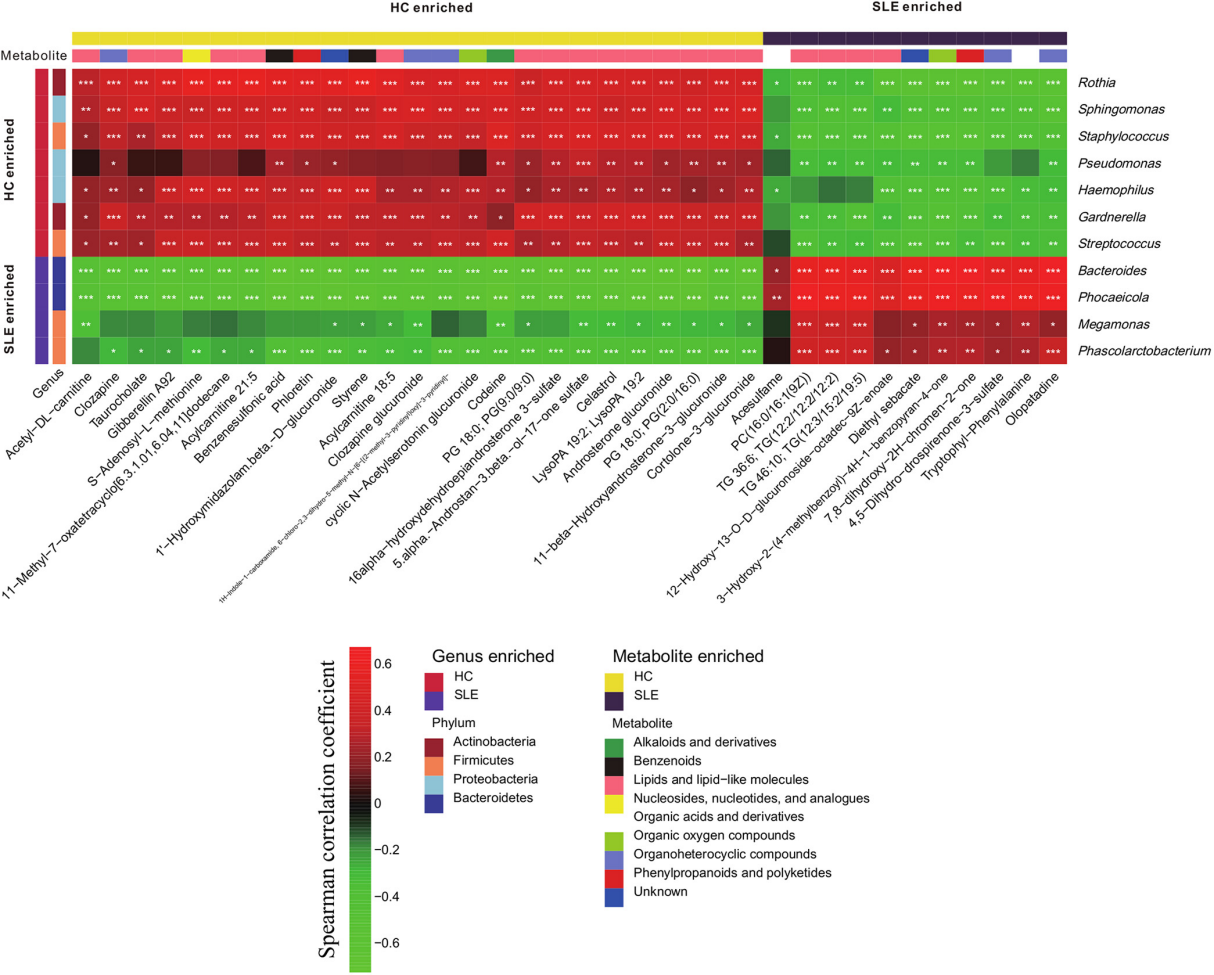
Bladder microbiome was associated with metabolites The heatmap depicted the association between the taxa and metabolites that differ in SLE relative to controls. Spearman correlation analysis was performed on the abundant bacterial genera (>1% average relative abundances) and metabolites that differed between the healthy control (HC) and SLE groups. The correlation of two variables with values of |*r*|>0.3 and *P < *0.05 are displayed. *, *P < *0.05; **, *P < *0.01; and ***, *P < *0.001.

Bladder microbiome and urinary metabolome correlated robustly across all subjects (Fig. S11; M^2^ = 0.906, *P = *0.001). To determine specific associations between the bacterial genera and metabolites, we conducted a Spearman correlation analysis using the abundant bacterial genera (>1% average relative abundances of all urine samples) and metabolites that differed between the SLE and control groups. Indeed, most of the SLE-enriched genera were positively correlated with most of the SLE-enriched metabolites, and most of the SLE-depleted genera were negatively correlated with most of the SLE-enriched metabolites (Fig. 4 and Table S26; |*r*|>0.3, *P < *0.05). For example, the SLE-enriched genera, such as *Bacteroides*, were positively correlated with SLE-enriched metabolites, such as the lipids and lipid-like molecules, including PC [16:0/16:1(9Z)]. Notably, the SLE-enriched genera, including *Bacteroides*, were positively correlated with several SLE-enriched organoheterocyclic compounds, including olopatadine.

### Urine cytokines were altered in SLE patients.

**FIG 5:**
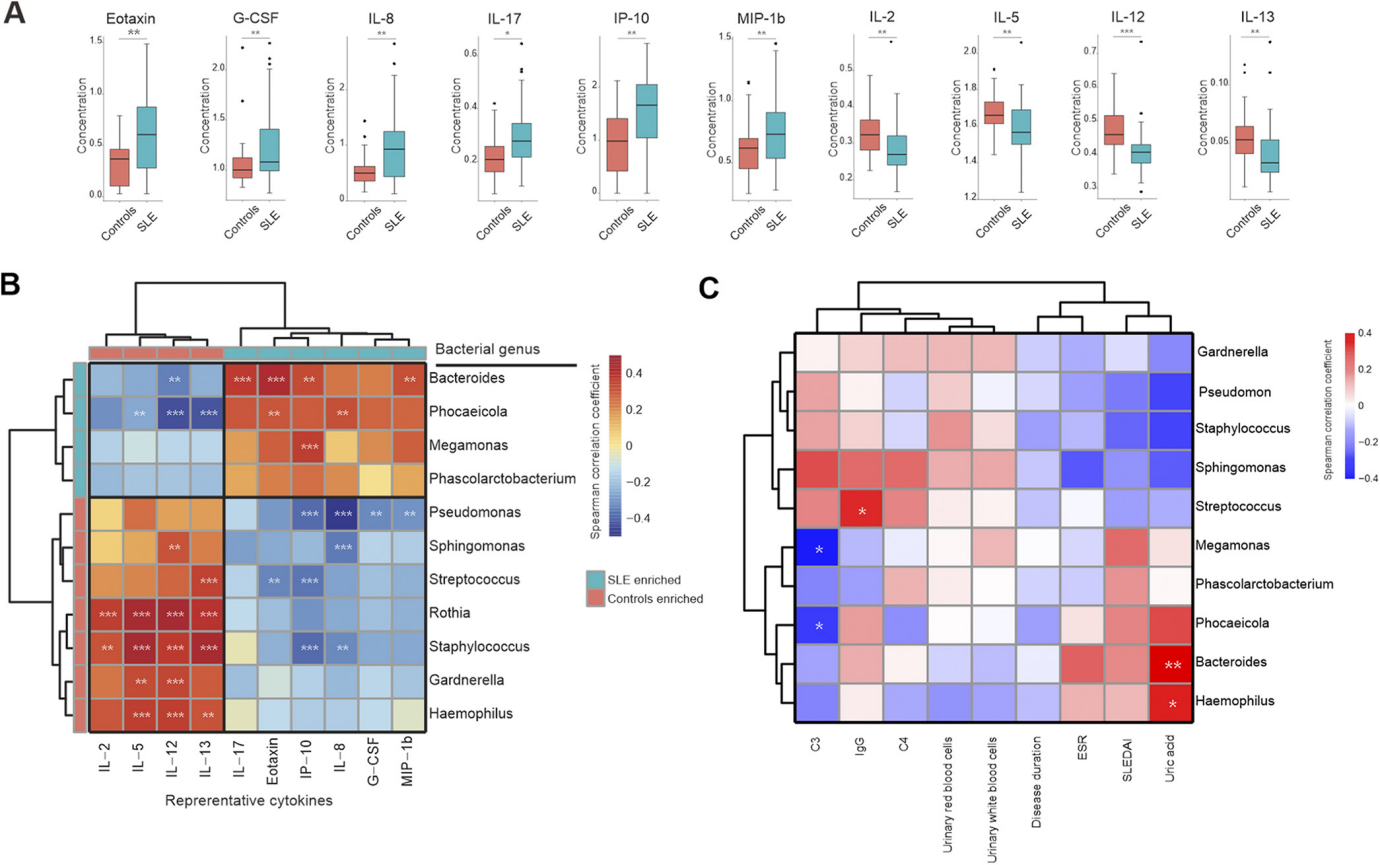
Urinary cytokines and disease profiles in SLE were associated with bladder microbiome. (A) Urinary cytokines increased and decreased in SLE group compared to controls. *P* value was calculated using Wilcoxon rank-sum test and adjusted by Benjamini and Hochberg false discovery rate. (B) Spearman correlation analysis was performed on the most abundant bacterial genera (>1% average relative abundances) and cytokines that differed between the controls and SLE groups. The correlation of two variables with values of |*r*|>0.3 and *P < *0.05 are displayed. *, *P < *0.05; **, *P < *0.01; and ***, *P < *0.001. (C) Spearman correlation analysis was performed on the abundant bacterial genera (>1% average relative abundances) and disease profiles of SLE patients. The correlation of two variables with values of |*r*|>0.3 and *P < *0.05 are displayed. *, *P < *0.05; **, *P < *0.01; and ***, *P < *0.001.

We next tested whether urinary cytokines differed between SLE patients and controls; 26 of the 27 cytokines assessed were identified. Among them, 10 cytokines differed in concentration between SLE patients and controls, including 6 cytokines (eotaxin, granulocyte colony-stimulating factor, IL-8, IL-17, inducible protein-10 [IP-10], and macrophage inflammatory proteins-1b [MIP-1b]) significantly more abundant in SLE patients and 4 cytokines (IL-2, IL-5 IL-12, and IL-13) significantly less abundant in SLE patients (Fig. 5A; *P*_adj_ < 0.05). However, when we compared the cytokines among the controls, LN, and non-LN SLE patients, 12 cytokines, including IL-8, differed significantly between controls and LN SLE patients (Fig. S12; *P*_adj_ < 0.05), and no cytokines differed significantly between controls and non-LN SLE patients.

### Bladder microbiome was correlated to urine cytokines.

To examine the associations between differentially abundant bacterial genera and SLE-linked cytokines, we performed a correlation analysis (Fig. 5B and Table S27; |*r*|>0.3, *P < *0.05). Several of the SLE-enriched bacterial genera were positively associated with several SLE-enriched cytokines. In contrast, the SLE-depleted genera, such as Pseudomonas, were negatively correlated to SLE-depleted cytokines like IL-8.

### Bladder microbiome was associated with SLE-linked disease profiles.

To look for associations between bladder microbiome and SLE-linked disease profiles, we performed a correlation analysis between the bacterial genera with the patients’ disease profiles. The disease profiles were as follows: the duration of SLE and LN, SLEDAI, immunological features, and urinary analysis outcomes that displayed significant differences between controls and SLE group or those only detected in the SLE patients (Table 1). *Megamonas* and *Phocaeicola* were negatively correlated with serum C3, whereas IgG, more abundant in SLE patients, was positively correlated with Streptococcus. *Bacteroides* and Haemophilus were positively correlated with uric acid (Fig. 5C and Table S28, |*r*|>0.3, *P < *0.05).

## DISCUSSION

This is an integrated multi-omics analysis of bladder urine. It shows that the bladder microbiome and urinary metabolome profiles of SLE patients differ significantly from those of controls after adjusting for confounding factors. It also shows that the microbiome profile correlates substantially with urinary metabolome, urinary cytokines, and disease characteristic profiles.

We showed that the bladder microbiome was associated with SLE. Compared with asymptomatic controls, the SLE bladder microbiome was greatly altered with increased microbial diversity and altered abundances of individual taxa at all tested taxonomic levels. The increased diversity of the bladder microbiome is quite substantial, considerably larger than in patients with lower urinary tract symptoms than asymptomatic controls (25, 26). Alterations to the microbiomes of SLE patients have been reported for human gut, oral cavity, and skin (3–15); however, the increased diversity of the SLE bladder microbiome was dissimilar to the findings of previous studies of the SLE gut microbiome, in which patients had lower bacterial diversity than controls (3, 4, 8, 11).

The bladder microbiome of asymptomatic controls, most of whom were women from eastern China (Jiangsu province), was most often predominated by Staphylococcus. We also observed predominance of Staphylococcus over *Lactobacillus* and *Gardnerella* in the bladder of our local women in a previous study (19). This observation was also confirmed in another study that used the expanded quantitative urine culture (EQUC) method to analyze bladder urine samples from 14 asymptomatic adult female controls. In that study, 78.6% (11/14) females had Staphylococcus, while only 14.3% (2/14) had *Lactobacillus* and 14.3% (2/14) had *Gardnerella* (our unpublished data; Table 1). Thus, although *Lactobacillus* predominates in the bladders of most asymptomatic controls of North American women, Staphylococcus predominates in some individuals (27, 28). Different genetic, ethnic, sociocultural, lifestyle, hormonal status and dietary diversity may contribute to differences in the bladder microbiomes of North American and eastern Chinese women.

The phylum *Bacteroidetes*, the genera *Bacteroides* and *Alistipes*, and the species B. uniformis were more common in the SLE group than in the controls, an observation that resembles that of a previous study on the SLE gut microbiome (7). In the gut, certain *Bacteroidetes* spp. are thought to be probiotic, as they can improve some intestinal disorders, as well as cardiovascular disease, behavior disorders and cancer (29). For example, the supplementation of B. uniformis can increase the production of butyrate and restore the proportion of induced intraepithelial lymphocytes and type-3 innate lymphoid cells in the intestinal epithelium (30). Further investigation of the role of *Bacteroidetes* in the human bladder is desirable.

LN is a form of glomerulonephritis that constitutes one of the most severe organ manifestations in SLE patients. Previous human and animal gut microbiome studies demonstrated that LN is associated with microbiome composition (4, 31). Thus, we divided the patients into LN and non-LN subgroups. Although there was no significant difference in bacterial composition or abundance between LN and non-LN patients, microbial diversity differed between LN patients and controls, indicating there might be an interaction between bladder microbiome and inflammation in patients’ kidneys. However, the group sizes were too small and too biased to draw a strong conclusion.

The urinary metabolome also differed significantly in our study. Previous studies also showed that the urinary metabolome was altered in SLE and LN patients (32, 33), but those studies analyzed voided urine, which often contains posturethral contamination (34). We assessed catheterized urine that avoided that contamination (34), and identified several metabolites altered in SLE patients. For example, organoheterocyclic compounds, including olopatadine, were more abundant in SLE patients. Olopatadine appears to have significant anti-inflammatory activity, and inhibits histamine release from mast cells (35). Thus, the increase of olopatadine in urine might play a potential protective role for SLE patients.

Based on ROC, 10 metabolites differed between SLE patients from controls, 53 metabolites differed between LN patients and controls, 34 metabolites differed between non-LN patients and controls, but no metabolite differed between LN and non-LN patients Thus, urinary metabolites could be valuable for diagnosis of SLE as an alternative to the usual classification of SLE by serum autoantibody assessment (36). They also might help predict the severity of kidney damage in SLE. Future large prospective studies will be needed to determine the validity of these results.

Like the microbiome and metabolome profiles, bladder cytokines differed between SLE patients and controls. For example, IL-8 was significantly more abundant in SLE patients, consistent with previous cytokine studies of SLE patients (37, 38). As the kidney is affected by SLE and evaluation of urinary cytokines is reported to predict development of renal flares (39), we compared the urinary cytokines among controls and both LN and non-LN SLE patients. No cytokines differed between controls and non-LN SLE patients. In contrast, 10/12 of the cytokines that differed between controls and LN SLE patients also differed between controls and the entire group of SLE patients. The frequently confirmed LN-associated cytokines, such as IL-17 (40), IP-10, and MCP-1 (41), were only more abundant in the LN group compared to controls. These findings suggest that kidney damage in SLE patients may be responsible for altered bladder cytokine expression.

As integration of microbiome and metabolome data possess the potential to identify microbial influence on host physiology through production, modification, and/or degradation of bioactive metabolites, we performed correlation analysis on the bladder microbiome and metabolome data. A major finding of the present study was the positive association between *Bacteroides* and organoheterocyclic compounds including olopatadine. Olopatadine is reported to be an antihistamine agent with inhibitory activities against chronic inflammation (42). *Bacteroidetes* also was positively correlated to PC [16:0/16:1(9Z)], which has been reported to have anti-inflammatory properties (43). Thus, the interaction between *Bacteroides* and organoheterocyclic compounds in the bladder might play a potential role in inhibiting inflammation-associated metabolites in SLE.

*Phocaeicola* was increased in SLE patients, but negatively associated with C3 levels. A recent study suggested that *Phocaeicola* was responsible for colorectal cancer in the gut (44). Thus, it might also play a potential pathological role in SLE patient’s bladder.

The microbiome plays a vital role in the regulation of host mucosal inflammation. To investigate interactions between bladder microbes and the inflammation response, we performed the integration analysis between the altered bacterial genus and urinary cytokines in patients. The SLE-enriched genus *Bacteroides* was positively correlated to the SLE-enriched cytokines, including IP-10 and IL-17, which are usually used as biomarkers to predict disease severity (40, 45). We also noticed that Pseudomonas was negatively correlated to IL-8, which can induce chemotaxis in target cells, and cause neutrophils and granulocytes to migrate toward the inflammation site (46). In the human respiratory tract, IL-8 helps generate dense neutrophil accumulations in bronchopulmonary infections caused by Pseudomonas aeruginosa (47). Thus, the negative interaction between Pseudomonas and IL-8 suggests that certain species in the bladder might play different roles from other members of the same species in other body sites. In our study, IL-8 was elevated only in LN patients and not non-LN patients, which fits with these IL-8 functions. Therefore, Pseudomonas might play a role in regulating immunity in bladder microbial communities. The correlations between bladder microbiome and urinary cytokines indicate there may be a microbe-inflammation axis that should be explored.

It is known that complement deficiency is associated with SLE, predisposing these patients to infection (48). Indeed, the SLE patients in this study had decreased complement C3 and C4 levels and a negative correlation between C3 and SLE-enriched bacterial genera, including *Megamonas*. In contrast, *Megamonas* has been reported to be depleted in the gut of SLE patient and positively correlated to Th17 (49). These observations warrant further investigation of *Megamonas* in SLE pathology. In addition, the SLE-depleted genus, Streptococcus, was positively correlated to immunoglobulin G (IgG), a major serum immunoglobulin principally responsible for elimination of pathogens and toxic antigens (50). Uric acid accumulation is common in SLE patients (51); it was elevated in our study, and positively correlated with *Bacteroides* and Haemophilus. Based on these findings, we hypothesize the possible existence of a host-microbe interaction in the human bladder that contributes to the SLE phenotype.

Individuals with SLE are regularly treated with immunosuppressives, which can cause serious adverse effects that severely compromise life quality. Therefore, there is an urgent need to control the disease process. Our present study demonstrated that the bladder microbiome of SLE patients is associated with their urinary metabolites, cytokines, and disease profiles, highlighting plausible disease-specific mechanisms for future investigation.

Our study has several limitations. Given frequent infection in SLE patients, it was hard to recruit volunteers who were willing to accept urinary catheterization (which is an invasive procedure); therefore, our sample size was small and entirely recruited at a single center with participants from eastern China. Moreover, as females are more susceptible to SLE, only a few males were recruited to this study. Thus, we could not compare sexual differences. The small sample size also makes it difficult to interpret the differences observed between controls and LN patients relative to non-LN patients. A study with a larger sample size that includes more males and participants from different areas of China should be conducted in the future.

## MATERIALS AND METHODS

### Participant recruitment and assessment.

Fifty SLE patients, who fulfilled at least 4 of the American College of Rheumatology Criteria for the diagnosis of SLE (52), and 50 sex-, age-, BMI-, and co-morbid disease-matched controls, were consecutively recruited from Wuxi Second Affiliated Hospital of Nanjing Medical University (Fig. S1). The inclusion and exclusion criteria are described in File S1. Disease activity was measured using SLEDAI score (53). All participants signed their informed consent before sample collection. The study was executed in accordance with the Ethical Committee of the hospital (ref. 201805). Fifty asymptomatic controls were recruited prospectively. When 20 SLE patients had been recruited, we began recruiting asymptomatic controls, making sure to match the SLE cohort participants’ age, gender, and BMI.

Food assessment was performed as described previously (54); the method is described briefly in File S2. Disease treatment was collected from the patient’s medical record and via a face-to-face interview.

### Urine sample collection.

Urine samples were collected through a urinary catheter. Before insertion of the catheter, 5% iodophor was applied to sterilize the genital and perineal areas. The collected urine was separated into four portions, which were used for detecting or measuring the bladder microbiome, metabolome, creatinine levels, and cytokines. Fecal and vaginal samples were collected before the collection of urine samples. Fecal material was collected in a sterile container by the patient, and 30 mg was immediately placed in a sterile container. The vaginal sample was obtained by a nurse using a sterile, DNA-free swab inserted into the middle section of vaginal tract. All urine, feces and vaginal samples were placed in sterile, DNA- and enzyme-free centrifuge tubes and immediately stored at −80°C until use.

### DNA extraction and bioinformatics analysis.

30 mL urine samples were processed for sequencing as described in File S3. The DNA extraction from the vaginal and fecal samples were the same as urine samples. We used DADA2 (https://github.com/benjjneb/dada2) to process reads derived from 16S rRNA V3-V4 region, including quality control [truncQ = 8, maxN = 0, maxEE = c(2)], dereplication, merging forward and reverse reads (trimOverhang = TRUE, minOverlap = 5), and chimera removal (method = “consensus”) to obtain ASVs. To identify contaminating taxonomic signals from the kit and environment, we sequenced the urinary catheter, tubes, lysis buffer, PCR buffer, and water samples used for urine collection, DNA preparation, and sequence. To obtain taxonomic identities, we combined BLCA with the NCBI 16S Microbial Database to obtain species-level resolution from V3 to V4 amplicons, as reported recently (55). Taxa with a confidence score >60 for used for downstream analyses. To remove contaminating sequences, we manually removed bacterial species whose counts did not exceed five times the maximum number of counts in the controls. After decontamination, urine samples with <1000 counts were considered below the level of detection and removed from downstream analyses. The crude DADA output is included in Table S1, and the filtered taxa of bacterial phylum, genus, and species are listed in Table S2 to S4. MaAslin framework was used to adjust the effects of confounding factors.

### Urinary metabolome profiling and processing.

Urinary metabolome profiling was performed using liquid chromatography tandem mass spectrometry, liquid chromatography-tandem mass spectrometry (ExionLC and TripleTOF 5600, SCIEX, Framingham, MA, USA) as previously described (see File S4). In total, 6,770 and 6,078 peaks were detected in the positive and negative ionization modes, respectively; 6,515 (positive mode) and 5,970 (negative mode) metabolite features remained. The positive-mode and negative-mode features were then annotated using The Kyoto Encyclopedia of Genes and Genomes (KEGG; https://www.genome.jp/kegg/) and Human Metabolome Database (HMDB; http://www.hmdb.ca). The result was 1,076 annotated metabolites (Table S5).

### Cytokines and creatinine detection.

The Bio-Plex 200 System (Bio-Rad) and Bio-Plex Pro Human Cytokine 27-plex assay (Bio-Rad, CA, USA) were used to detect urinary cytokines. Creatinine was detected using the human urine Elisa kit of creatinine (Hengyuan Biological Technology Co., Ltd, Shanghai, China).

### Disease profile measurement.

Blood samples were collected on the day of urine sample collection. The immunoturbidimetric test was used to assess serum complement (C) and immunoglobulin antibodies (AU5421; Beckman Coulter, USA). Immunoblotting and immunofluorescence were used to detect serum autoantibodies. Erythrocyte sedimentation rate (ESR) was determined by the Westergren method (XC-408 ESR Monitor; Mindray, China).

Lupus nephritis (LN) was defined as clinical and laboratory manifestations that meet American College of Rheumatology criteria (56). Systemic Lupus Erythematosus Disease Activity Index 2000 (SLEDAI-2K) was used to assess disease severity (53).

### Statistical analysis.

For microbiome analysis, Beta-diversity analysis (Bray-Curtis dissimilarity) of microbial communities was performed at species-, genus- and phylum-levels using ‘vegdist’ function with the “bray” mode, and permutational multivariate analysis of variance was calculated using the ‘adonis’ function. Alpha-diversity analysis (Shannon’s H) was performed at the species level using the ‘diversity’ function with the “shannon” mode in R. For metabolome analysis, the concentration of urinary metabolites was adjusted for variability in urine dilution, using creatinine as a normalization indicator. For the comparison of the metabolite intensity between SLE and controls, we performed statistical analysis using MetaboAnalyst 5.0 (https://www.metaboanalyst.ca). Metabolites with (i) variable importance in the projection (VIP) greater than 1, (ii) fold change greater than 2 or less than 0.5, and (iii) *P*-value less than < 0.05, were then log_2_ transformed and subjected to linear model analysis to control for confounding factors, including nutrient intake (Binary logistic regression model analysis, SPSS 24.0). Classical ROC curve analysis, including logistic regression analysis with selected variables to obtain modeling results and to compare the performance using the accuracy/performance plots (i.e., area under the curve, specificity, and sensitivity) was used to evaluate the performance of single metabolites. For cytokine analysis, creatinine was used to normalize urinary cytokine concentration.

Pearson’s Chi-square or Fisher’s exact tests were used with categorical variables; Student’s *t* test was used on normalized continuous variables and Wilcoxon rank-sum test on nonnormal continuous variables. The *P* value was adjusted for multiple comparisons using the Benjamini-Hochberg false discovery rate.

### Data accession.

Raw data from 16S rRNA sequencing are available in the Sequence Read Archive under BioProject ID SRP367734 (https://www.ncbi.nlm.nih.gov/bioproject/PRJNA823507). The metabolome data are available in the link: https://ngdc.cncb.ac.cn/omix/select-edit/OMIX001294.
