# Supplementary material for: The Bladder Microbiome, Metabolome, Cytokines, and Phenotypes in Patients with Systemic Lupus Erythematosus
Source: Microbiol Spectr. 2022 Aug 1;10(5):e00212-22. doi: 10.1128/spectrum.00212-22 (PMC9620774; doi:10.1128/spectrum.00212-22)
Supplement: Supplemental Material FOR publication — Files S1-S4, Fig. S1-S12, Tables S1-S28. Download spectrum.00212-22-s0001.pdf, PDF file, 7.4 MB [file spectrum.00212-22-s0001.pdf]

File S1 Participant's inclusion and exclusion criteria

As previous studies using large populations demonstrated that the standard treatment options of low dosages of prednisone and hydroxychloroquine are not associated with gut microbiome composition(1, 2). patients using low dosages of these two medications were included. The exclusion criteria for both cohorts were presence of other autoimmune diseases, pregnancy, breastfeeding, menstruation, severe illness or infections, neoplastic disease, or use of antibiotics, probiotics, vitamin D, vitamin B12, calcium, oral contraceptive, metformin, or proton pump inhibitors within the 2 weeks prior to participation in the study. Night shift subjects also were excluded. At the time of sample collection, controls declared that they had no health problems and that they were not taking medications.

REFERENCES

1. Jackson M, Verdi S, Maxan M, Shin C, Zierer J, Bowyer R, Martin T, Williams F, Menni C, Bell JT, Spector T, Steves C. 2018. Gut microbiota associations with common diseases and prescription medications in a population-based cohort. Nat Commun 9:2655. 10.1038/s41467-018-05184-7.
2. Zhernakova A, Kurilshikov A, Bonder M, Tigchelaar E, Schirmer M, Vatanen T, Mujagic Z, Vila A, Falony G, Vieira-Silva S, Wang J, Imhann F, Brandsma E, Jankipersadsing S, Joossens M, Cenit M, Deelen P, Swertz M, Weersma R, Feskens E, Netea M, Gevers D, Jonkers D, Franke L, Aulchenko Y, Huttenhower C, Raes J, Hofker M, Xavier R, Wijmenga C, Fu J. 2016. Population-based metagenomics analysis reveals markers for gut microbiome composition and diversity. Science 352:565-569. 10.1126/science.aad3369.

File S2 Nutrient intake

A Chinese version of the food frequency questionnaire (FFQ) was used to assess food intake during a face-to-face interview (1). The participants were asked to recall their food and water intake patterns over the previous year (up to the day of urine sample collection). The name and amount consumed food was assessed as was the intake of nutrition supplements. Haoyingyang software v. 2.08.07 (Pinwang Inc. ShenZhen, China) was used to convert the amount of food intake into daily nutrient intake.

REFERENCES

1. Zhao WH, Huang ZP, Zhang X, He L, Willett W, Wang JL, Hasegawa K, Chen JS. 2010. Reproducibility and validity of a chinese food frequency questionnaire. Biomed Environ Sci:1-38.

File S3 DNA extraction and sequence

Each 30 mL urine sample was centrifuged at 10,000×g for 30min at 4°C. Most of the supernantant fluid was decanted, and the pellet resuspended in the remaining 1 mL of urine was transferred to a 1.5 mL Eppendorf tube at 10,000×g for 30 min at 4°C. The supernatant was gently removed and 500 μL lysis buffer (Guhe, Hangzhou, China) was added. The samples were repeatedly frozen and thawed in liquid nitrogen and a 65°C water bath for 3 cycles. AMpure XP magnetic beads (Beckman Coulter, Indianapolis, IN, USA) were used to isolate bacterial DNA. Specifically, 40 μL Agencourt AMPure XP (Beckman Coulter, USA) was added to 80 μL of the urine pellet, vortexed for 30 sec, and incubated for 5 min at room temperature. The tube was placed into a magnetic separator for 5 min, and DNA was bound to magnetic beads, which were drawn to the wall of the microcentrifuge tube. The supernatant was carefully removed without disrupting the magnetic beads. The sample was washed twice with 180 μL 80% ethanol for 30 sec, being placed on a magnet separator between each washing. The purified DNA was eluted with 30 μL elution buffer for 1 min. The beads, now released from the DNA, were collected with the magnet. The DNA-containing supernatant was transferred to a clean tube. Two samples with sterile deionized water were used as negative controls to assess the reagent contamination at this step.

The bacterial 16S rRNA V3-V4 region was PCR-amplified using the universal primers 319F and 806R with 32 cycles. Two samples of sterile water were used to assess reagent contamination. The quantity and quality of the extracted DNA were measured using a NanoDrop ND-1000 spectrophotometer (Thermo Fisher Scientific, Waltham, MA, USA) and agarose gel electrophoresis, respectively. PCR amplicons were purified with Agencourt AMPure XP Beads (Beckman Coulter, Indianapolis, IN, USA) and quantified using the PicoGreen dsDNA Assay Kit (Invitrogen, Carlsbad, CA, USA). Amplicons were then pooled in equal amounts for sequencing using the Illlumina MiSeq platform samples. Two samples without PCR product were sequenced to assess cross contamination.

As low bacterial DNA biomass in urine samples, we assessed potential bacterial

and DNA contamination in the environment using 21 negative samples. They were as follows: air in the biosafety cabinet, various tubes used in the sample collection and DNA isolation, urinary catheter, and reagent. Each type was collected using three samples. For each instrument used in our study, triplicate samples were sequenced as negative controls. For example, three urinary catheters were placed in a sterile cell culture plate. A sterile scissors was used to cut a 1 mM portion of catheter, which was placed in 500 μL lysis buffer. The lysis buffer containing catheters was then processed as described for urine samples. The negative samples were processed with the urine samples immediately and were not exposed biosafety cabinet air for a long time.

File S4 Urinary metabolites processing

Urinary metabolome profiling was performed using liquid chromatography tandem mass spectrometry, LC-MS/MS（ExionLC and TripleTOF 5600, SCIEX, Framingham, MA, USA） as previously described (1). Briefly, 20 μL urine was used for metabolite extraction with 120 μL of precooled 50% methanol. After vortexing for 1 min, the extraction mixture was incubated at room temperature for 10 min and then stored overnight at -20°C. After centrifugation at 4,000 × g for 20 min, the supernatants were transferred into 96-well plates and then stored at -80°C prior to LC-MS analysis. Meanwhile, pooled quality control samples were prepared by combining 10 μL of each extraction mixture. Features detected in less than 50% of QC samples or 80% of the urine samples were removed, and the remaining peaks with missing values were imputed with the k-nearest neighbor algorithm to further improve data quality.

REFERENCES

1. Xiang S, Ye K, Li M, Ying J, Wang H, Han J, Shi L, Xiao J, Shen Y, Feng X, Bao X, Zheng Y, Ge Y, Zhang Y, Liu C, Chen J, Chen Y, Tian S, Zhu X. 2021. Xylitol enhances synthesis of propionate in the colon via cross-feeding of gut microbiota. Microbiome 9. 10.1186/s40168-021-01029-6.

Supplementary Figures

Fig. S1 Overview of the study design.

Fig. S2 Medication usages in patients didn't affect bladder microbiome.

A. PCoA based on Bray-Curtis distances at species level did not show different microbial compositions between SLE patients taking hydroxychloroquine dosages of 0.2 mg/d and those taking 0.4 mg/d. Permutational multivariate analysis of variance (PERMANOVA) was performed for statistical comparisons of samples using different levels of hydroxychloroquine. *P* value was adjusted by Benjamini and Hochberg false discovery rate.

B. PCoA based on Bray-Curtis distances at species level did not show different microbial compositions among SLE patients taking prednisone of 0 mg/d, 5 mg/d and those taking 10 mg/d. PERMANOVA was performed for statistical comparisons of samples using different levels of prednisone. *P* value was adjusted by Benjamini and Hochberg false discovery rate.

Fig. S3 Bacterial evenness and richness of bacterial diversity.

A. Comparison of bacterial species diversity indicator of evenness between control and SLE groups. Permutational multivariate analysis of variance (PERMANOVA) was performed for statistical comparisons of samples in two groups. *P* value was adjusted by Benjamini and Hochberg false discovery rate. ** indicates $P_{(adj)}<0.01$.

B. Comparison of bacterial species diversity indicator of richness between control and SLE groups. PERMANOVA was performed for statistical comparisons of samples in two groups. *P* value was adjusted by Benjamini and Hochberg false discovery rate.

Fig. S4 Bacterial communities and composition at bacterial genus level.

A. PCoA based on Bray Curtis distances at the genus level showed different microbial compositions between control and SLE groups. Permutational multivariate analysis of variance (PERMANOVA) was performed for statistical comparisons of samples in two groups. *P* value was adjusted by Benjamini and Hochberg false discovery rate.

B. Heatmap of bacterial genera. The 15 abundant genera (>1% average relative abundances) were displayed.

Fig. S5 Bacterial composition at bacterial species level.

The heatmap displays the bacterial species >0.5% average relative abundances.

Fig. S6 Comparison of bladder microbiome among control, LN and non-LN groups.

A. Bacterial communities among controls, LN and non-LN SLE patients. PCoA based on Bray Curtis distances at species level was performed. Permutational multivariate analysis of variance (PERMANOVA) was performed for statistical comparisons of samples in two groups. *P* value was adjusted by Benjamini and Hochberg false discovery rate.

B. Bacterial diversity measured by Shannon index was calculated at the bacterial species level. Wilcoxon rank-sum test and adjusted by Benjamini and Hochberg false discovery rate (FDR). ** indicates $P_{(adj)}<0.01$.

Fig. S7 Comparison of microbiome in bladder, vagina, and gut in SLE patients.

A. PCoA based on Bray-Curtis distances at species level showed different microbial compositions between the bladder, vagina, and gut. (PERMANOVA) was performed for statistical comparisons of samples in two groups. *P* value was adjusted by Benjamini and Hochberg false discovery rate.

B. Bray-Curtis dissimilarities of the different niches were calculated using the same SLE patient. Wilcoxon rank-sum test and adjusted by Benjamini and Hochberg false discovery rate (FDR). **, *** indicate $P_{(adj)}<0.01$ and $P_{(adj)}<0.001$, respectively.

C. Microbial profiles of the bladder, gut, and vagina at the species level. Bacterial species abundance with >0.05 % average relative abundances are displayed. On the x axis, the numbers 1 to 15 represent SLE patients with bladder urine, gut and vaginal samples sequenced.

Fig. S8 Medication usages in SLE patients didn't affect urinary metabolome.

A. Principal component analysis (PCA) was used to compare urinary metabolome between SLE patients taking hydroxychloroquine dosages of 0.2 mg/d and those taking 0.4 mg/d. The explained variances are shown in brackets. Anosim was used to calculate R^2 and P value. The 95% confidence ellipse is drawn for each group.

B. Principal component analysis (PCA) was used to compare urinary metabolome among SLE patients taking prednisone of 0 mg/d, 5 mg/d, and 10 mg/d. Anosim was performed for statistical comparisons of samples in two groups. The 95% confidence ellipse is drawn for each group.

Fig. S9 Metabolome comparison between control and SLE groups.

A. Volcano plot. Volcano plot of differential metabolites classification of the control and SLE groups. Metabolites with FDR <0.05 obtained by non-parametric tests and fold change (FC) >2 were identified as significantly different between the two groups. Colored plots indicate upward trend and downward trend of metabolites, and gray plots indicate that they are not statistically significant.

B. Clustering result shown as heatmap. Distance was measured using Euclidean, and clustering algorithm was calculated using Ward's method.

Fig. S10 Metabolome comparison among control, LN and non-LN SLE groups. Metabolome among controls, LN and non-LN SLE patients were compared using principal component analysis (PCA). Anosim was performed for statistical comparisons of samples between groups. The 95% confidence ellipse is drawn for each group.

Fig. S11 The relationship between bladder microbiome and urinary metabolome. Procrustes analysis analyzed the congruence of two-dimensional shapes produced from superimposition of principal component analyses from the datasets of microbiome and metabolome. Euclidian distances of eigenvalues for both the microbiome and metabolome using the Procrustes function in the vegan R package. Longer lines on Procrustes plots indicate more within-subject dissimilarity of the microbiome and metabolome. Significance value shown was calculated using the protest function from the vegan R package.

Fig. S12 Urinary cytokines differed in LN SLE patients comparing to controls. Comparison of urinary cytokines between controls and LN SLE patients. *P* value was calculated using Wilcoxon rank-sum test and adjusted by Benjamini and Hochberg false discovery rate. *, **, *** indicate $P_{(adj)}<0.05$, $P_{(adj)}<0.01$ and $P_{(adj)}<0.001$, respectively.

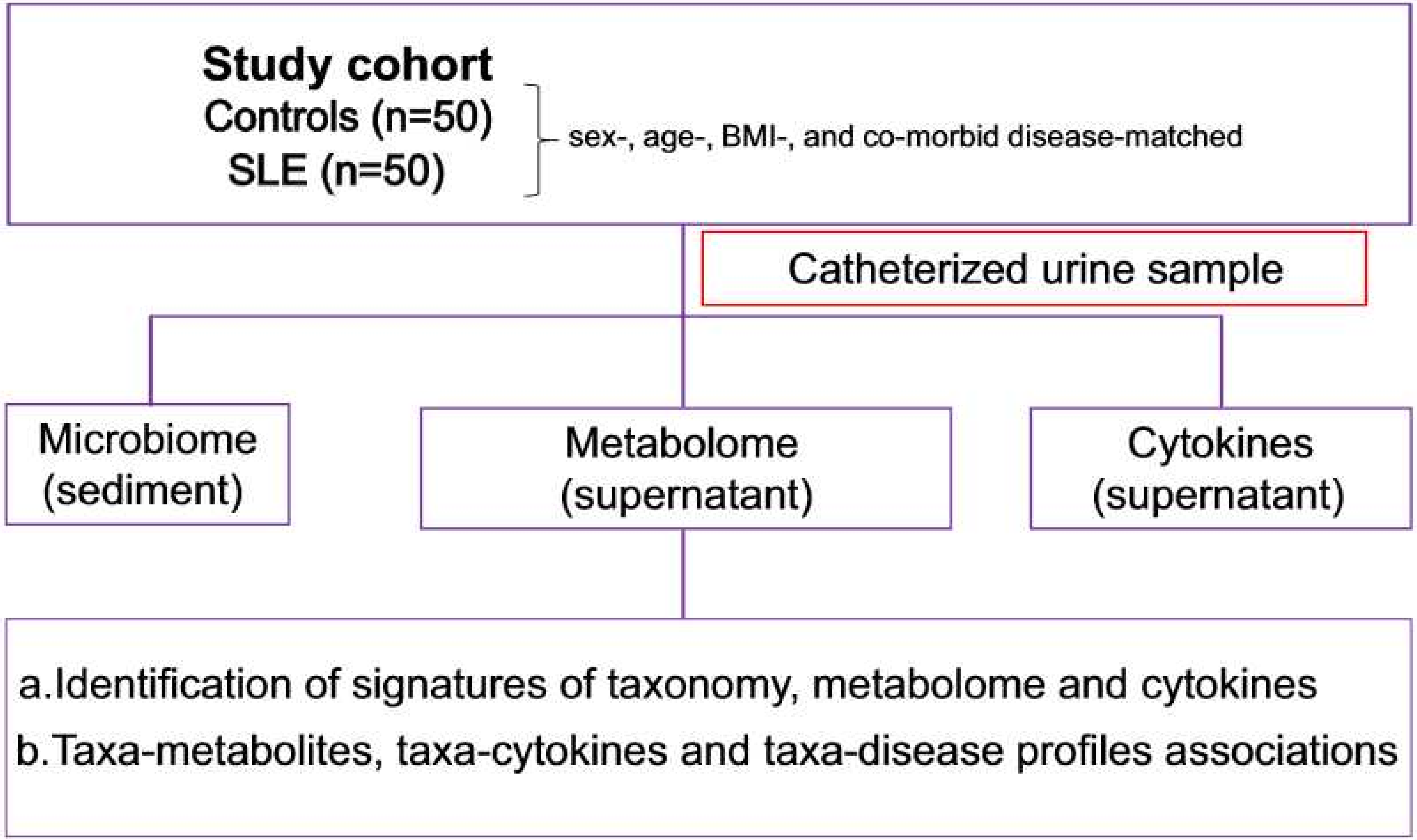
Study cohort
Controls (n=50)
SLE (n=50)
sex-, age-, BMI-, and co-morbid disease-matched
Catheterized urine sample
Microbiome (sediment)
Metabolome (supernatant)
Cytokines (supernatant)
a.Identification of signatures of taxonomy, metabolome and cytokines
b.Taxa-metabolites, taxa-cytokines and taxa-disease profiles associations

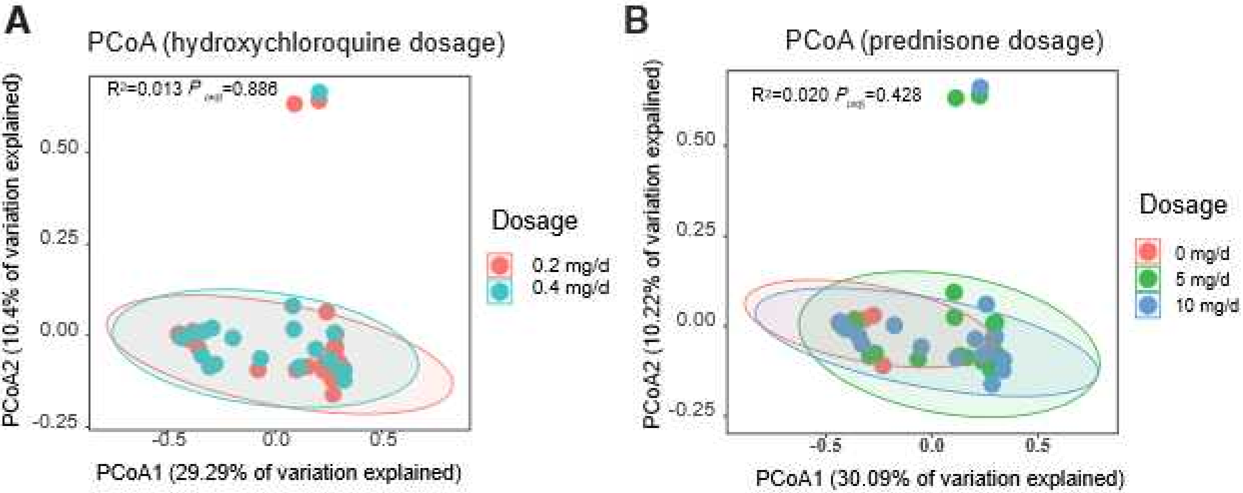
A
PCoA (hydroxychloroquine dosage)
R²=0.013 P(adj)=0.888
0.50
0.25
0.00
-0.25
-0.5
0.0
0.5
PCoA2 (10.4% of variation explained)
PCoA1 (29.29% of variation explained)
Dosage
0.2 mg/d
0.4 mg/d
B
PCoA (prednisone dosage)
R²=0.020 P(adj)=0.428
0.50
0.25
0.00
-0.25
-0.5
0.0
0.5
PCoA2 (10.22% of variation explained)
PCoA1 (30.09% of variation explained)
Dosage
0 mg/d
5 mg/d
10 mg/d

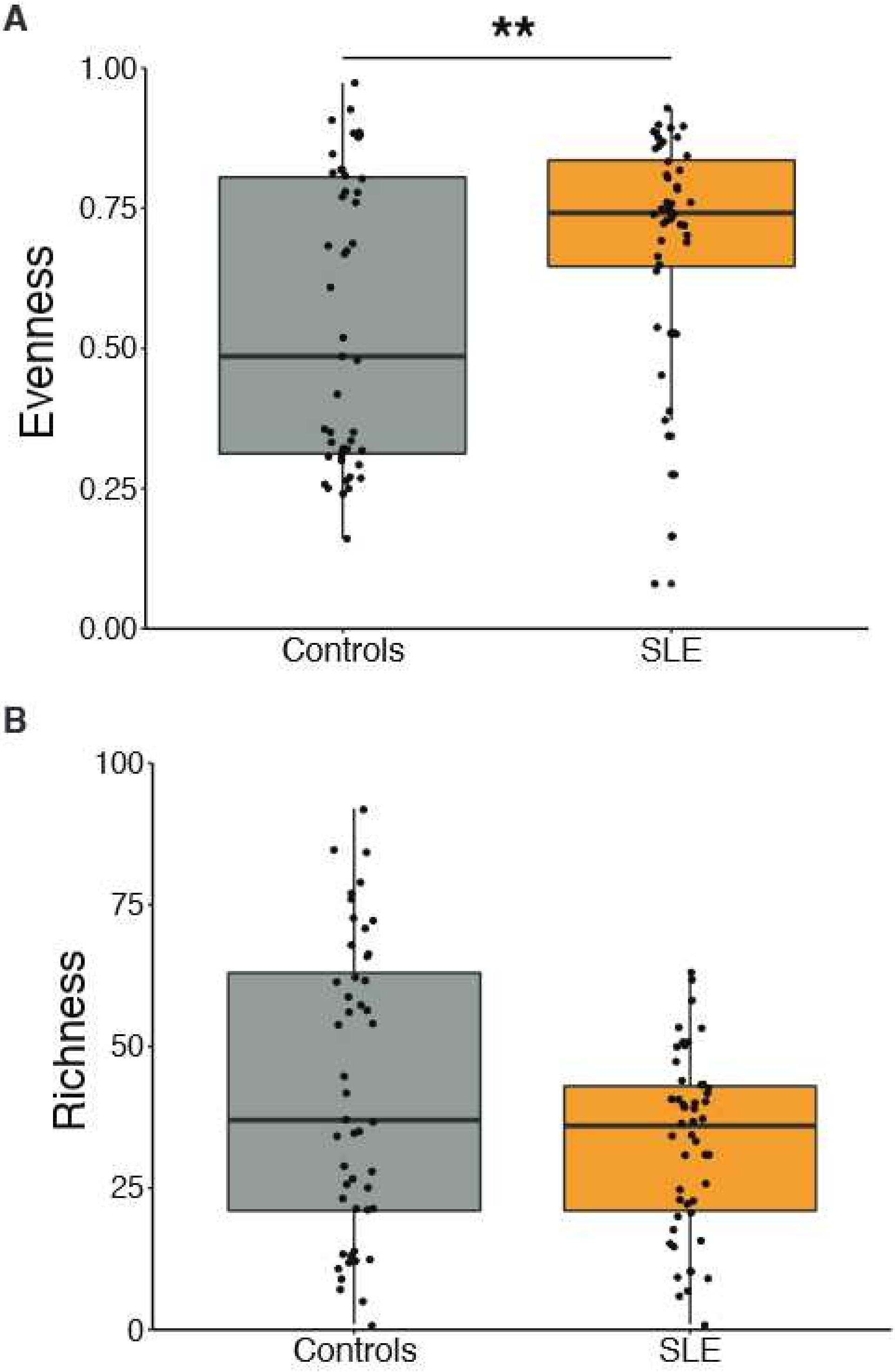

A
**
1.00
0.75
0.50
0.25
0.00
Evenness
Controls
SLE
B
100
75
50
25
0
Richness
Controls
SLE

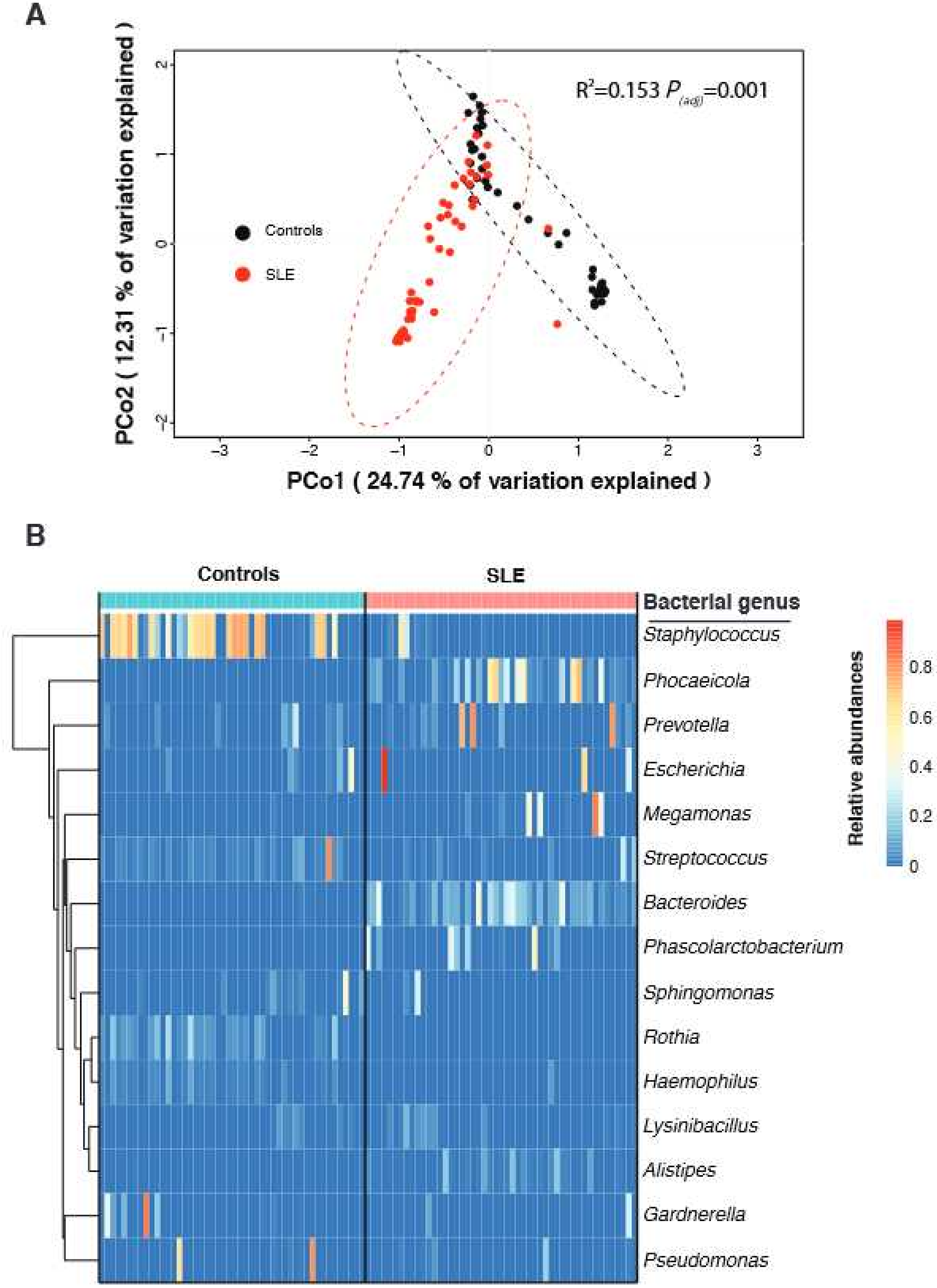
A
R^2=0.153 $P_{(adj)}$=0.001
Controls
SLE
PCo2 (12.31 % of variation explained)
PCo1 (24.74 % of variation explained)
B
Controls
SLE
Bacterial genus
Staphylococcus
Phocaeicola
Prevotella
Escherichia
Megamonas
Streptococcus
Bacteroides
Phascolarctobacterium
Sphingomonas
Rothia
Haemophilus
Lysinibacillus
Alistipes
Gardnerella
Pseudomonas
Relative abundances

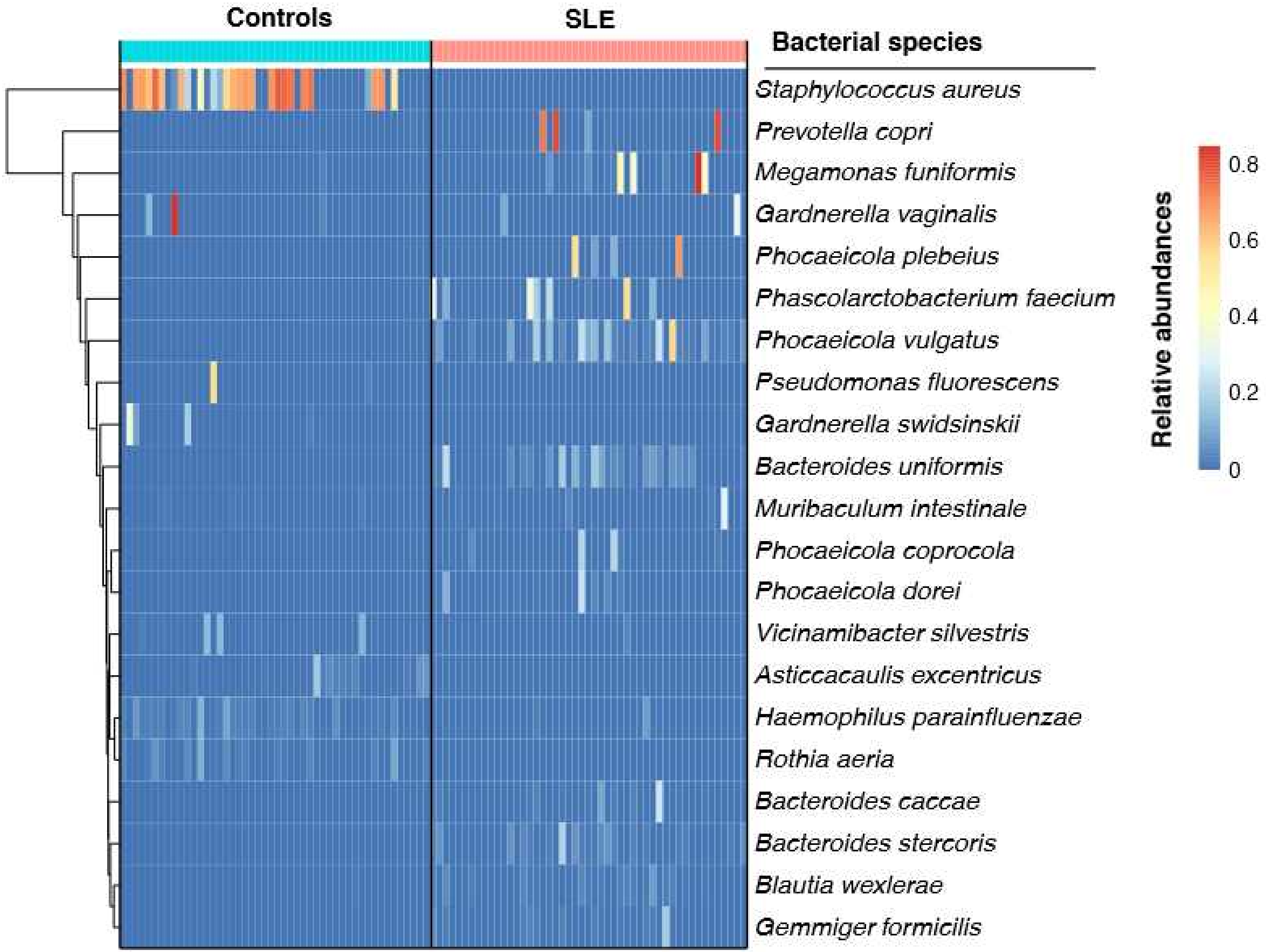
Controls
SLE
Bacterial species
Staphylococcus aureus
Prevotella copri
Megamonas funiformis
Gardnerella vaginalis
Phocaeicola plebeius
Phascolarctobacterium faecium
Phocaeicola vulgatus
Pseudomonas fluorescens
Gardnerella swidsinskii
Bacteroides uniformis
Muribaculum intestinale
Phocaeicola coprocola
Phocaeicola dorei
Vicinamibacter silvestris
Asticcacaulis excentricus
Haemophilus parainfluenzae
Rothia aeria
Bacteroides caccae
Bacteroides stercoris
Blautia wexlerae
Gemmiger formicilis
Relative abundances
0.8
0.6
0.4
0.2
0

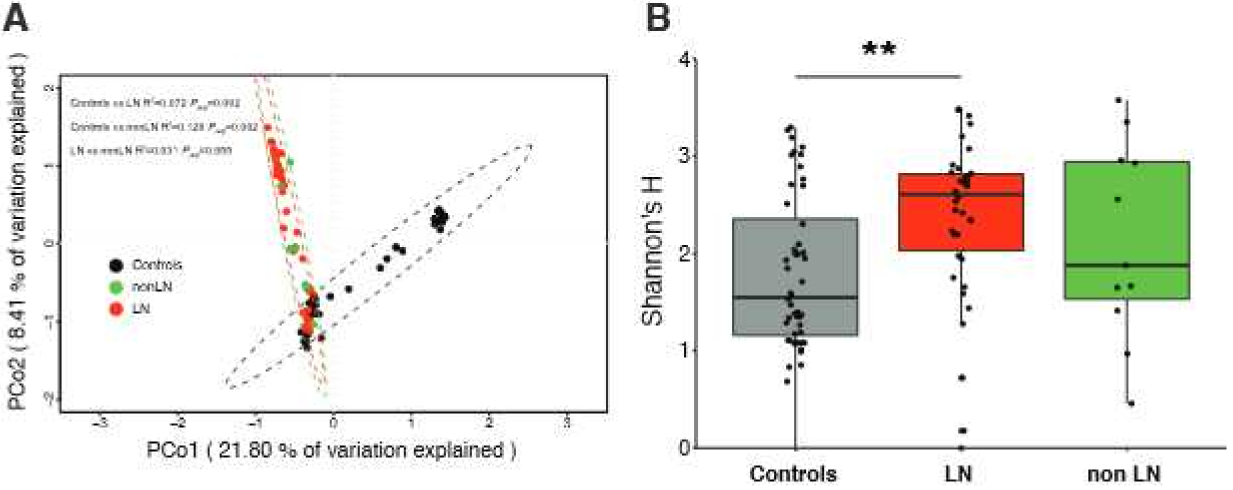

A
Controls
nonLN
LN
PCo1 (21.80 % of variation explained)
PCo2 (8.41 % of variation explained)
B
**
Shannon's H
Controls
LN
non LN

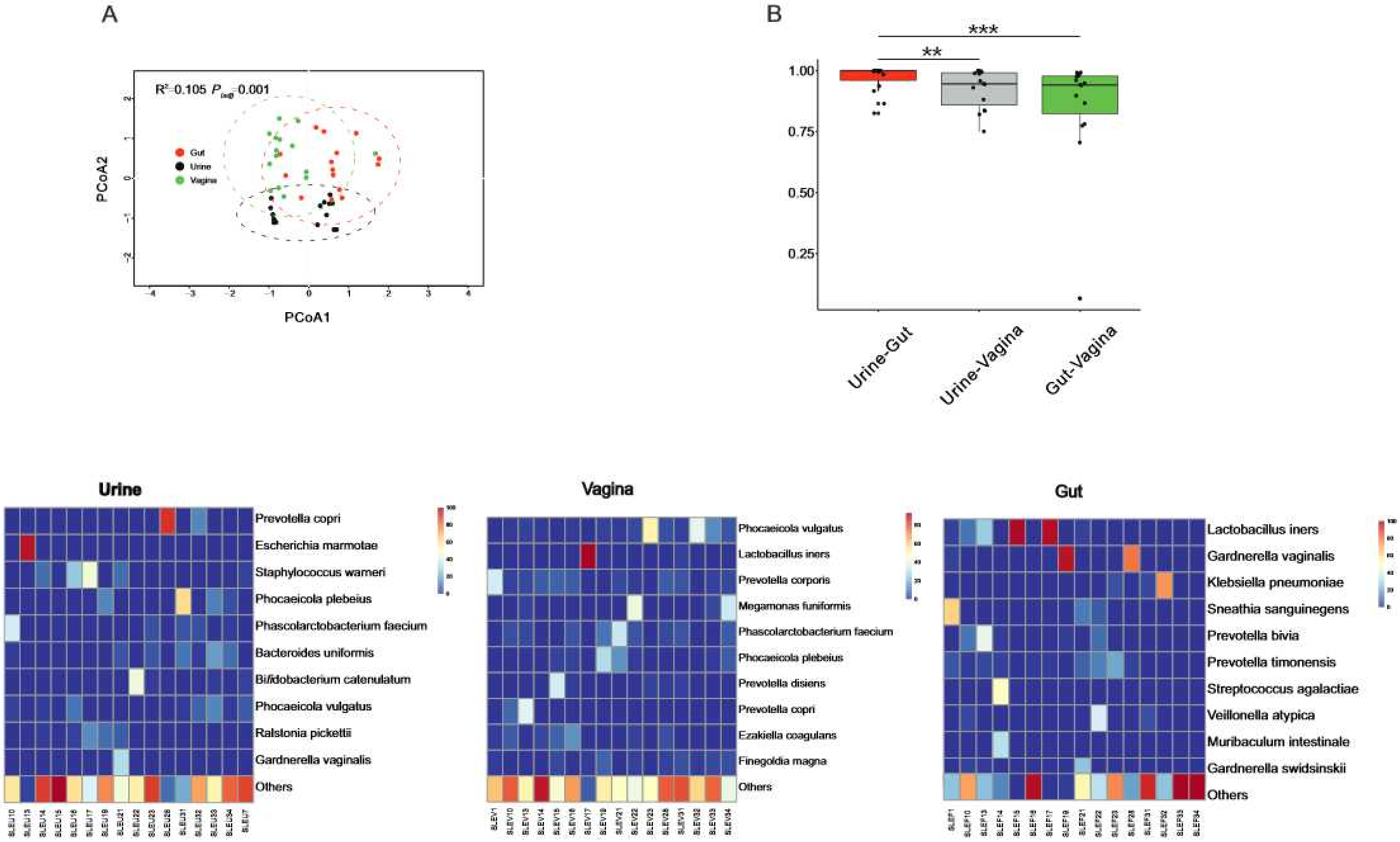
A
R²=0.105 P(adj)=0.001
Gut
Urine
Vagina
PCoA2
PCoA1
B
***
**
1.00
0.75
0.50
0.25
Urine-Gut
Urine-Vagina
Gut-Vagina
Urine
Prevotella copri
Escherichia marmotae
Staphylococcus warneri
Phocaeicola plebeius
Phascolarctobacterium faecium
Bacteroides uniformis
Bifidobacterium catenulatum
Phocaeicola vulgatus
Ralstonia pickettii
Gardnerella vaginalis
Others
Vagina
Phocaeicola vulgatus
Lactobacillus iners
Prevotella corporis
Megamonas funiformis
Phascolarctobacterium faecium
Phocaeicola plebeius
Prevotella disiens
Prevotella copri
Ezakiella coagulans
Finegoldia magna
Others
Gut
Lactobacillus iners
Gardnerella vaginalis
Klebsiella pneumoniae
Sneathia sanguinegens
Prevotella bivia
Prevotella timonensis
Streptococcus agalactiae
Veillonella atypica
Muribaculum intestinale
Gardnerella swidsinskii
Others

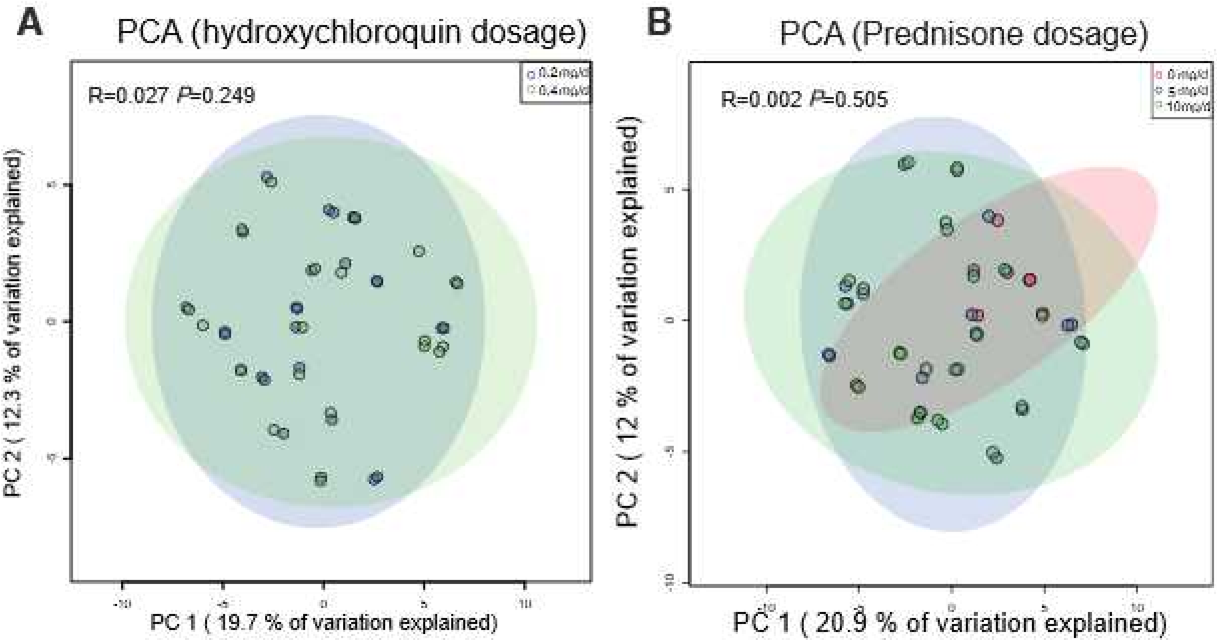
A
PCA (hydroxychloroquin dosage)
R=0.027 P=0.249
PC 2 (12.3 % of variation explained)
PC 1 (19.7 % of variation explained)
B
PCA (Prednisone dosage)
R=0.002 P=0.505
PC 2 (12 % of variation explained)
PC 1 (20.9 % of variation explained)

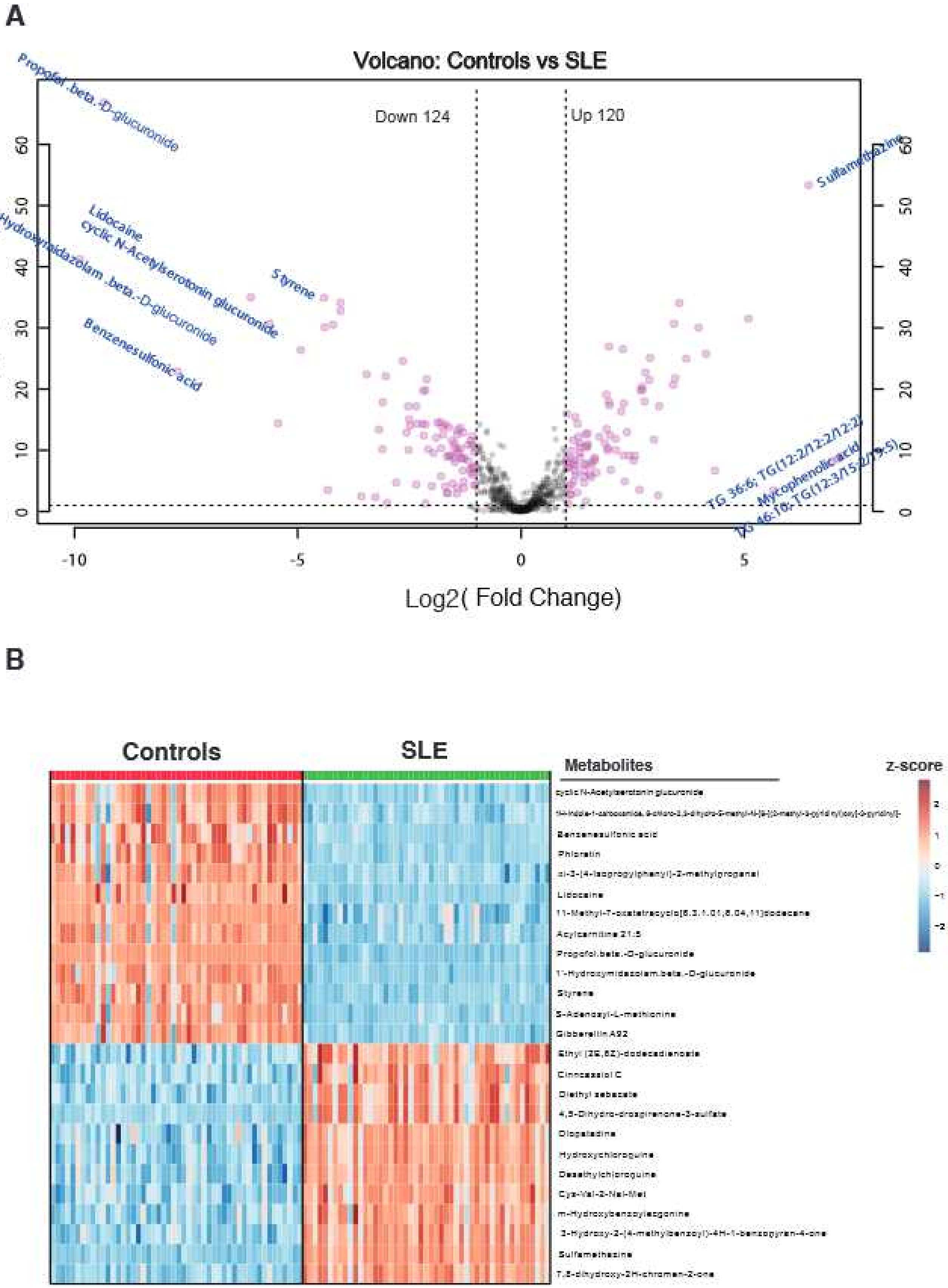
A
Volcano: Controls vs SLE
Down 124
Up 120
Propofol .beta.-D-glucuronide
Lidocaine
cyclic N-Acetylserotonin glucuronide
1'-Hydroxymidazolam .beta.-D-glucuronide
Styrene
Benzenesulfonic acid
Sulfamethazine
TG 36:6; TG(12:2/12:2/12:2)
Mycophenolic acid
TG 46:10; TG(12:3/15:2/19:5)
-log10(p-value)
Log2(Fold Change)
-10
-5
0
5
0
10
20
30
40
50
60
B
Controls
SLE
Metabolites
z-score
cyclic N-Acetylserotonin glucuronide
Benzenesulfonic acid
Phloretin
Lidocaine
11-Methyl-7-oxatetracyclo[6.3.1.01,6.04,11]dodecane
Acylcarnitine 21:5
Propofol.beta.-D-glucuronide
1'-Hydroxymidazolam.beta.-D-glucuronide
Styrene
S-Adenosyl-L-methionine
Gibberellin A92
Ethyl (2E,6Z)-dodecadienoate
Cinncassiol C
Diethyl sebacate
4,5-Dihydro-drospirenone-3-sulfate
Olopatadine
Hydroxychloroquine
Desethylchloroquine
Cys-Val-2-Nal-Met
m-Hydroxybenzoylecgonine
3-Hydroxy-2-(4-methylbenzoyl)-4H-1-benzopyran-4-one
Sulfamethazine
7,8-dihydroxy-2H-chromen-2-one
2
1
0
-1
-2

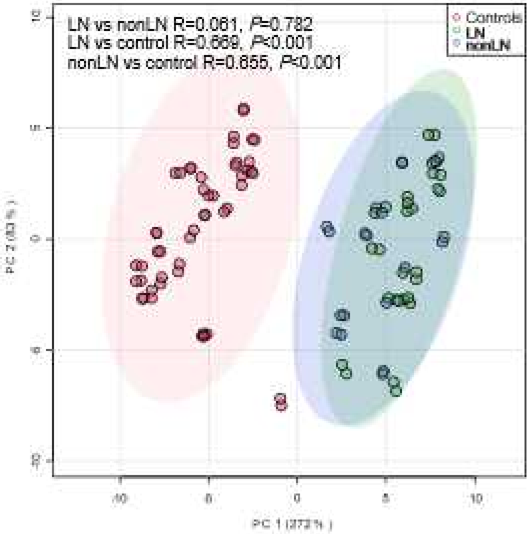
LN vs nonLN R=0.061, P=0.782
LN vs control R=0.669, P<0.001
nonLN vs control R=0.855, P<0.001
Controls
LN
nonLN

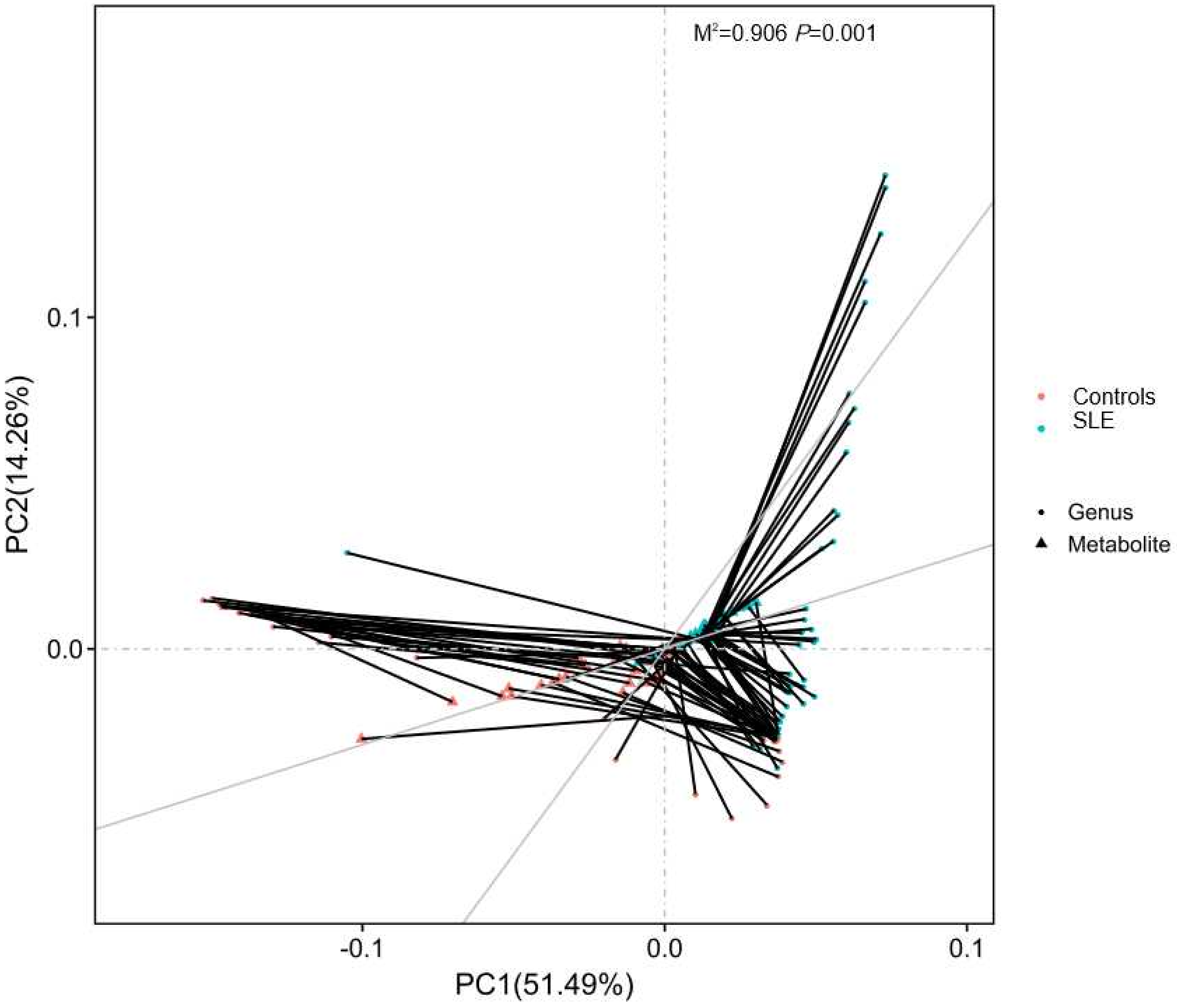
M²=0.906 P=0.001
PC2(14.26%)
PC1(51.49%)
0.1
0.0
-0.1
0.0
0.1
Controls
SLE
Genus
Metabolite

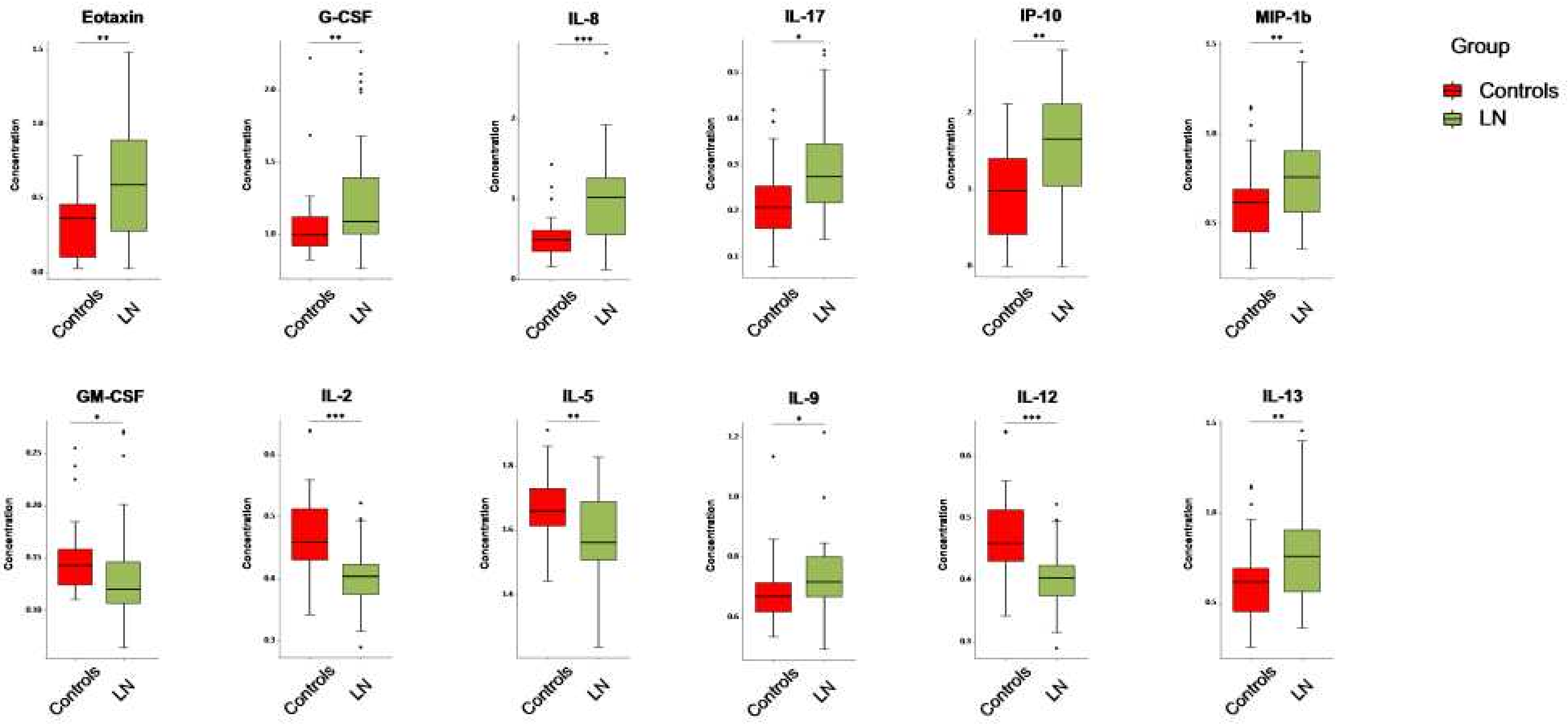
Eotaxin
G-CSF
IL-8
IL-17
IP-10
MIP-1b
GM-CSF
IL-2
IL-5
IL-9
IL-12
IL-13
Concentration
Controls
LN
Group
Controls
LN

Legends of supplementary tables

Tab. S1 original output for dada2+BLCA

Tab. S2 Detectable bacterial phylum and its relative abundance

Tab. S3 Detectable bacterial genus and its relative abundance

Tab. S4 Detectable bacterial species and its relative abundance

Tab. S5 Annotable metabolites.

Untargeted urinary metabolite profile was performed on liquid chromatography tandem mass spectrometry.

Tab. S6 SLE patient's characteristics

Abbreviations: "-" and "+" represent negative and positive, respectively; ACA, anti-centromere antibodies; AHAs, Antihistone antibodies; AMA-M2, Anti-mitochondrial M2 antibody; ANAs, antinuclear antibodies; Anti-dsDNA, anti-double stranded DNA; Anti-NCS, anti-nucleosome antibodies; Anti-nRNP, anti-nRNP antibodies; Anti-PCNA, antibodies to the proliferating cell nuclear antigen; Anti-PM/Scl, anti-PM/Scl antibodies; Anti-Ro/SSA, anti-Ro/SSA antibodies; Anti-Ro52, anti-Ro52 antibodies; Anti-Scl-70, anti-Scl 70 antibodies; Anti-Sm, anti-Smith antibodies; Anti-SSB, anti-SSB antibodies; ASO, anti-streptolysin O; ESR, erythrocyte sedimentation rate; HCQ, hydroxychloroquin; IgG, immunoglobulin G; Meth, methylprednisolone; MTX, methotrexate; Pred, prednisone; RF, rheumatoid factor

Tab. S7 Comparison of nutrient intake between controls and SLE.

Student's t test on normalized continuous variables and Wilcoxon rank-sum test was used on un-normalized continuous variables.

Tab. S8 Bacterial taxonomy affected by food intake.

MaAsLin (Microbiome Multivariable Associations with Linear Models) was used to adjust confounding factors, food intake, on bacteria showing significant difference at their abundance using Wilcoxon rank-sum test (Galaxy Version 1.0.1). P value was adjusted using Benjamin Hochberg false discovery rate (FDR).

Tab. S9 Demographics of LN and non-LN patients

a n, number of subjects;

b Mean ± SD or n (%);

c Pearson Chi-square or Fisher's exact test was used with categorical variables; Wilcoxon rank-sum test was used on un-normalized continuous variables.

Abbreviations: LN, lupus nephritis; NA, not applicable; SLEDAI, systemic lupus erythematosus disease activity index

Tab. S10. ASV features and its corresponding bacterial species in feces, urine and vagina in SLE patients

Tab. S11 Metabolite comparison between using Wilcoxon rank-sum rank test (Controls and LN)

Wilcoxon rank-sum test was used on the metabolites. P value was adjusted using Benjamin Hochberg false discovery rate (FDR).

Tab. S12 Metabolites showing Fold change>2 or <0.5 (Controls vs SLE)

For paired fold change analysis, the algorithm first counts the total number of pairs with fold changes that are consistently above/below the specified FC threshold >2 or <0.5 for each variable.

Tab. S13 Metabolites with VIP >1 (Controls vs SLE)

VIP was calculated using PLS-DA analysis

Abbreviation: VIP, Variable Importance in Projection

Tab. S14 The effects of nutrient intake on metabolites showing significant different between controls and SLE

Binary logistic regression model was used.

Abbreviations: B, coefficient value; SE, standard error; df, degrees of freedom; 95% CI, 95% confidence interval

Tab. S15 Effects of hydroxychloroquine intake on metabolites

Wilcoxon rank-sum test was used on the metabolites. P value was adjusted using Benjamin Hochberg false discovery rate (FDR).

Tab. S16 Effects of prednisone intake on metabolites

Wilcoxon rank-sum test was used on the metabolites. P value was adjusted using Benjamin Hochberg false discovery rate (FDR).

Tab. S17 The effects of hydroxychloroquine intake on urinary hydroxychloroquine and Desethylchloroquine

Binary logistic regression model was used.

Abbreviations: B, coefficient value; SE, standard error; df, degrees of freedom; 95%CI, 95% confidence interval

Tab. S18 Metabolite comparison between controls and LN

Wilcoxon rank-sum test was used on the metabolites. P value was adjusted using Benjamin Hochberg false discovery rate (FDR).

Abbreviation: LN, lupus nephritis

Tab. S19 Metabolites comparison between controls and LN showing Fold change>2 or <0.5

For paired fold change analysis, the algorithm first counts the total number of pairs with fold changes that are consistently above/below the specified FC threshold >2 or <0.5 for each variable.

Tab. S20 Metabolites with VIP >1 (Controls vs LN)

VIP was calculated using PLS-DA analysis.

Abbreviation: VIP, Variable Importance in Projection

Tab. S21 The area under the ROC curve (AUC) of urinary metabolite based on lupus nephritis classification

The metabolites displayed in the table are based on area under ROC curve (AUCROC). Only the metabolites with AUC ≥ 0.85 are displayed. The 95% confidence interval is calculated using 500 bootstrappings.

Tab. S22 Metabolite comparison between controls and nonLN

Wilcoxon rank-sum test was used on the metabolites. P value was adjusted using Benjamin Hochberg false discovery rate (FDR). Abbreviation: nonLN, non-lupus nephritis.

Abbreviation: nonLN, non-lupus nephritis

Tab. S23 Metabolites showing Fold change>2 or <0.5 (controls vs nonLN)

For paired fold change analysis, the algorithm first counts the total number of pairs with fold changes that are consistently above/below the specified FC threshold >2 or <0.5 for each variable.

Abbreviation: nonLN, non-lupus nephritis

Tab. S24 Metabolites with VIP >1 (Controls vs nonLN)

VIP was calculated using PLS-DA analysis.

Abbreviation: LN, lupus nephritis; VIP, Variable Importance in Projection.

Tab. S24 Metabolites with VIP >1 (Controls vs nonLN)

VIP was calculated using PLS-DA analysis.

Abbreviation: LN, lupus nephritis; VIP, Variable Importance in Projection.

Tab. S25 The area under the ROC curve (AUC) of urinary metabolite based on non-lupus nephritis classification

The metabolites displayed in the table are based on area under ROC curve (AUCROC). Only the metabolites with AUC ≥ 0.85 are displayed. The 95% confidence interval is calculated using 500 bootstrappings.

Tab. S26 Correlation between bacterial genus and metabolites that showed significant difference between controls and SLE

Spearman correlation evaluated the linear relationship between bacterial genus and metabolite.

Tab. S27 Correlation between bacterial genus and cytokines that showed significant difference between controls and SLE

Spearman correlation evaluated the linear relationship between bacterial genus and cytokine.

Tab. S28 Correlation between bacterial genus and disease profiles in SLE patients

Spearman correlation evaluated the linear relationship between bacterial genus and disease profile.

[illegible]

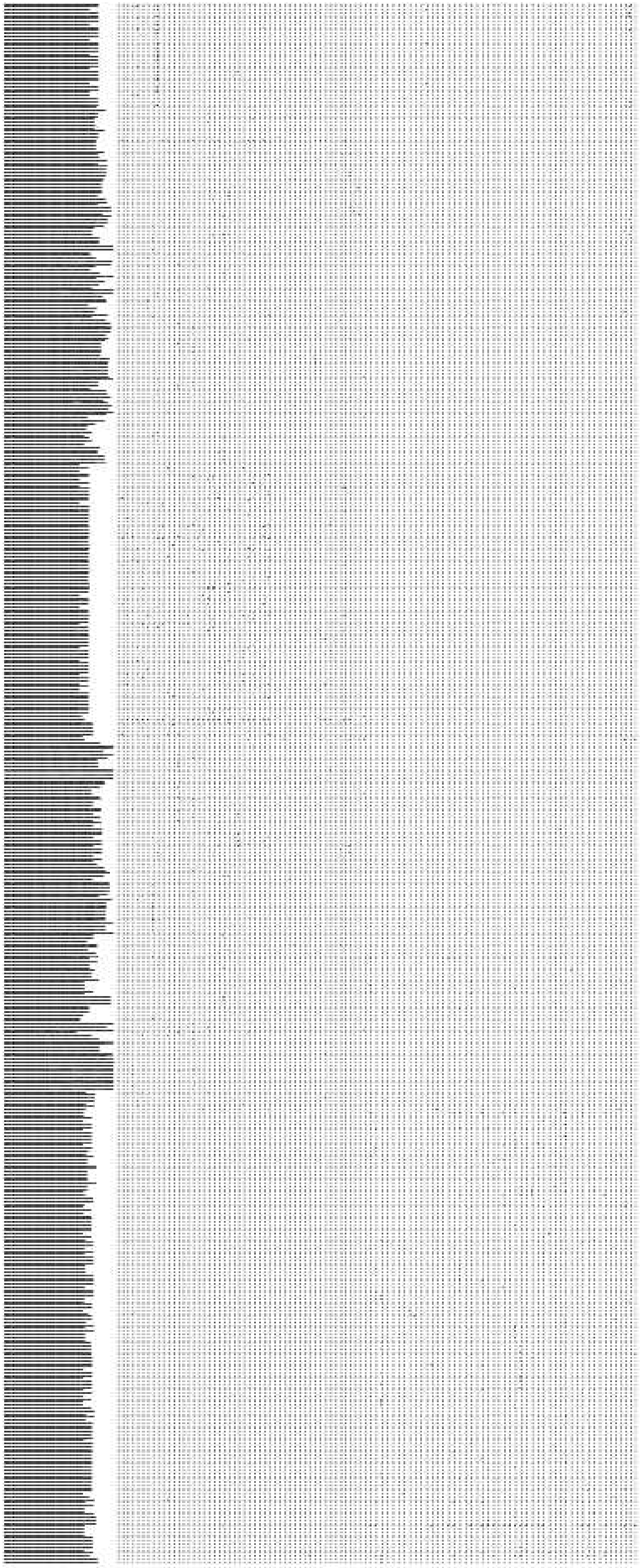

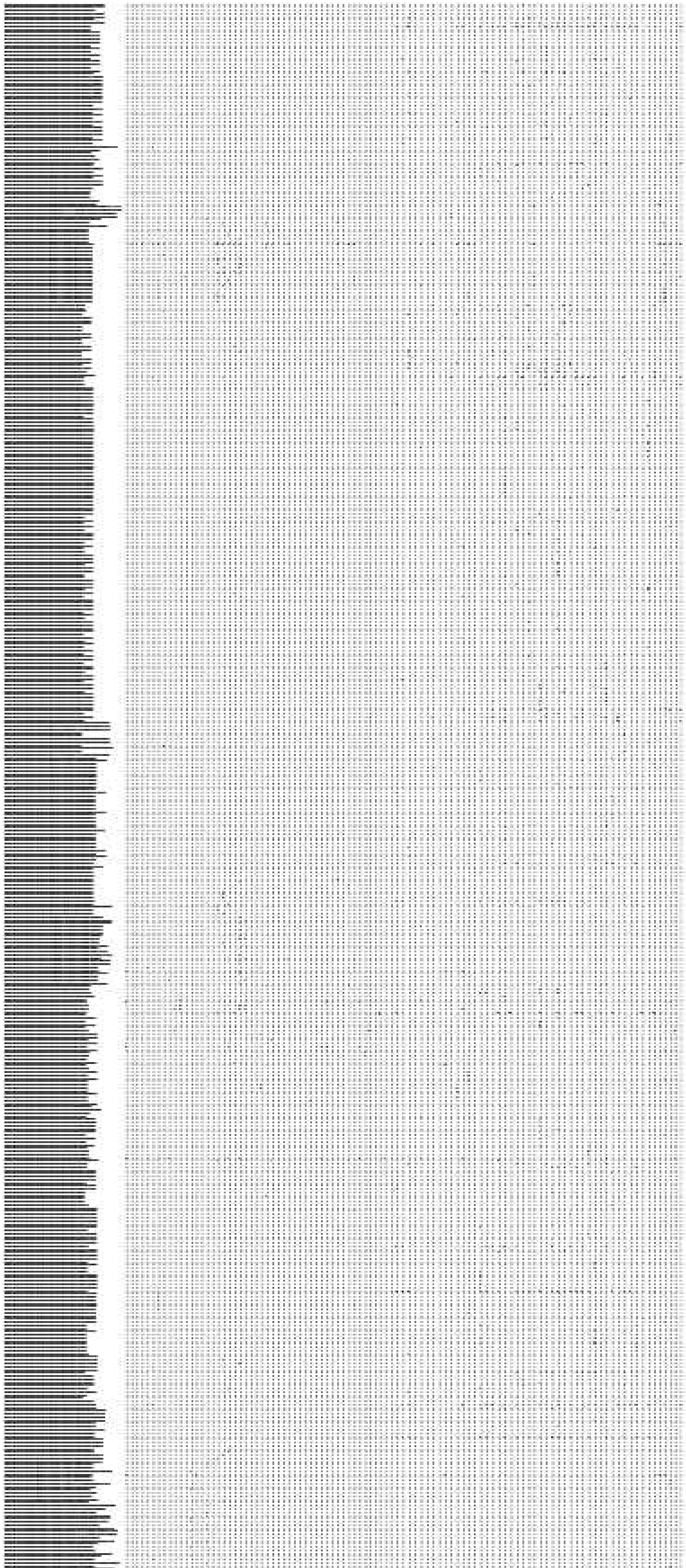

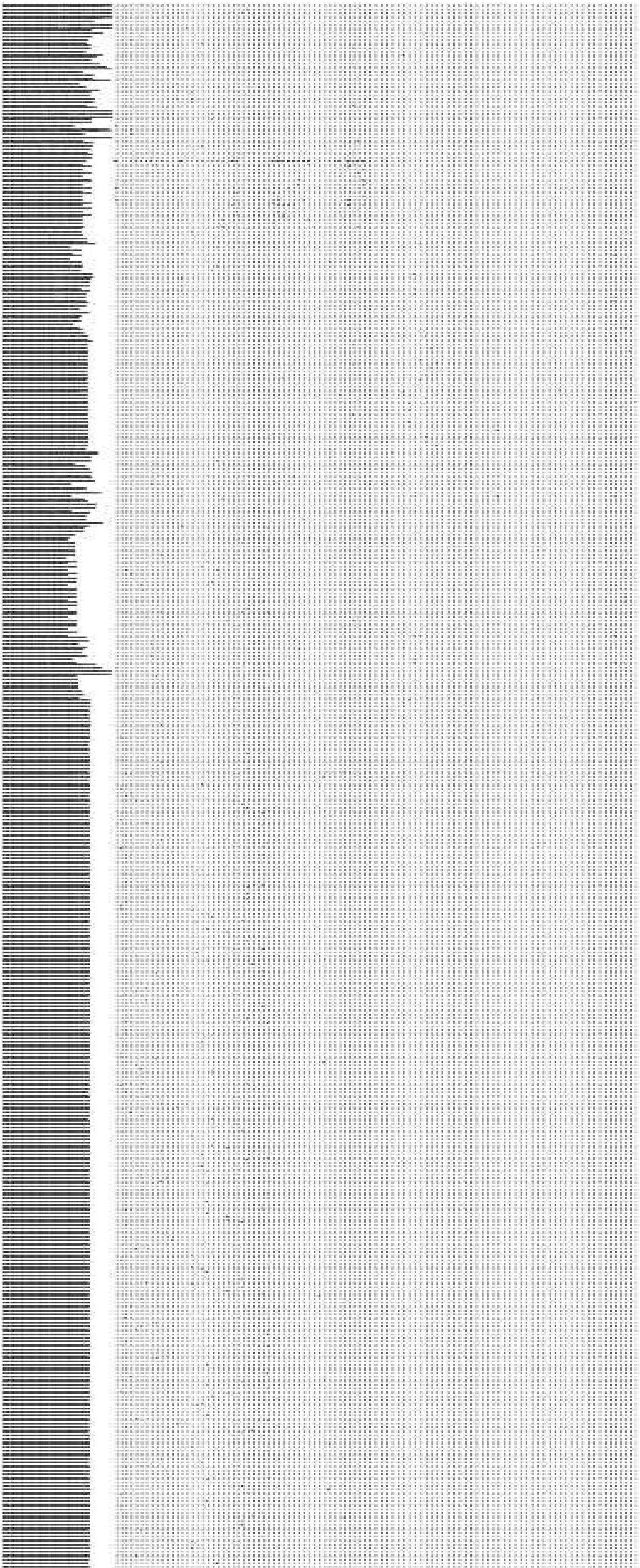

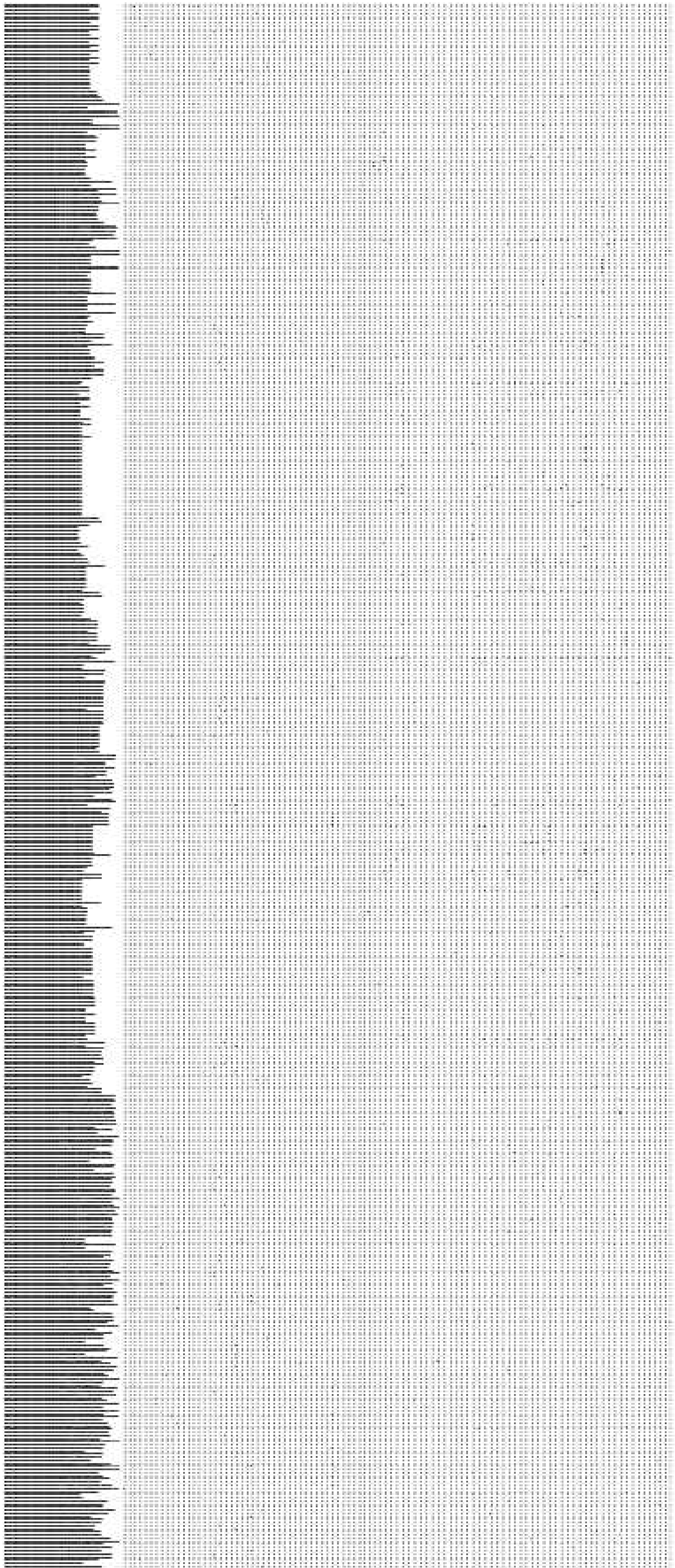

[illegible]

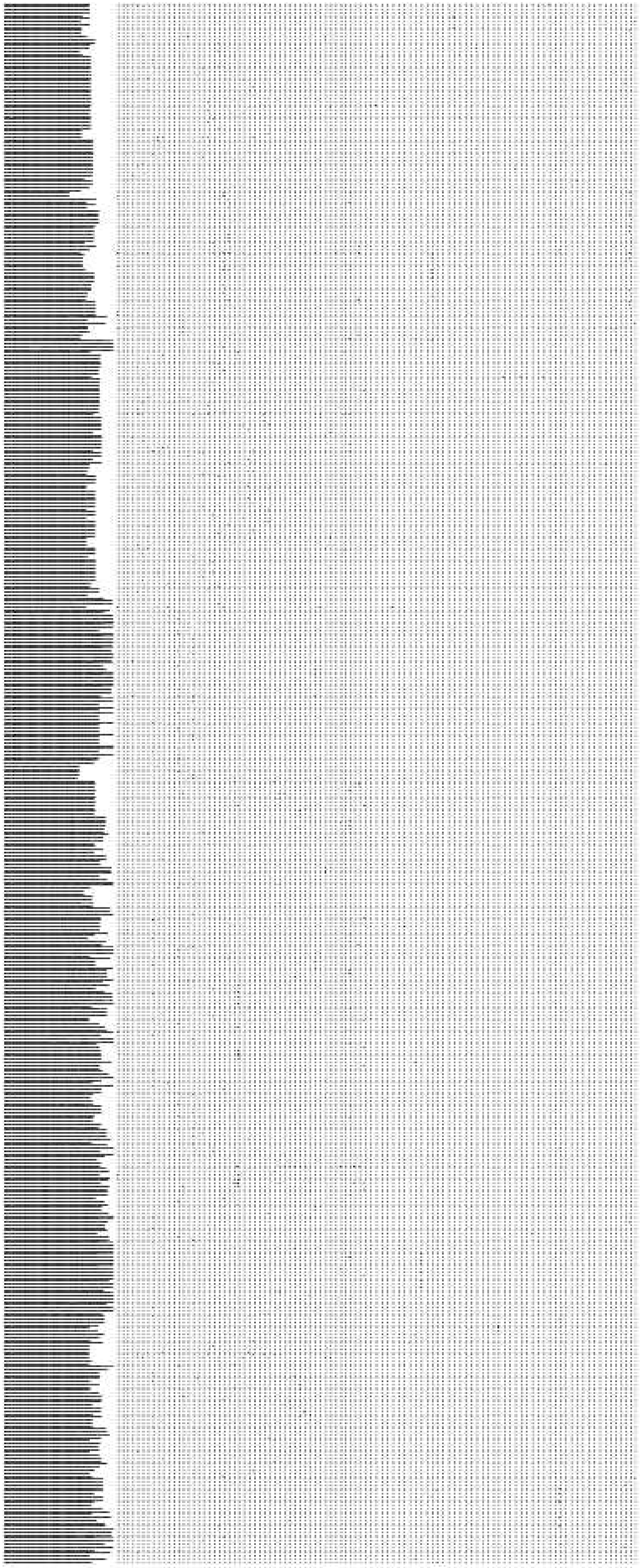

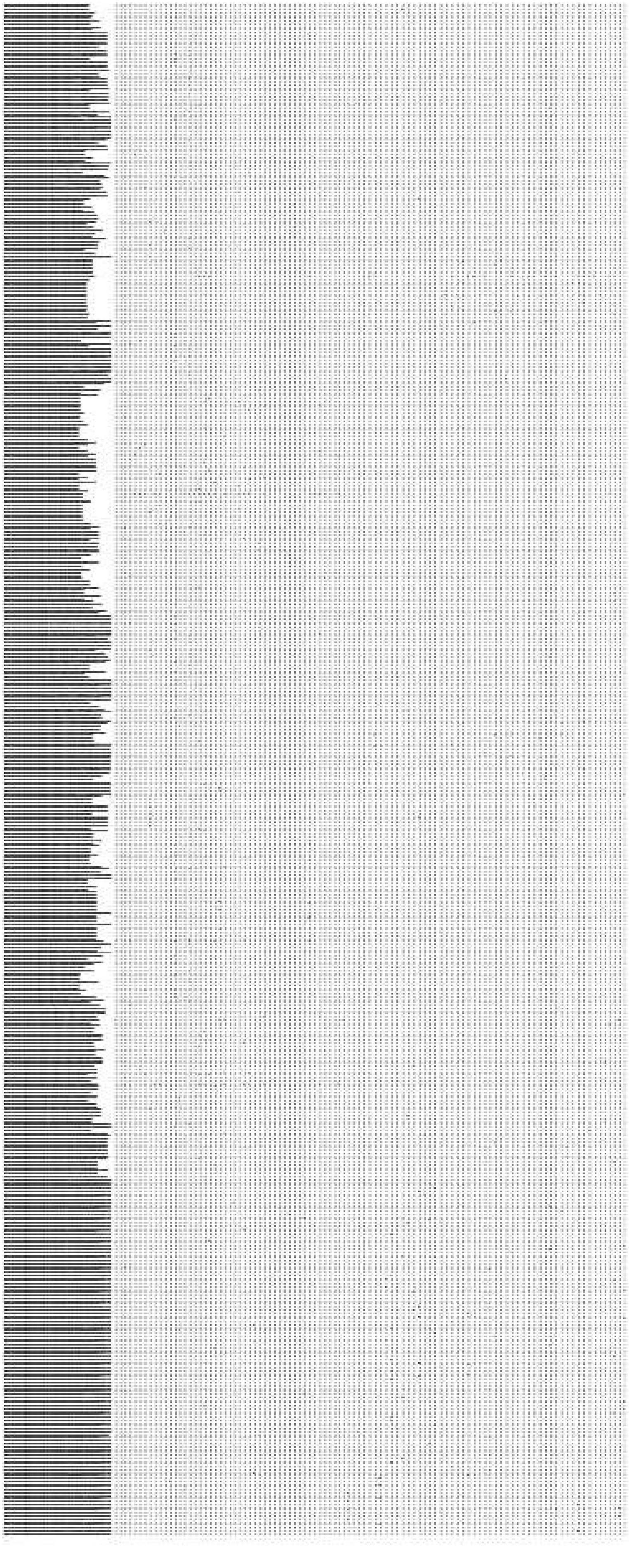

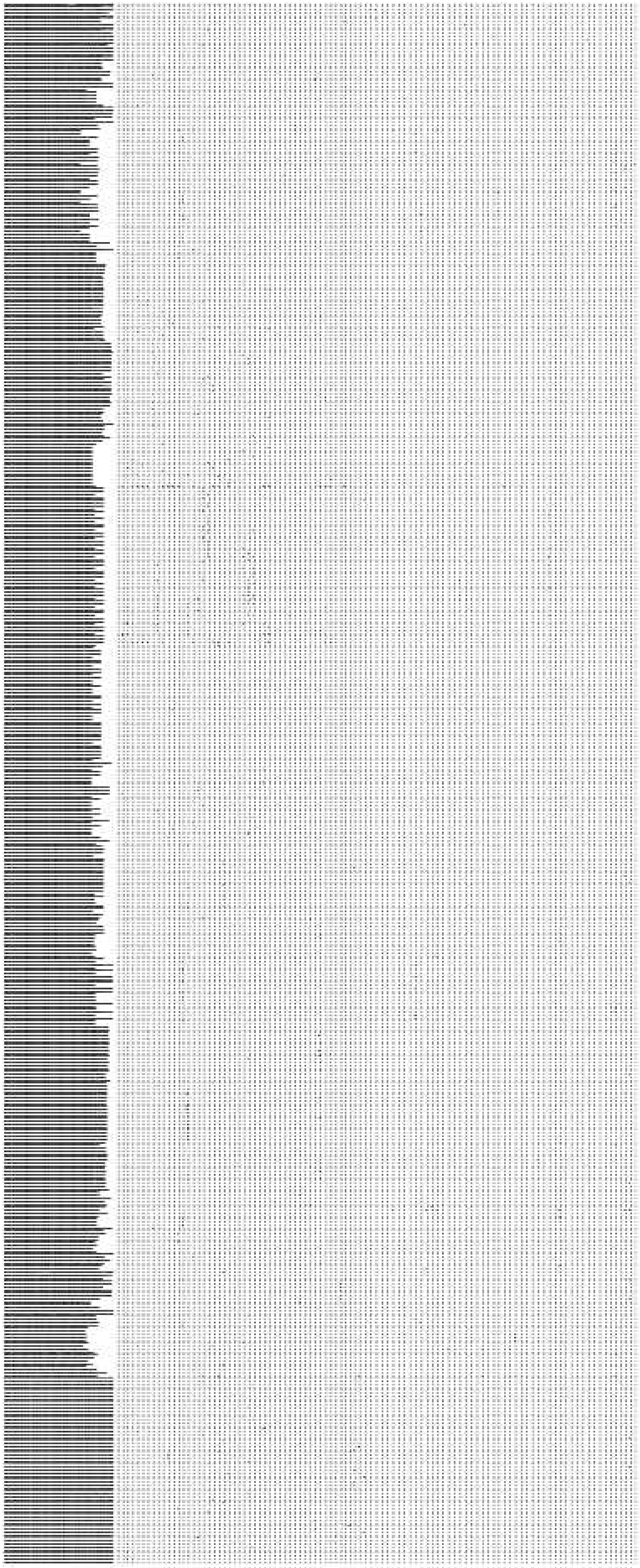

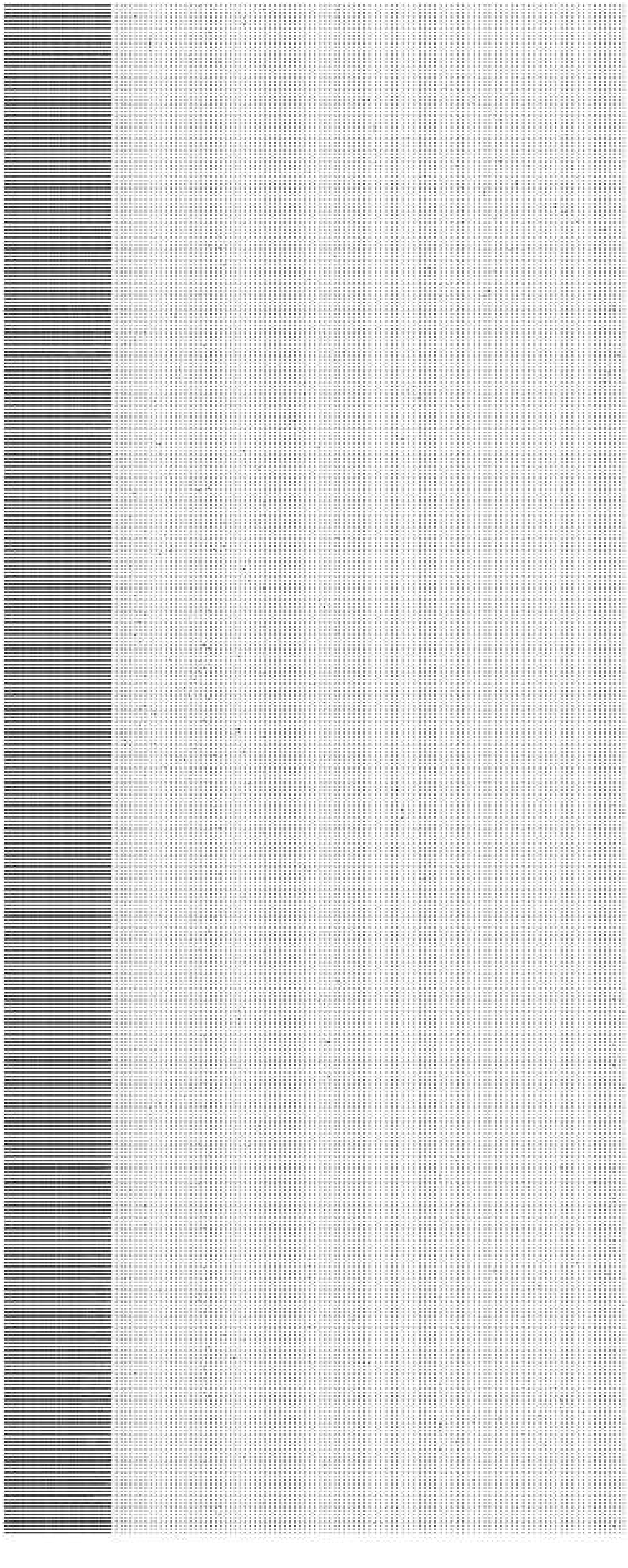

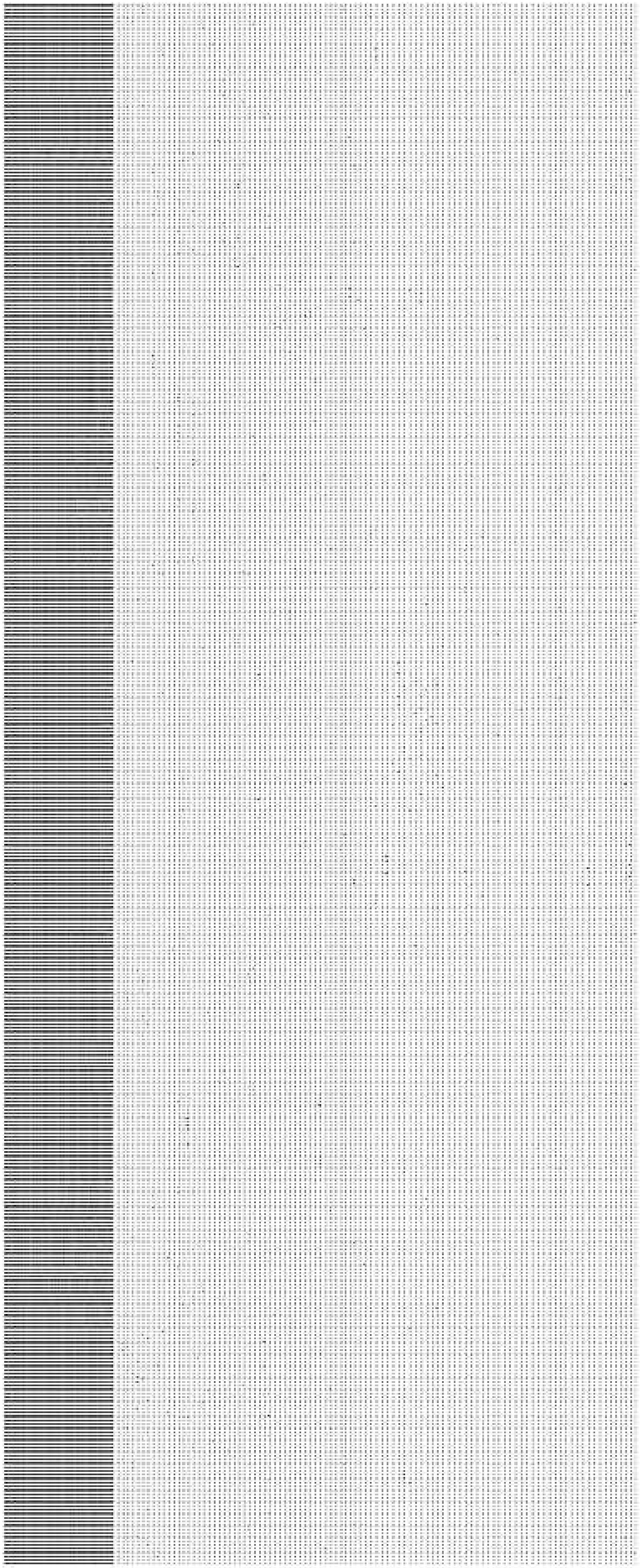

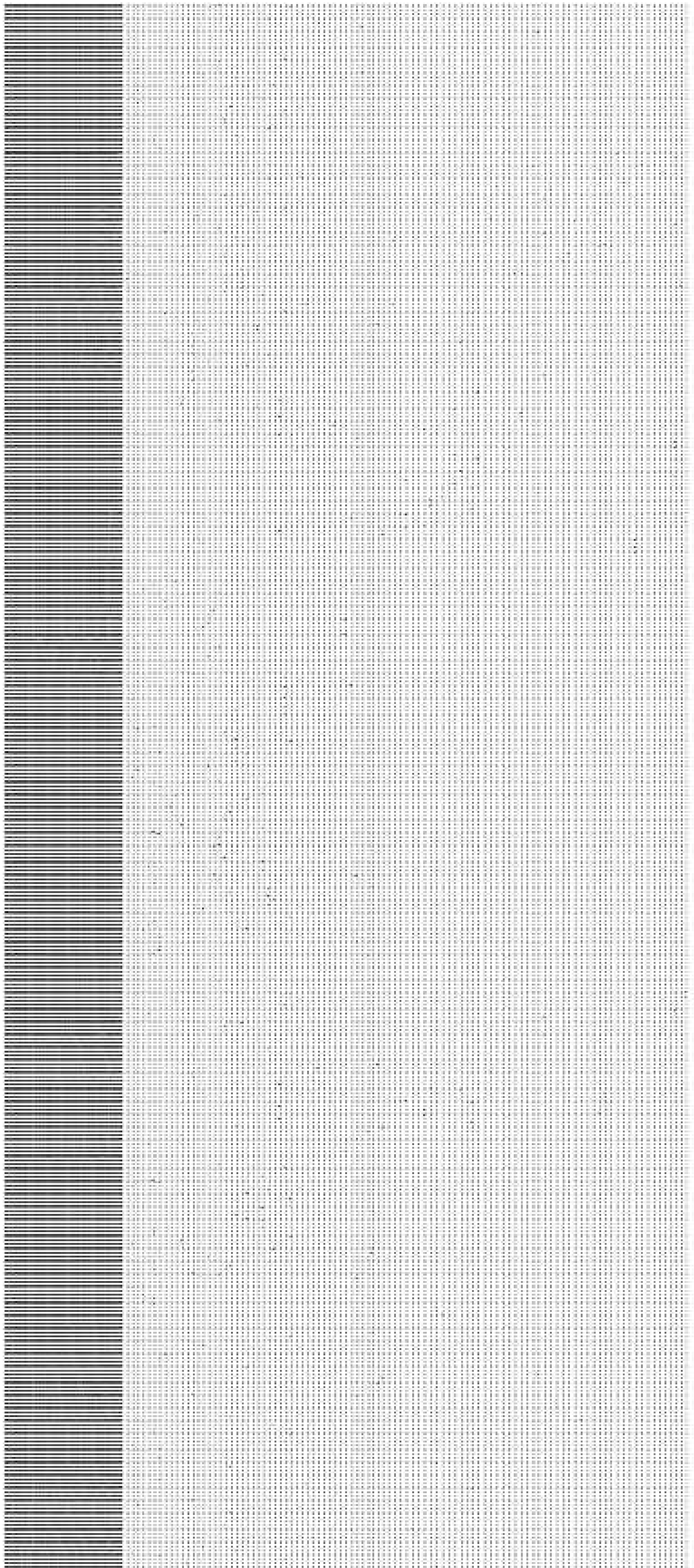

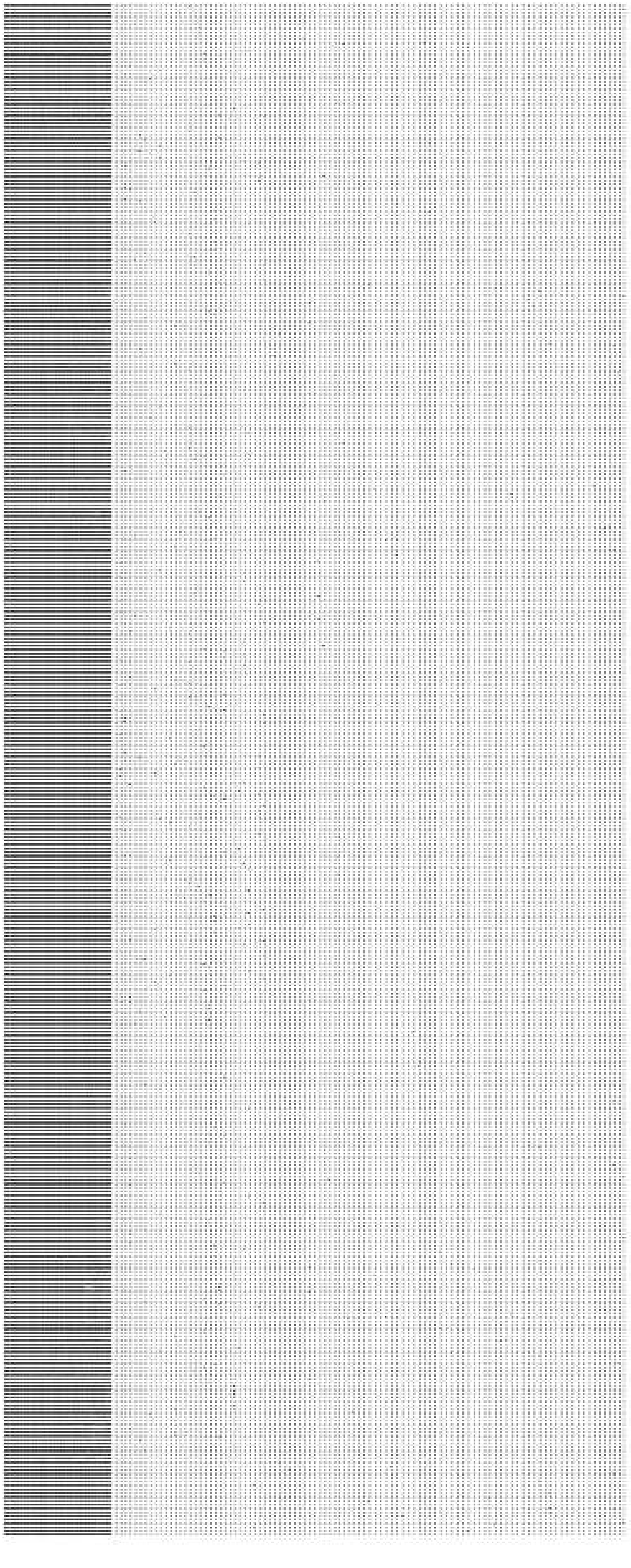

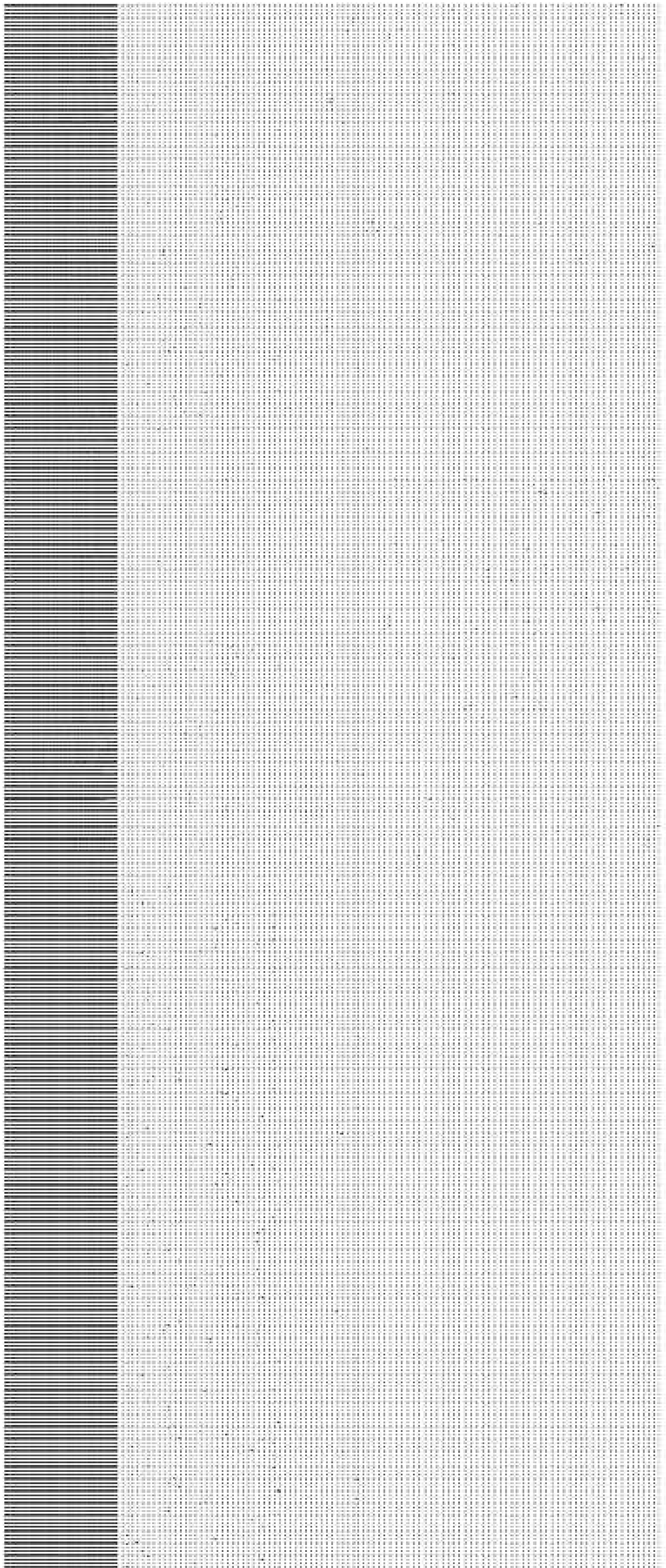

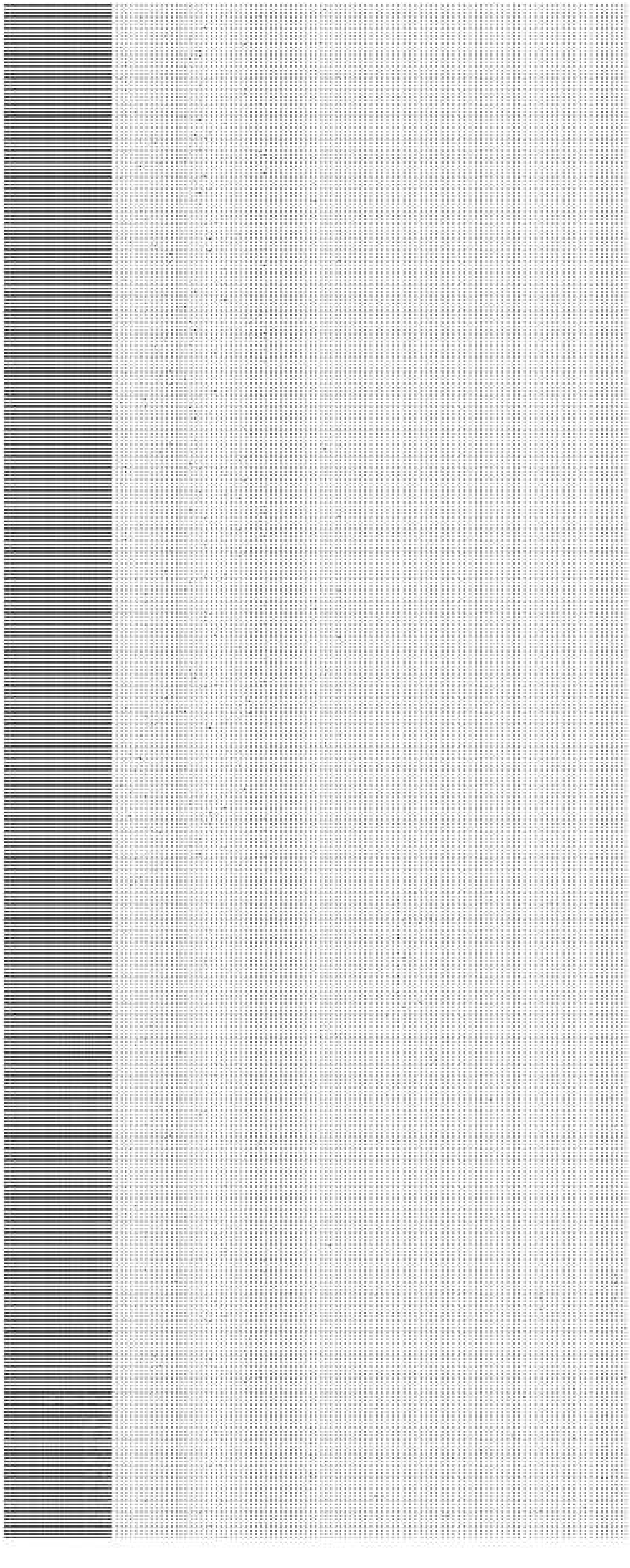

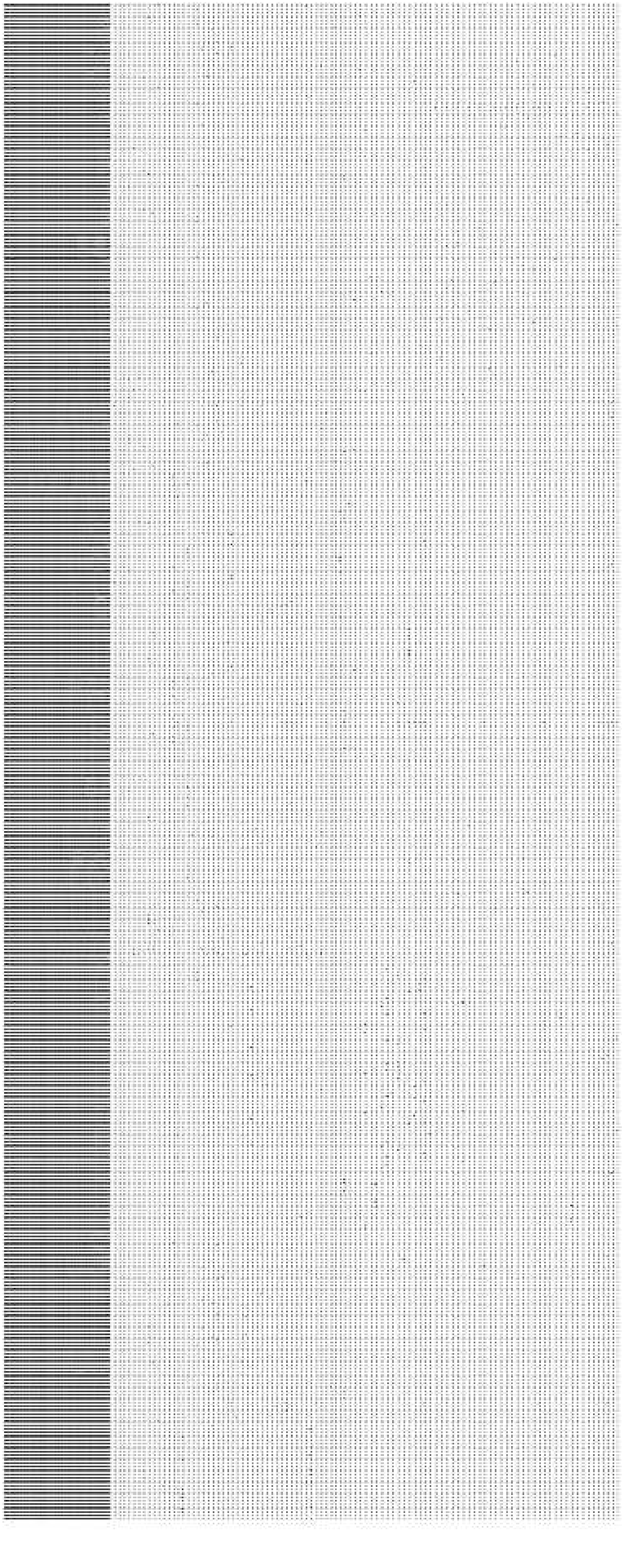

Tab. S3 Detectable bacterial phylum and its relative abundance

ID	Acidobacteria	Actinobacteria	Bacteroidetes	Chloroflexi	Cyanobacteria	Deferribacteres	Firmicutes	Fusobacteria	Gemmatimonadetes	Lentisphaerae	Nitrospirae	Proteobacteria	Spirochaetes	Synergistetes	Tenericutes	Thermotogae	Unclassified	Verrucomicrobia
BC10	0.00	0.20	0.10	0.00	0.01	0.00	0.28	0.06	0.00	0.00	0.00	0.08	0.00	0.00	0.00	0.00	0.00	0.00
BC11	0.00	0.04	0.00	0.00	0.00	0.00	0.47	0.00	0.00	0.00	0.00	0.05	0.00	0.00	0.00	0.00	0.00	0.00
BC1	0.00	0.00	0.01	0.00	0.00	0.00	0.70	0.00	0.00	0.00	0.00	0.00	0.00	0.00	0.00	0.00	0.00	0.00
BC12	0.02	0.08	0.01	0.00	0.01	0.00	0.76	0.01	0.01	0.00	0.00	0.03	0.00	0.00	0.00	0.00	0.00	0.00
BC13	0.01	0.02	0.01	0.00	0.00	0.00	0.48	0.01	0.00	0.00	0.00	0.07	0.00	0.00	0.00	0.00	0.00	0.00
BC14	0.00	0.13	0.01	0.00	0.00	0.00	0.83	0.00	0.00	0.00	0.00	0.06	0.00	0.00	0.00	0.00	0.00	0.00
BC15	0.02	0.08	0.04	0.00	0.00	0.00	0.78	0.01	0.00	0.00	0.00	0.08	0.00	0.00	0.00	0.00	0.00	0.00
BC16	0.04	0.31	0.08	0.00	0.00	0.00	0.22	0.02	0.00	0.00	0.00	0.33	0.00	0.00	0.01	0.00	0.00	0.00
BC17	0.00	0.00	0.00	0.00	0.00	0.00	0.00	0.00	0.00	0.00	0.00	0.00	0.00	0.00	0.00	0.00	0.00	0.00
BC18	0.01	0.00	0.01	0.00	0.00	0.00	0.75	0.00	0.00	0.00	0.00	0.10	0.00	0.00	0.01	0.00	0.00	0.00
BC19	0.00	0.40	0.07	0.00	0.01	0.00	0.28	0.08	0.00	0.00	0.00	0.00	0.00	0.00	0.00	0.00	0.00	0.00
BC20	0.00	0.02	0.00	0.00	0.00	0.00	0.82	0.00	0.00	0.00	0.00	0.02	0.00	0.00	0.00	0.00	0.00	0.00
BC21	0.28	0.10	0.02	0.01	0.00	0.00	0.04	0.00	0.02	0.00	0.01	0.10	0.00	0.00	0.00	0.00	0.27	0.00
BC2	0.00	0.00	0.00	0.00	0.00	0.00	0.80	0.00	0.00	0.00	0.00	0.48	0.00	0.00	0.00	0.00	0.00	0.00
BC22	0.00	0.06	0.00	0.00	0.00	0.00	0.28	0.00	0.00	0.00	0.00	0.65	0.00	0.00	0.00	0.00	0.00	0.00
BC23	0.01	0.10	0.01	0.01	0.00	0.00	0.14	0.00	0.00	0.00	0.00	0.17	0.00	0.00	0.00	0.00	0.53	0.00
BC24	0.00	0.11	0.00	0.00	0.00	0.00	0.62	0.01	0.00	0.00	0.00	0.12	0.00	0.00	0.00	0.00	0.00	0.00
BC25	0.00	0.00	0.04	0.00	0.00	0.00	0.79	0.01	0.00	0.00	0.00	0.08	0.00	0.00	0.00	0.00	0.00	0.00
BC26	0.01	0.07	0.02	0.00	0.00	0.00	0.81	0.01	0.00	0.00	0.00	0.06	0.00	0.00	0.00	0.00	0.00	0.00
BC27	0.00	0.11	0.02	0.00	0.00	0.00	0.70	0.02	0.00	0.00	0.00	0.08	0.00	0.00	0.00	0.00	0.00	0.00
BC28	0.01	0.10	0.02	0.00	0.00	0.00	0.78	0.00	0.00	0.00	0.00	0.07	0.00	0.00	0.00	0.00	0.00	0.00
BC29	0.00	0.10	0.18	0.00	0.00	0.00	0.08	0.01	0.00	0.00	0.00	0.10	0.00	0.00	0.00	0.00	0.00	0.00
BC31	0.00	0.11	0.02	0.00	0.00	0.00	0.21	0.01	0.00	0.00	0.00	0.04	0.00	0.00	0.00	0.00	0.00	0.00
BC3	0.00	0.04	0.00	0.00	0.00	0.00	0.12	0.00	0.00	0.00	0.00	0.80	0.00	0.00	0.00	0.00	0.00	0.00
BC32	0.00	0.07	0.02	0.00	0.00	0.00	0.84	0.01	0.00	0.00	0.00	0.04	0.00	0.00	0.00	0.00	0.00	0.00
BC33	0.01	0.08	0.01	0.00	0.00	0.00	0.82	0.01	0.00	0.00	0.00	0.07	0.00	0.00	0.00	0.00	0.00	0.00
BC34	0.01	0.08	0.01	0.00	0.00	0.00	0.81	0.01	0.00	0.00	0.00	0.07	0.00	0.00	0.00	0.00	0.00	0.00
BC35	0.00	0.00	0.00	0.00	0.00	0.00	0.00	0.00	0.00	0.00	0.00	1.00	0.00	0.00	0.00	0.00	0.00	0.00
BC36	0.00	0.10	0.00	0.00	0.00	0.00	0.80	0.01	0.00	0.00	0.00	0.06	0.00	0.00	0.00	0.00	0.00	0.00
BC37	0.00	0.12	0.01	0.00	0.00	0.00	0.80	0.01	0.00	0.00	0.00	0.06	0.00	0.00	0.00	0.00	0.00	0.00
BC38	0.00	0.22	0.00	0.00	0.00	0.00	0.08	0.00	0.00	0.00	0.00	0.60	0.00	0.00	0.00	0.00	0.00	0.00
BC40	0.06	0.03	0.00	0.00	0.00	0.00	0.07	0.00	0.00	0.00	0.00	0.66	0.00	0.00	0.00	0.00	0.00	0.00
BC41	0.02	0.02	0.12	0.00	0.00	0.00	0.02	0.00	0.00	0.00	0.00	0.75	0.00	0.00	0.00	0.00	0.00	0.00
BC4	0.02	0.11	0.01	0.00	0.00	0.00	0.21	0.00	0.00	0.00	0.00	0.62	0.00	0.00	0.01	0.00	0.00	0.00
BC42	0.02	0.11	0.08	0.00	0.00	0.00	0.22	0.01	0.00	0.00	0.00	0.57	0.00	0.00	0.00	0.00	0.00	0.00
BC43	0.00	0.08	0.02	0.00	0.00	0.00	0.28	0.00	0.00	0.00	0.00	0.18	0.00	0.00	0.00	0.00	0.00	0.00
BC44	0.00	0.01	0.01	0.00	0.00	0.00	0.11	0.00	0.00	0.00	0.00	0.80	0.00	0.00	0.00	0.00	0.00	0.00
BC45	0.18	0.10	0.08	0.00	0.00	0.00	0.08	0.00	0.00	0.00	0.00	0.24	0.00	0.00	0.00	0.00	0.00	0.00
BC46	0.00	0.21	0.00	0.00	0.00	0.00	0.12	0.00	0.00	0.00	0.00	0.02	0.00	0.00	0.00	0.00	0.00	0.00
BC47	0.04	0.06	0.01	0.00	0.00	0.00	0.76	0.01	0.00	0.00	0.00	0.10	0.00	0.00	0.00	0.00	0.00	0.00
BC48	0.01	0.06	0.02	0.00	0.00	0.00	0.80	0.00	0.00	0.00	0.00	0.00	0.00	0.00	0.00	0.00	0.00	0.00
BC49	0.00	0.02	0.03	0.00	0.00	0.00	0.87	0.00	0.00	0.00	0.00	0.09	0.00	0.00	0.00	0.00	0.00	0.00
BC50	0.00	0.07	0.00	0.00	0.00	0.00	0.28	0.00	0.00	0.00	0.00	0.68	0.00	0.00	0.00	0.00	0.00	0.00
BC5	0.00	0.20	0.02	0.00	0.00	0.00	0.62	0.00	0.00	0.00	0.00	0.12	0.00	0.00	0.00	0.00	0.00	0.00
BC6	0.00	0.13	0.00	0.00	0.00	0.00	0.00	0.08	0.00	0.00	0.00	0.70	0.00	0.00	0.00	0.00	0.00	0.00
BC7	0.08	0.00	0.02	0.00	0.01	0.00	0.18	0.00	0.00	0.00	0.00	0.66	0.00	0.00	0.00	0.00	0.00	0.00
BC8	0.00	0.40	0.01	0.00	0.02	0.00	0.48	0.00	0.00	0.00	0.00	0.00	0.00	0.00	0.00	0.00	0.00	0.00
BC9	0.00	0.02	0.00	0.00	0.00	0.00	0.28	0.02	0.00	0.00	0.00	0.58	0.00	0.00	0.00	0.00	0.00	0.00
BLE10	0.00	0.04	0.28	0.00	0.00	0.00	0.48	0.00	0.00	0.00	0.00	0.02	0.00	0.00	0.00	0.00	0.00	0.00
BLE11	0.00	0.00	0.08	0.00	0.00	0.00	0.00	0.00	0.00	0.00	0.00	0.82	0.00	0.00	0.00	0.00	0.00	0.00
BLE12	0.00	0.01	0.84	0.00	0.00	0.00	0.42	0.00	0.00	0.00	0.00	0.00	0.00	0.00	0.00	0.00	0.00	0.00
BLE13	0.00	0.00	0.01	0.00	0.00	0.00	0.00	0.00	0.00	0.00	0.00	0.00	0.00	0.00	0.00	0.00	0.00	0.00
BLE14	0.00	0.00	0.11	0.00	0.00	0.00	0.27	0.18	0.00	0.00	0.00	0.47	0.00	0.00	0.00	0.00	0.00	0.00
BLE15	0.00	0.00	0.00	0.00	0.00	0.00	0.00	0.00	0.00	0.00	0.00	1.00	0.00	0.00	0.00	0.00	0.00	0.00
BLE16	0.00	0.01	0.18	0.00	0.00	0.00	0.71	0.00	0.00	0.00	0.00	0.12	0.00	0.00	0.00	0.00	0.00	0.00
BLE17	0.00	0.02	0.04	0.00	0.00	0.00	0.47	0.00	0.00	0.00	0.00	0.50	0.00	0.00	0.00	0.00	0.00	0.00
BLE18	0.00	0.00	0.10	0.00	0.00	0.00	0.42	0.00	0.00	0.00	0.00	0.08	0.01	0.00	0.00	0.00	0.00	0.00
BLE19	0.00	0.00	0.02	0.00	0.00	0.00	0.18	0.00	0.00	0.00	0.00	0.78	0.02	0.00	0.00	0.00	0.00	0.00
BLE20	0.00	0.11	0.00	0.00	0.00	0.00	0.10	0.00	0.00	0.00	0.00	0.67	0.00	0.00	0.00	0.00	0.00	0.00
BLE21	0.00	0.00	0.08	0.00	0.00	0.00	0.08	0.00	0.00	0.00	0.00	0.57	0.00	0.00	0.00	0.00	0.00	0.00
BLE2	0.00	0.00	0.12	0.00	0.00	0.00	0.20	0.00	0.00	0.00	0.00	0.67	0.00	0.00	0.00	0.00	0.00	0.00
BLE22	0.00	0.04	0.08	0.00	0.00	0.00	0.11	0.00	0.00	0.00	0.00	0.77	0.00	0.00	0.00	0.00	0.00	0.00
BLE23	0.00	0.01	0.20	0.00	0.00	0.00	0.47	0.00	0.00	0.00	0.00	0.01	0.00	0.01	0.00	0.00	0.00	0.00
BLE24	0.00	0.02	0.20	0.00	0.00	0.00	0.72	0.00	0.00	0.00	0.00	0.04	0.00	0.00	0.00	0.00	0.00	0.00
BLE25	0.00	0.01	0.51	0.00	0.00	0.00	0.45	0.00	0.00	0.00	0.00	0.04	0.00	0.00	0.00	0.00	0.00	0.00
BLE26	0.00	0.00	0.82	0.00	0.00	0.00	0.14	0.00	0.00	0.00	0.00	0.04	0.00	0.00	0.00	0.00	0.00	0.00
BLE27	0.00	0.01	0.50	0.00	0.00	0.00	0.47	0.00	0.00	0.00	0.00	0.02	0.00	0.00	0.00	0.00	0.00	0.00
BLE28	0.00	0.02	0.80	0.00	0.00	0.00	0.08	0.00	0.00	0.00	0.00	0.18	0.00	0.00	0.00	0.00	0.00	0.00
BLE29	0.00	0.01	0.82	0.00	0.00	0.00	0.12	0.00	0.00	0.00	0.00	0.02	0.00	0.00	0.00	0.00	0.00	0.00
BLE30	0.00	0.00	0.88	0.00	0.00	0.00	0.10	0.00	0.00	0.00	0.00	0.02	0.00	0.00	0.00	0.00	0.00	0.00
BLE31	0.00	0.00	0.88	0.00	0.00	0.00	0.10	0.00	0.00	0.00	0.00	0.01	0.00	0.00	0.00	0.00	0.00	0.00
BLE3	0.00	0.08	0.18	0.00	0.00	0.00	0.27	0.00	0.00	0.00	0.00	0.50	0.00	0.00	0.00	0.00	0.00	0.00
BLE32	0.00	0.02	0.42	0.00	0.00	0.00	0.08	0.11	0.00	0.00	0.00	0.10	0.00	0.00	0.00	0.00	0.00	0.00
BLE33	0.00	0.02	0.62	0.00	0.00	0.00	0.34	0.00	0.00	0.00	0.00	0.01	0.00	0.00	0.00	0.00	0.00	0.00
BLE34	0.00	0.01	0.88	0.00	0.00	0.00	0.08	0.00	0.00	0.00	0.00	0.01	0.00	0.00	0.00	0.00	0.00	0.00
BLE35	0.00	0.00	0.73	0.00	0.00	0.00	0.21	0.00	0.00	0.00	0.00	0.01	0.00	0.00	0.00	0.00	0.00	0.00
BLE36	0.00	0.00	0.77	0.00	0.00	0.00	0.21	0.00	0.00	0.00	0.00	0.02	0.00	0.00	0.00	0.00	0.00	0.00
BLE37	0.00	0.02	0.04	0.00	0.00	0.00	0.72	0.00	0.00	0.00	0.00	0.01	0.00	0.00	0.00	0.00	0.00	0.00
BLE38	0.07	0.02	0.11	0.00	0.00	0.00	0.70	0.00	0.00	0.00	0.00	0.02	0.00	0.00	0.00	0.00	0.00	0.00
BLE39	0.00	0.00	0.42	0.00	0.00	0.00	0.88	0.00	0.00	0.00	0.00	0.01	0.00	0.00	0.00	0.00	0.00	0.00
BLE40	0.00	0.01	0.51	0.00	0.00	0.00	0.48	0.00	0.00	0.00	0.00	0.11	0.00	0.00	0.00	0.00	0.00	0.00
BLE41	0.00	0.00	0.22	0.00	0.00	0.00	0.41	0.01	0.00	0.00	0.00	0.08	0.00	0.00	0.00	0.00	0.00	0.00
BLE4	0.00	0.00	0.01	0.00	0.00	0.00	0.18	0.00	0.00	0.00	0.00	0.77	0.00	0.00	0.00	0.00	0.00	0.00
BLE42	0.00	0.00	0.71	0.00	0.00	0.00	0.28	0.00	0.00	0.00	0.00	0.01	0.00	0.00	0.00	0.00	0.00	0.00
BLE43	0.00	0.01	0.07	0.00	0.00	0.00	0.00	0.00	0.00	0.00	0.00	0.02	0.00	0.00	0.00	0.00	0.00	0.00
BLE44	0.00	0.01	0.67	0.00	0.00	0.00	0.24	0.00	0.00	0.00	0.00	0.08	0.00	0.00	0.00	0.00	0.00	0.00
BLE45	0.00	0.00	0.88	0.00	0.00	0.00	0.12	0.00	0.00	0.00	0.00	0.00	0.00	0.00	0.00	0.00	0.00	0.00
BLE46	0.00	0.00	0.12	0.00	0.00	0.00	0.18	0.00	0.00	0.00	0.00	0.60	0.00	0.00	0.00	0.00	0.00	0.00
BLE47	0.00	0.01	0.42	0.00	0.00	0.00	0.51	0.00	0.00	0.00	0.00	0.06	0.00	0.00	0.00	0.00	0.00	0.00
BLE48	0.00	0.00	0.01	0.00	0.00	0.00	0.88	0.00	0.00	0.00	0.00	0.11	0.00	0.00	0.00	0.00	0.00	0.00
BLE49	0.00	0.00	0.47	0.00	0.00	0.00	0.02	0.01	0.00	0.00	0.00	0.00	0.00	0.00	0.00	0.00	0.00	0.00
BLE50	0.00	0.00	0.88	0.00	0.00	0.00	0.00	0.00	0.00	0.00	0.00	0.02	0.00	0.00	0.00	0.00	0.00	0.00
BLE5	0.00	0.01	0.00	0.00	0.00	0.01	0.42	0.00	0.00	0.00	0.00	0.22	0.00	0.00	0.01	0.01	0.00	0.00
BLE6	0.00	0.04	0.82	0.00	0.00	0.01	0.08	0.00	0.00	0.00	0.00	0.18	0.01	0.00	0.00	0.00	0.00	0.00
BLE7	0.00	0.08	0.12	0.00	0.00	0.00	0.60	0.00	0.00	0.00	0.00	0.21	0.00	0.00	0.00	0.00	0.00	0.00
BLE8	0.00	0.14	0.00	0.00	0.00	0.00	0.10	0.00	0.00	0.00	0.00	0.28	0.00	0.00	0.00	0.00	0.00	0.00
BLE9	0.00	0.00	0.01	0.00	0.00	0.00	0.04	0.00	0.00	0.00	0.00	0.28	0.00	0.00	0.00	0.00	0.00	0.00

Tab. S3 Detectable bacterial genus and its relative abundance

[illegible]

[illegible]

[illegible]	[illegible]

[illegible]

[illegible]

[illegible]

[illegible]

[illegible]

[illegible]

[illegible]

Tab. S[illegible] SLE patient's characteristics

Patient ID	Gender	Age (yrs)	Disease duration (yrs)	With LN at any time	SLEDAI score	LN duration (yrs)	With LN at time of collection	Immunological features				Medication usage	
								AnuA	Anti-nRNP	Anti-[illegible]	Other immunological features	HCQ dosage (mg/d)	Pred dosage(mg/d)
SLE1	Female	33	5	0	4	No	0	−	−	+		0.2	5.0
SLE10	Female	34	3	1	10	3	1	+	−	−	[illegible], IgG, RF	0.2	10.0
SLE11	Male	61	0.5	0	0	No	1	−	−	−	[illegible]	0.2	5.0
SLE12	Female	32	7	1	6	7	1	−	+	−	[illegible]	0.2	5.0
SLE13	Female	38	12	1	3	12	1	+	−	−	[illegible]	0.2	10.0
SLE14	Female	33	5	1	0	2	1	+	−	−		0.2	5.0
SLE15	Female	27	12	1	11	2	0	−	+	−	[illegible]	0.2	10.0
SLE16	Female	27	2	1	28	2	0	+	−	−	[illegible]	0.2	10.0
SLE17	Female	60	11	0	4	No	1	−	+	+	[illegible]	0.2	5.0
SLE18	Female	62	22	1	10	2	0	−	+	−	[illegible]	0.2	5.0
SLE19	Female	21	4	1	14	4	0	−	−	−		0.2	5.0
SLE2	Female	44	5	1	6	5	0	−	+	−	[illegible]	0.2	5.0
SLE20	Female	32	5	1	10	3	2	−	−	−	[illegible]	0.2	10.0
SLE21	Female	58	12	1	4	2	1	−	−	+	[illegible]	0.2	5.0
SLE22	Female	47	22	1	18	2	0	+	−	−	[illegible], IgG, RF	0.2	10.0
SLE23	Female	30	7	1	10	7	1	−	+	+	[illegible]	0.2	10.0
SLE24	Female	46	5	1	20	3	0	+	−	+	[illegible], IgG, RF	0.2	5.0
SLE25	Female	57	5	1	4	3	0	+	−	−	[illegible], IgG, RF	0.2	5.0
SLE26	Female	56	5	1	14	3	1	−	+	+	[illegible]	0.2	5.0
SLE27	Female	57	5	1	24	0.02	1	+	−	−	[illegible]	0.2	10.0
SLE28	Female	68	14	0	4	No	1	−	−	−	[illegible]	0.2	5.0
SLE29	Female	33	1	1	22	0.8	1	−	+	−	[illegible], IgG, RF	0.2	5.0
SLE3	Female	65	5	0	12	No	1	+	−	−		0.2	5.0
SLE30	Female	58	0.3	1	24	0.08	1	−	−	−	[illegible], IgG, RF	0.2	5.0
SLE31	Female	41	4	1	3	1	0	−	−	−	[illegible]	0.2	5.0
SLE32	Female	41	5	1	10	2	0	+	+	−		0.2	10.0
SLE33	Female	30	6	1	16	6	1	+	−	+	[illegible]	0.2	5.0
SLE34	Female	47	22	1	18	0	0	−	+	+		0.2	5.0
SLE35	Female	62	16	0	12	No	0	+	+	+	[illegible]	0.2	10.0
SLE36	Female	50	5	1	20	0	1	−	−	−	[illegible]	0.2	5.0
SLE37	Female	47	7	1	10	7	1	+	−	+		0.2	5.0
SLE38	Female	30	2	1	11	2	1	+	+	−	[illegible]	0.2	10.0
SLE39	Female	28	17	1	20	7	0	+	−	+	[illegible], IgG, RF	0.2	5.0
SLE4	Female	62	12	0	12	No	1	+	−	+	[illegible]	0.4	5.0
SLE40	Female	28	1	1	16	1	0	+	−	+	[illegible]	0.2	5.0
SLE41	Female	22	14	1	22	6	0	+	+	+	[illegible]	0.4	10.0
SLE42	Female	32	18	1	37	10	0	+	+	+	[illegible]	0.4	5.0
SLE43	Female	28	4	1	28	4	0	+	−	−	[illegible]	0.4	5.0
SLE44	Female	48	20	1	16	18	1	+	+	+	[illegible]	0.4	10.0
SLE45	Female	56	5	1	11	2	0	+	+	−	[illegible]	0.4	5.0
SLE46	Male	33	1	1	18	1	1	+	−	−	[illegible]	0.4	5.0
SLE47	Female	62	2	1	18	2	0	−	+	−	[illegible]	0.4	10.0
SLE48	Female	57	5	1	12	3	0	−	−	+	[illegible]	0.4	5.0
SLE49	Male	72	12	0	7	No	1	+	−	−	[illegible], IgG, RF	0.4	10.0
SLE5	Male	67	28	0	10	No	1	−	−	−	[illegible]	0.4	5.0
SLE50	Male	58	2	1	10	2	0	+	−	+	RF	0.4	10.0
SLE6	Female	20	4	0	3	No	0	−	−	+	[illegible]	0.4	10.0
SLE7	Female	50	7	0	18	No	0	−	−	+	[illegible]	0.4	10.0
SLE8	Female	58	7	0	0	No	0	−	−	−	[illegible]	0.4	5.0
SLE9	Male	32	6	1	0	4	0	+	−	−	[illegible]	0.4	5.0

Abbreviations: "−" and "+" represent negative and positive, respectively; [illegible]

Tab. S7 Comparison of nutrient intake between controls and SLE

Nutrient intake	Controls (n = 50)	SLE (n = 50)	*P value*
Calcium (mg/d)	430.81 ± 256.02	1223.33 ± 472.72	< 0.001
Carbohydrate (g/d)	247.50 ± 73.32	217.64 ± 78.79	0.053
Carotene(μg/d)	17.59 ± 4.22	20.1 ± 8.40	0.063
Cholesterol (mg/d)	527.42 ± 188.43	516.16 ± 235.38	0.792
Copper (mg/d)	2.20 ± 0.65	2.06 ± 1.12	0.478
Energy (Kcal/d)	2191.03 ± 308.2	2048.09 ± 478.82	0.080
Fat (g/d)	116.62 ± 149.62	97.52 ± 46.84	0.391
Fiber (g/d)	12.04 ± 8.59	13.93 ± 8.34	0.269
Iron (mg/d)	30.31 ± 16.64	25.14 ± 16.05	0.117
Magnesium (mg/d)	243.91 ± 76.91	277.58 ± 94.16	0.053
Manganese (mg/d)	5.77 ± 2.63	5.03 ± 2.44	0.147
Niacin (mg/d)	15.78 ± 4.46	17.7 ± 6.39	0.084
Phosphorus (mg/d)	776.72 ± 195.95	843.82 ± 304.34	0.194
Potassium (mg/d)	1667.48 ± 579.82	1874.99 ± 547.99	0.069
Protein (g/d)	69.53 ± 18.34	80.64 ± 41.53	0.088
Retinol (μg/d)	1004.76 ± 299.76	1121.91 ± 378.41	0.089
Selenium (mg/d)	44.24 ± 16.67	49.00 ± 20.69	0.208
Sodium (mg/d)	2207.3 ± 416.01	2401.04 ± 664.25	0.084
Vitamin A (μg/d)	1106.72 ± 518.13	1398.03 ± 908.65	0.052
Vitamin B1 (mg/d)	0.82 ± 0.39	0.97 ± 0.43	0.076
Vitamin B2 (mg/d)	1.27 ± 0.6	1.48 ± 0.61	0.073
Vitamin C (mg/d)	84.93 ± 39.66	99.05 ± 52.96	0.135
Vitamin E (mg/d)	66.66 ± 12.77	60.48 ± 23.79	0.110
Zinc (mg/d)	9.71 ± 3.55	11.49 ± 4.19	0.024

Student's *t* test on normalized continuous variables and Wilcoxon rank-sum test was used on un-normalized continuous variables.

Tab. S8 Bacterial taxonomy affected by food intake

Variable	Taxonomy	Coefficient	*p* value	*p (adj)* value
Calcium	*Blautia wexlerae*	0.000	0.002	0.017
	Alistipes	0.000	0.002	0.018
Prednisone	*Blautia wexlerae*	0.006	0.000	0.001

MaAsLin (Microbiome Multivariable Associations with Linear Models) was used to adjust confounding factors, food intake, on bacteria showing significant difference in their abundance using Wilcox test (Galaxy Version [illegible]). P value was adjusted using Benjamini Hochberg false discovery rate (FDR).

Tab. S9 Demographics of LN and non-LN patients

Parameters	Controls (n = 50)	LN (n = 38)	non-LN (n = 12)	Value for cohort (n a) b or statistic		
				Controls vs LN	Controls vs nonLN	LN vs non-LN
Female sex, n (%)	44 (88)	35 (92.11)	9 (75.00)	0.529	0.251	0.141
Age (yrs)	49.22 ± 15.19	42.03 ± 15.45	54.42 ± 14.66	0.023	0.289	0.009
Duration of SLE (yrs)	NA	8.06 ± 6.12	10.03 ± 6.57	NA	NA	0.343
SLEDAI	NA	14.03 ± 5.88	9.42 ± 3.32	NA	NA	0.002
Duration of LN (yrs)	NA	5.30 ± 3.60	NA	NA	NA	NA
Body-mass index (kg/m^2)	23.98 ± 3.23	24.29 ± 2.84	23.04 ± 1.72	0.650	0.346	0.157
Comorbidity						
Diabetes, n (%)	5 (10)	4 (10.53)	1 (8.33)	0.936	1.000	1.000
Hypertension, n (%)	10 (20)	9 (23.68)	1 (8.33)	0.677	0.342	0.246
Immunological features						
Complement 3 (g/L)	1.20 ± 0.64	0.74 ± 0.24	0.84 ± 0.18	<0.001	0.061	0.194
Complement 4 (g/L)	0.30 ± 0.26	0.15 ± 0.06	0.17 ± 0.05	<0.001	0.002	0.213
Ig A (g/L)	2.59 ± 0.85	2.65 ± 1.19	2.44 ± 0.68	0.751	0.581	0.553
Ig G (g/L)	10.98 ± 1.88	16.44 ± 8.78	14.49 ± 3.64	0.001	0.007	0.459
Ig M (g/L)	1.17 ± 0.51	1.03 ± 0.56	0.95 ± 0.28	0.198	0.152	0.670
ESR（mm/hr）*	NA	32.40 ± 24.27	22.48 ± 14.30	NA	NA	0.187
Renal function						
Serum creatinine (μmol/L)	55.23 ± 10.75	61.62 ± 26.21	75.81 ± 67.78	0.174	0.340	0.504
Blood urea nitrogen (mmol/L)	5.73 ± 5.99	5.46 ± 2.25	5.47 ± 1.38	0.841	0.886	0.943
Serum uric acid (umol/L)	268.04 ± 72.56	327.87 ± [illegible]	290.33 ± 62.75	0.014	0.411	0.209
Estimated glomerular filtration rate (mL/min/1.73 m^2)	114.40 ± 20.08	111.09 ± 38.53	101.29 ± 38.15	0.635	0.292	0.462
Urinary creatinine (μmol/L)	120.92 ± 17.13	126.65 ± 18.25	121.17 ± 32.26	0.134	0.980	0.584
Urine analysis						
White blood cells (/uL)	2.22 ± 6.38	10.63 ± 25.04	5.16 ± 10.06	<0.001	0.002	0.799
Red blood cells (/uL)	0.61 ± 3.03	28.83 ± 145.84	25.35 ± 73.63	<0.001	<0.001	0.861
Nitrites positive, n (%)	1 (2)	2 (5.26)	0 (0.00)	0.576	1.000	1.000
Leucocyte esterase, n (%)	0 (0)	11 (0.29)	1 (8.33)	<0.001	0.194	0.145

[a] n, number of subjects;
[b] Mean ± SD or n (%);
[c] Pearson Chi-square or Fisher's exact test was used with categorical variables; Wilcoxon rank-sum test was used on un-normalized continuous variables.
Abbreviations: LN, lupus nephritis; NA, not applicable; SLEDAI, systemic lupus erythematosus disease activity index

[illegible]

[illegible]

Tab. S11 Metabolites showing Fold change>2 or <0.5 (Controls vs ELS)

Metabolite	Fold Change	log2(FC)
(-)-Riboflavin	[illegible]	[illegible]
(3alpha)-Androst-5-en-17-one	[illegible]	[illegible]
[illegible]	[illegible]	[illegible]
1,3-Cyclohexanedione	[illegible]	[illegible]
11alpha-Hydroxyprogesterone beta-D-glucuronide	[illegible]	[illegible]
[illegible]	[illegible]	[illegible]
[illegible]	[illegible]	[illegible]
11-Oxo-androsterone glucuronide	[illegible]	[illegible]
[illegible]	[illegible]	[illegible]
[illegible]	[illegible]	[illegible]
[illegible]	[illegible]	[illegible]
[illegible]	[illegible]	[illegible]
[illegible]	[illegible]	[illegible]
[illegible]	[illegible]	[illegible]
[illegible]	[illegible]	[illegible]
[illegible]	[illegible]	[illegible]
[illegible]	[illegible]	[illegible]
[illegible]	[illegible]	[illegible]
[illegible]	[illegible]	[illegible]
[illegible]	[illegible]	[illegible]
[illegible]	[illegible]	[illegible]
[illegible]	[illegible]	[illegible]
[illegible]	[illegible]	[illegible]
[illegible]	[illegible]	[illegible]
[illegible]	[illegible]	[illegible]
[illegible]	[illegible]	[illegible]
[illegible]	[illegible]	[illegible]

[illegible]

Tab. S13 Metabolites with VIP >1 (Controls vs SLE)

Metabolite	VIP
11-beta-Hydroxyandrosterone-3-glucuronide	1.159
11-Methyl-7-oxatetracyclo[6.3.1.01,6.04,11]dodecane	1.812
12-Hydroxy-13-O-D-glucuronoside-octadec-9Z-enoate	1.111
16alpha-hydroxydehydroepiandrosterone3-sulfate	1.502
1H-Indole-1-carboxamide, 6-chloro-2,3-dihydro-5-methyl-N-[6-[(2-methyl-3-pyridinyl)oxy]-3-pyridinyl]-	1.076
1'-Hydroxymidazolam .beta.-D-glucuronide	3.558
3-Hydroxy-2-(4-methylbenzoyl)-4H-1-benzopyran-4-one	1.490
4,5-Dihydro-drospirenone-3-sulfate	1.209
5.alpha.-Androstan-3.beta.-ol-17-one sulfate	1.112
7,8-dihydroxy-2H-chromen-2-one	1.329
Acesulfame	1.033
Acetyl-DL-carnitine	1.002
Acylcarnitine 18:5	1.409
Acylcarnitine 21:5	1.726
Androsterone glucuronide	1.011
Benzenesulfonic acid	2.291
Celastrol	1.207
Clozapine	1.292
Clozapine glucuronide	1.266
Codeine	1.130
Cortolone-3-glucuronide	1.167
Cyclamate	1.252
cyclic N-Acetylserotonin glucuronide	2.029
Desethylchloroquine	1.455
Diethyl sebacate	1.018
Gibberellin A92	1.538
Hydroxychloroquine	2.094
Lidocaine	2.028
LysoPA 19:2; LysoPA 19:2	1.077
Olopatadine	1.799
PC(16:0/16:1(9Z))	1.464
PG 18:0; PG(2:0/16:0)	1.024
PG 18:0; PG(9:0/9:0)	1.002
Phloretin	1.668
Propofol .beta.-D-glucuronide	3.799
S-Adenosyl-L-methionine	1.534
Styrene	1.616
Sulfamethazine	2.419
Taurocholate	1.627
TG 36:6; TG(12:2/12:2/12:2)	1.384
TG 46:10; TG(12:3/15:2/19:5)	1.582
Tryptophyl-Phenylalanine	1.066

VIP was calculated using PLS-DA analysis
Abbreviation: VIP, Variable Importance in Projection

Tab. S11 The effects of nutrient intake on metabolites showing significant different between controls and BLC

Parameters	B	SE	Wald	df	*P* value	OR	95% CI (lower-upper)
Calcium	0.002	0.001	2.983	1	0.084	1.002	1.000~1.004
Zinc	0.098	0.078	1.59	1	0.207	1.103	0.947~1.284
11-beta-Hydroxyandrosterone -3-glucuronide	-0.001	0	13.346	1	0	0.999	0.998~0.999
Constant	-1.108	1.103	1.009	1	0.315	0.327	
Calcium	0.002	0.001	1.159	1	0.282	1.002	0.998~1.004
Zinc	-0.004	0.130	0.383	1	0.360	0.91	0.871~1.220
11-Methyl-7-azabicyclo[2.2.1.02,6.03,11]dodecane	-0.178	0.054	10.272	1	0.001	0.839	0.754~0.933
Constant	3.373	2.428	2.187	1	0.141	30.283	
Calcium	0.004	0.001	7.073	1	0.030	1.004	1.001~1.007
Zinc	-0.037	0.134	0.330	1	0.83	0.964	0.779~1.108
12-Hydroxy-12-O-D-glucuronide -abscisic-OC-enate	0.01	0.003	8.080	1	0.003	1.01	1.003~1.018
Constant	-0.098	1.349	13.114	1	0	0.004	
Calcium	0.002	0.001	4.283	1	0.032	1.002	1.000~1.004
Zinc	0.117	0.088	1.8	1	0.18	1.120	0.947~1.330
16alpha-hydroxydehydroepiandrosterone 3-sulfate	-0.002	0	13.866	1	0	0.998	0.997~0.999
Constant	-1.273	1.288	1.467	1	0.228	0.237	
Calcium	0.002	0.001	2.893	1	0.083	1.002	1.000~1.004
Zinc	0.02	0.133	0.018	1	0.890	1.021	0.831~1.234
1H-Indole-1-carboxamide, 2-chloro-2,3-dihydro-2-methyl-N-[3-[(2-methyl)-2-pyridyl	-0.038	0.011	12.212	1	0	0.962	0.941~0.983
Constant	1.098	1.737	0.88	1	0.348	3.187	
Calcium	-0.001	0.073	0	1	0.983	0.999	0.880~1.102
Zinc	-0.073	8.271	0.083	1	0.944	0.389	0~71422709.883
1-Hydroxymidazolam beta-D-glucuronide	-4.001	3.385	3.089	1	0.079	0.01	0~128224.989
Constant	39.013	128.38	0.091	1	0.764	7.17083E+23	
Calcium	0	0.002	0.003	1	0.983	1	0.996~1.003
Zinc	0.179	0.17	1.107	1	0.283	1.190	0.857~1.659
2-Hydroxy-2-(1-methylbutenyl)-6H-1-benzopyran-4-one	0.03	0.012	10.980	1	0.001	1.031	1.021~1.082
Constant	-8.34	3.164	6.208	1	0.011	0	
Calcium	0.003	0.002	4.330	1	0.038	1.003	1.000~1.006
Zinc	0.004	0.122	0.001	1	0.974	1.030	0.832~1.34
4,5-Dihydro-aldosterone-3-sulfate	0.012	0.004	9.07	1	0.003	1.012	1.004~1.02
Constant	-7.081	2.018	16.44	1	0	0	
Calcium	0.003	0.001	11.383	1	0.001	1.003	1.001~1.003
Zinc	0.112	0.076	2.127	1	0.142	1.118	0.963~1.298
5alpha-Androstan-3beta-ol-17-one sulfate	0	0	2.734	1	0.010	1	0.999~1.000
Constant	-0.281	1.178	7.79	1	0.003	0.038	
Calcium	0.003	0.002	1.227	1	0.23	1.003	0.998~1.003
Zinc	0.300	0.188	2.733	1	0.023	1.428	0.990~2.078
7,8-dihydroxy-OH-abramen-2-one	0.227	0.094	7.974	1	0.003	1.263	1.082~1.271
Constant	-12.003	4.023	7.060	1	0.008	0	
Calcium	0.003	0.001	13.310	1	0	1.003	1.002~1.007
Zinc	0.021	0.082	0.067	1	0.790	1.022	0.869~1.201
Acetaldiene	0.009	0.003	7.829	1	0.003	1.009	1.003~1.013
Constant	-2.489	1.327	38.13	1	0	0.004	
Calcium	0.004	0.001	12.843	1	0	1.004	1.002~1.008
Zinc	0.183	0.080	4.002	1	0.034	1.201	1.014~1.423
Acetyl-DL-carnitine	-0.001	0	12.381	1	0	0.999	0.998~1.000
Constant	-0.013	1.338	8.783	1	0.003	0.02	
Calcium	0.003	0.001	8.330	1	0.003	1.003	1.001~1.003
Zinc	0.003	0.09	0.329	1	0.880	1.203	0.883~1.236
Acylcarnitine 18:3	-0.007	0.014	22.183	1	0	0.933	0.908~0.961
Constant	-1.408	1.403	1.039	1	0.287	0.224	
Calcium	0.002	0.001	2.843	1	0.080	1.002	1.000~1.003
Zinc	-0.008	0.118	0.003	1	0.944	0.992	0.783~1.230
Acylcarnitine 22:3	-0.034	0.013	8.114	1	0.004	0.937	0.903~0.987
Constant	1.013	1.802	0.32	1	0.572	2.789	
Calcium	0.003	0.001	8.49	1	0.011	1.003	1.001~1.003
Zinc	0.133	0.081	2.380	1	0.103	1.142	0.974~1.338
Androsterone glucuronide	0	0	8.048	1	0.002	1	0.999~1.000
Constant	-0.278	1.321	2.979	1	0.084	0.102	
Calcium	0.002	0.001	2.888	1	0.340	1.002	1.000~1.003
Zinc	-0.027	0.133	0.038	1	0.794	0.973	0.782~1.198
Benzenesulfonic acid	-0.137	0.043	8.237	1	0.002	0.872	0.798~0.952
Constant	0.733	1.033	0.333	1	0.031	2.083	
Calcium	0.003	0.001	10.322	1	0.001	1.003	1.001~1.003
Zinc	0.128	0.08	2.381	1	0.107	1.137	0.972~1.328
Calcitriol	-0.001	0	7.73	1	0.003	0.999	0.998~1.000
Constant	-3.28	1.24	7.009	1	0.008	0.037	
Calcium	0.003	0.001	10.4	1	0.001	1.003	1.001~1.003
Zinc	0.104	0.084	1.303	1	0.213	1.11	0.942~1.307
Cinnoptine	-0.012	0.004	10.130	1	0.001	0.988	0.981~0.993
Constant	-0.738	1.232	4.782	1	0.029	0.083	
Calcium	0.002	0.001	3.403	1	0.064	1.002	1.000~1.004
Zinc	0.138	0.102	1.862	1	0.173	1.148	0.941~1.401
Cinnoptine glucuronide	-0.008	0.017	11.130	1	0.001	0.943	0.911~0.973
Constant	-0.411	1.471	0.078	1	0.78	0.663	
Calcium	0.004	0.001	11.084	1	0.001	1.004	1.001~1.008
Zinc	0.112	0.083	1.780	1	0.18	1.118	0.95~1.318
Dehalene	-0.06	0.014	18.932	1	0	0.942	0.917~0.968
Constant	-0.196	1.294	2.870	1	0.09	0.111	
Calcium	0.002	0.001	2.979	1	0.084	1.002	1.000~1.004
Zinc	0.143	0.089	2.63	1	0.104	1.130	0.971~1.378
Dehalene-3-glucuronide	-0.001	0	12.930	1	0	0.999	0.998~1.000
Constant	-1.127	1.308	0.079	1	0.41	0.324	
Calcium	0.002	0.001	1.832	1	0.172	1.002	0.999~1.004
Zinc	-0.018	0.118	0.019	1	0.89	0.984	0.781~1.24
epsilon-N-Acetyllysine glucuronide	-0.004	0.008	8.881	1	0.003	0.970	0.951~0.990
Constant	2.033	1.050	1.07	1	0.304	7.638	
Calcium	0.004	0.003	2.028	1	0.134	1.004	0.999~1.008
Zinc	0.308	0.187	2.742	1	0.098	1.383	0.942~1.804
Desethylchloroquine	0.041	0.013	9.73	1	0.002	1.042	1.013~1.068
Constant	-11.813	4.082	8.41	1	0.011	0	
Calcium	0.003	0.002	3.3	1	0.069	1.003	1.000~1.007
Zinc	0.113	0.117	0.928	1	0.333	1.119	0.890~1.408
Diethyl adipate	0.148	0.058	0.982	1	0.002	1.127	1.057~1.238
Constant	-8.213	2.307	12.048	1	0	0	
Calcium	0.002	0.001	1.721	1	0.19	1.002	0.999~1.004
Zinc	-0.008	0.121	0.030	1	0.940	0.992	0.783~1.237
Gibberellin A96	-0.06	0.024	6.17	1	0.013	0.942	0.898~0.988
Constant	1.834	1.829	1.029	1	0.31	6.287	
Calcium	0.003	0.003	1.340	1	0.204	1.003	0.998~1.008
Zinc	0.037	0.193	2.080	1	0.083	1.029	0.978~2.087
Hydroxychloroquine	0.138	0.032	8.310	1	0.003	1.172	1.008~1.287
Constant	-11.308	4.037	8.087	1	0.014	0	
Calcium	0.003	0.001	8.734	1	0.008	1.003	1.001~1.003
Zinc	0.124	0.083	2.094	1	0.148	1.132	0.957~1.338
LysoPA 18:0; LysoPA 18:0	-0.008	0.003	8.830	1	0.003	0.992	0.987~0.997
Constant	-2.184	1.331	2.374	1	0.108	0.113	
Calcium	0.003	0.001	8.770	1	0.009	1.003	1.001~1.004
Zinc	0.108	0.077	1.880	1	0.189	1.112	0.950~1.294
PC(16:0/18:4(PE))	0.008	0.014	8.982	1	0.008	1.037	1.008~1.004
Constant	-4.837	1.288	14.140	1	0	0.009	
Calcium	0.003	0.001	8.840	1	0.003	1.003	1.001~1.003
Zinc	0.073	0.078	0.88	1	0.322	1.078	0.928~1.232
PE 18:0; PE(2:0/16:0)	-0.004	0.002	7.011	1	0.008	0.993	0.993~0.999
Constant	-2.207	1.323	2.784	1	0.093	0.11	
Calcium	0.003	0.001	8.308	1	0.003	1.003	1.001~1.003
Zinc	0.099	0.082	1.378	1	0.24	1.101	0.903~1.293
PE 18:0; PE(8:0/10:0)	-0.004	0.001	10.688	1	0.001	0.993	0.994~0.998
Constant	-2.181	1.283	2.777	1	0.090	0.118	
Calcium	0.001	0.001	1.230	1	0.280	1.001	0.999~1.004
Zinc	-0.03	0.118	0.083	1	0.803	0.971	0.770~1.224
Phloretin	-0.1	0.032	8.900	1	0.002	0.903	0.823~0.963
Constant	2.830	1.904	2.177	1	0.14	17.343	
Calcium	0.002	0.001	1.309	1	0.231	1.002	0.999~1.003
Zinc	0.02	0.148	0.018	1	0.894	1.02	0.763~1.363
S-Adenosyl-L-methionine	-0.132	0.087	7.480	1	0.030	0.870	0.797~0.904
Constant	3.703	2.388	2.117	1	0.140	40.589	
Calcium	0.001	0.002	0.709	1	0.380	1.001	0.998~1.004
Zinc	0.12	0.194	0.384	1	0.483	1.127	0.817~1.334
Styrene	-0.498	0.218	2.181	1	0.023	0.089	0.397~0.933
Constant	2.838	2.42	1.364	1	0.243	16.878	
Calcium	0.004	0.001	12.247	1	0	1.004	1.002~1.008
Zinc	0.003	0.084	0.001	1	0.330	1.030	0.882~1.202
Taurocholate	-0.013	0.003	10.388	1	0.001	0.983	0.977~0.994
Constant	-2.272	1.281	3.377	1	0.080	0.089	
Calcium	0.003	0.001	8.861	1	0.008	1.003	1.001~1.004
Zinc	0.112	0.078	2.030	1	0.152	1.118	0.960~1.303
TG 30:0; TG(10:0/10:0/10:0)	0.029	0.011	8.083	1	0.01	1.029	1.007~1.052
Constant	-4.823	1.292	14.031	1	0	0.007	
Calcium	0.003	0.001	8.302	1	0.01	1.003	1.001~1.004
Zinc	0.108	0.077	1.879	1	0.171	1.111	0.955~1.293
TG 43:10; TG(12:0/12:0/19:0)	0.043	0.017	8.421	1	0.011	1.044	1.010~1.080
Constant	-4.332	1.238	12.423	1	0	0.011	
Calcium	0.008	0.003	7.883	1	0.080	1.008	1.002~1.018
Zinc	0.123	0.133	0.871	1	0.803	1.133	0.865~1.502
Tryptophyl-Phenylalanine	0.131	0.042	9.874	1	0.002	1.14	1.050~1.237
Constant	-10.887	4.308	10.983	1	0.001	0	

Binary logistic regression model was used.
Abbreviations: B, coefficient value; SE, standard error; df, degrees of freedom; 95% CI, 95% confidence interval

Tab. S15 Effects of hydroxychloroquine intake on metabolites

Metabolites	0.2 (mg/d)	0.4 (mg/d)	$P_{(adj)}$ value
	mean ± SD	mean ± SD	
11-beta-Hydroxyandrosterone-3-glucuronide	611.09±767.64	809.13±1216.95	1.000
11-Methyl-7-oxatetracyclo[6.3.1.01,6.04,11]dodecane	5.4±6.74	3.57±4.85	1.000
12-Hydroxy-13-O-D-glucuronoside-octadec-9Z-enoate	1015.14±775.48	1174.86±1183.6	1.000
16alpha-hydroxydehydroepiandrosterone 3-sulfate	263.5±384.36	363.51±645.16	1.000
1H-Indole-1-carboxamide, 6-chloro-2,3-dihydro-5-methyl-N-[6-[(2-methyl-3-pyridinyl)oxy]-3-pyridinyl]-	38.06±15.36	40.43±22.28	1.000
1'-Hydroxymidazolam .beta.-D-glucuronide	3.98±2.03	4.01±2.33	1.000
3-Hydroxy-2-(4-methylbenzoyl)-4H-1-benzopyran-4-one	683.98±414.42	692.2±478.64	1.000
4,5-Dihydro-drospirenone-3-sulfate	2298.01±1860.55	2741.32±2239.52	1.000
5.alpha.-Androstan-3.beta.-ol-17-one sulfate	782.27±1470.06	1273.37±2648.54	1.000
7,8-dihydroxy-2H-chromen-2-one	107.25±73.61	99.95±73.12	1.000
Acesulfame	940.49±2004.68	926.79±2066.56	1.000
Acetyl-DL-carnitine	931.52±1074.33	1018.19±1225.31	1.000
Acylcarnitine 18:5	14.32±27.89	5.03±5.15	1.000
Acylcarnitine 21:5	15.53±16.12	13.28±11.8	1.000
Androsterone glucuronide	1737.11±1884.54	2292.25±3412.29	1.000
Benzenesulfonic acid	3.74±3.22	5.4±7.27	1.000
Celastrol	459.66±835.14	810.77±1619.18	1.000
Clozapine	25.49±45.04	39.55±57.25	1.000
Clozapine glucuronide	17.06±12.19	21.44±17.78	1.000
Codeine	17.86±30.5	12.87±15.01	1.000
Cortolone-3-glucuronide	898.89±1005.21	1236.16±1578.56	1.000
cyclic N-Acetylserotonin glucuronide	42.7±21.47	45.8±43.86	1.000
Desethylchloroquine	427.9±192.91	445.1±270.7	1.000
Diethyl sebacate	108.45±77.87	129.77±90.15	1.000
Gibberellin A92	14.86±9.33	13.36±6.74	1.000
Hydroxychloroquine	221.35±143.72	293.49±251.46	1.000
LysoPA 19:2; LysoPA 19:2	71.57±80.84	98.61±143.16	1.000
Olopatadine	73.64±45.36	95.8±77.26	1.000
PC(16:0/16:1(9Z))	1391.62±2960.64	2413.44±5478.25	1.000
PG 18:0; PG(2:0/16:0)	107.62±72.77	163.66±273.03	1.000
PG 18:0; PG(9:0/9:0)	154.87±158.27	174.05±210.52	1.000
Phloretin	13.81±5.11	14.24±7.48	1.000
S-Adenosyl-L-methionine	17.54±6.53	18.05±9.71	1.000
Styrene	4±1.76	4.61±1.95	1.000
Taurocholate	47.81±81.01	25.09±24.46	1.000
TG 36:6; TG(12:2/12:2/12:2)	2045.69±4366.62	3512.07±8110.84	1.000
TG 46:10; TG(12:3/15:2/19:5)	968.83±2042.34	1634.37±3676.38	1.000
Tryptophyl-Phenylalanine	316.4±242.68	339.73±394.72	1.000

Wilcoxon rank-sum test was used on the metabolites. *P* value was adjusted using Benjamin Hochberg false discovery rate (FDR).

Tab. S16 Effects of prednisone intake on metabolites

Metabolite	0 mg	5 mg	10 mg	P_{adj} value		
	mean±SD	mean±SD	mean±SD	0 mg vs 5	0 mg vs 10	5 mg vs 10 mg
11-beta-Hydroxyandrosterone-3-glucuronide	990.56±887	596.94±991.98	790.71±1086.59	0.969	1.000	1.000
11-Methyl-7-oxatetracyclo[6.3.1.01,6.04,11]dodecane	2.78±1.74	5.75±7.05	3.41±4.98	0.996	1.000	1.000
12-Hydroxy-13-O-D-glucuronoside-octadec-9Z-enoate	1100.7±1009.15	1026.82±986.79	1225.76±961.49	0.996	1.000	1.000
16alpha-hydroxydehydroepiandrosterone 3-sulfate	524.51±420.66	206.27±461.48	407.27±595.18	0.969	1.000	1.000
1H-Indole-1-carboxamide, 6-chloro-2,3-dihydro-5-methyl-N-[6-[(2-methyl-3-pyridinyl)oxy]-3-pyridinyl]-	28.92±19.99	28.81±18.21	29.13±18.98	0.996	1.000	1.000
1'-Hydroxymidazolam .beta.-D-glucuronide	3.42±2.1	4.19±2.57	2.89±2	0.996	1.000	1.000
3-Hydroxy-2-(4-methylbenzoyl)-4H-1-benzopyran-4-one	372±366.85	801.49±602.62	607.06±475.58	0.969	1.000	1.000
4,5-Dihydro-drospirenone-3-sulfate	1733.79±1895.7	2192.62±1904.64	2107.22±2085.71	0.996	1.000	1.000
5.alpha.-Androstan-3.beta.-ol-17-one sulfate	1060.14±735.52	752.11±1967.96	1481.91±2502.78	0.969	1.000	1.000
7,8-dihydroxy-2H-chromen-2-one	88.54±68.27	115.52±78.27	96.05±68.15	0.985	1.000	1.000
Acesulfame	994.26±1576.86	992.29±1971.04	979.21±2280.92	0.996	1.000	1.000
Acetyl-DL-carnitine	646.21±665.62	1128.72±1165.19	888.42±1160.23	0.996	1.000	1.000
Acylcarnitine 18:5	29.68±67.17	9.79±18.54	4.95±4.66	0.969	1.000	1.000
Acylcarnitine 21:5	10.65±5.52	16.41±16.75	12.68±12.25	0.996	1.000	1.000
Androsterone glucuronide	2641.22±1528.56	1587.22±2478.16	2449.88±2159.49	0.969	1.000	1.000
Benzenesulfonic acid	5.2±0.85	4.6±6.42	4.52±2.97	0.996	1.000	1.000
Celastrol	525.99±279.16	488.5±1171.19	890.75±1500.8	0.996	1.000	1.000
Clozapine	95.81±115.45	19.1±14.78	28.74±22.59	0.985	1.000	1.000
Clozapine glucuronide	24.82±27.17	16.15±9.64	18.13±12.76	0.969	1.000	1.000
Codeine	22.14±60.36	14.05±26.19	11.73±12.93	0.996	1.000	1.000
Cortolone-3-glucuronide	1819.59±1414.94	825.21±1146.2	1142.07±1402.62	0.969	1.000	1.000
cyclic N-Acetylserotonin glucuronide	28.92±22.82	50.45±98.81	26.36±24.05	0.996	1.000	1.000
Desethylchloroquine	250.71±120.55	496±227.27	290.55±200.25	0.969	1.000	1.000
Diethyl sebacate	96.15±81.63	104.41±75.51	142.09±86.64	0.996	1.000	1.000
Gibberellin A92	11.28±6.52	16.32±9.97	11.92±4.52	0.985	1.000	1.000
Hydroxychloroquine	109.29±76.4	274.19±201.52	249±198.87	0.969	1.000	1.000
LysoPA 19:2; LysoPA 19:2	101.05±87	69.72±108.22	101.92±129.57	0.969	1.000	1.000
Olopatadine	29±25.13	88.52±81.26	84.13±62.59	0.969	1.000	1.000
PC(16:0/16:1(9Z))	420.94±882.27	1839.18±4200.1	2141.06±6611.49	0.996	1.000	1.000
PG 18:0; PG(2:0/16:0)	325.25±452.08	91.2±71.28	126.15±102.22	0.969	1.000	1.000
PG 18:0; PG(9:0/9:0)	259.76±156.22	124.52±175.94	179.49±195.28	0.969	1.000	1.000
Phloretin	14.48±4.96	12.42±7.21	14.18±4.44	0.996	1.000	1.000
S-Adenosyl-L-methionine	12.77±5.17	19.12±9.73	16.96±4.58	0.996	1.000	1.000
Styrene	2.94±1.45	4.5±1.94	2.91±1.75	0.996	1.000	1.000
Taurocholate	22.02±12.41	52.72±84.73	26.46±24.91	0.996	1.000	1.000
TG 36:6; TG(12:2/12:2/12:2)	617.59±1901.88	2722.45±6452.12	2082.1±6392.65	0.996	1.000	1.000
TG 46:10; TG(12:3/15:2/19:5)	208.58±645.95	1275.97±2982.59	1447.25±2923.45	0.996	1.000	1.000
Tryptophyl-Phenylalanine	147.8±102.81	260.82±265.28	212.34±244.28	0.969	1.000	1.000

Wilcoxon rank-sum test was used on the metabolites. *P* value was adjusted using Benjamin Hochberg false discovery rate (FDR).

Tab. S17 The effects of hydroxychloroquine intake on urinary hydroxychloroquine and Desethylchloroquine

Parameters	B	SE	Wald	df	*P* value	OR	95% CI (lower-upper)
hydroxychloroquine intake	98.462	14407.234	0.000	1.000	0.995	0.000	0.000－0.000
Urinary Desethylchloroquine	0.080	0.063	1.601	1.000	0.206	1.083	0.957－1.226
Constant	-4.982	1.684	8.755	1.000	0.003	0.007	
hydroxychloroquine intake	100.177	17264.841	0.000	1.000	0.995	0.000	0.000－0.000
Urinary hydroxychloroquine	0.022	0.012	3.367	1.000	0.066	1.022	0.999－1.047
Constant	-5.425	1.735	9.780	1.000	0.002	0.004	

Binary logistic regression model was used.
Abbreviations: B, coefficient value; SE, standard error; df, degrees of freedom; 95%CI, 95% confidence interval

[illegible]

[illegible]

Metabolites	Fold Change	log2(FC)
[illegible]	[illegible]	[illegible]

[illegible]

Tab. S20 Metabolites with VIP >1 (Controls vs LN)

Metabolites	VIP
(-)-Riboflavin	1.298
(5.alpha.)-Androst-2-en-17-one	1.553
11-beta-Hydroxyandrosterone-3-glucuronide	2.026
11-Methyl-7-oxatetracyclo[6.3.1.01,6.04,11]dodecane	2.100
11-Oxo-androsterone glucuronide	1.418
12-Hydroxy-13-O-D-glucuronoside-octadec-9Z-enoate	2.222
12-oxo-20-dihydroxy-leukotriene B4	1.146
16alpha-hydroxydehydroepiandrosterone 3-sulfate	2.602
1H-Indole-1-carboxamide, 6-chloro-2,3-dihydro-5-methyl-N-[6-[(2-methyl-3-pyridinyl)oxy]-3-pyridinyl]-	1.750
1'-Hydroxymidazolam .beta.-D-glucuronide	6.120
1-Methylene-5.alpha.-androstan-3.alpha.-ol-17-one glucuronide	1.398
2,3-Dinor-6-keto-prostaglandin F1 a	1.468
2,3-Dinorthromboxane B1	1.479
2,4(1H,3H)-Pyrimidinedione, 6-[4-(2-chlorophenyl)-1-piperazinyl]-3-cyclohexyl-	1.415
2,6-Dichlorobenzoic acid	1.605
2.alpha.-Hydroxymethyltestosterone	1.127
2-Hydroxy-2,6,6-trimethylcyclohexanone	1.125
2-Isopropylmalic acid	1.652
2-Malonylacetate	1.187
2-Mercapto benzimidazole	1.242
2-octenoylglycine	1.059
3-(3-oxo-1H-indol-2-ylidene)-1H-indol-2-one	1.098
3.beta.,5.beta.-Tetrahydrocortisone	1.477
3-Hydroxy-2-(4-methylbenzoyl)-4H-1-benzopyran-4-one	2.872
3-Methoxybenzenepropanoic acid	1.163
4,5-Dihydro-drospirenone-3-sulfate	2.416
4-Hydroxy-2,6-dimethylaniline	1.241
5-(3',5')-Dihydroxyphenyl-gamma-valerolactone	1.556
5.alpha.-Androstan-3.beta.-ol-17-one sulfate	1.865
5.beta.-Dihydrotestosterone	1.231
5-Oxo-1-propyl-2-pyrrolidineacetic acid	1.088
7,8-dihydroxy-2H-chromen-2-one	2.628
Acesulfame	2.092
Acetyl-DL-carnitine	1.520
Acylcarnitine 15:5	2.415
Acylcarnitine 21:5	2.840
Androstadienone	1.502
Androsterone glucuronide	1.677
Arg-Gly-Asp	1.228
Azelaic acid	1.794
Benzenesulfonic acid	3.908
Bynoschlamic acid	1.267
Caffeic acid 4-sulfate	1.580
Celastrol	2.018
Chenodeoxyglycocholic acid	1.090
Cinncassiol C	1.955
Cinncassiol E	1.261
cis-3-Hexenyl pyruvate	1.777
Clonazepine	2.088
Clonazepine glucuronide	2.031
Codeine	1.979
Corticosterone	1.279
Cortolone-3-glucuronide	2.015
Cyclamate	2.228
cyclic N-Acetylserotonin glucuronide	2.459
Cymorcin monoglucoside	1.111
Cys-Val-2-Nal-Met	1.511
Daidzein 4'-O-glucuronide	1.401
Diethyl sebacate	2.050
Dihydroferulic acid 4-O-glucuronide	1.012
Dimethyl sebacate	1.663
DL-2-Fluorophenylglycine	1.516
Epicoetanol	1.557
Exemestane	1.096
Gibberellin A44	1.216
Gibberellin A82	2.456
Glycitein 7-O-glucuronide	1.294
Hydroferulic acid	1.320
Hydroxyphenylacetylglycine	1.212
Icariside B8	1.605
Lidocaine	2.258
LysoPA 19:2; LysoPA 19:2	1.756
LysoPI 15:0; LysoPI 15:0	1.587
Marmesin A/BB	1.963
Mesaconic acid	1.702
Methyl 7-epi-12-hydroxyjasmonate glucoside	1.240
Methylsyringin	1.280
Metribuzin	1.040
m-Hydroxybenzoylecgonine	1.712
Monobutylphthalate	1.098
Mycophenolic acid O-acyl-glucuronide	1.408
N-(2-Furoyl)glycine	1.972
N-(3-Methyl-2-oxohexyl)alanine	1.499
Nonsofibrin, human	1.460
Nifenazone	1.062
N-Undecanoylglycine	1.054
O-Desmethylmycophenolic acid	1.222
Olopatadine	5.480
o-Tyramine	1.128
PC(16:0/18:1(9Z))	2.975
Perillic acid	1.092
PG 18:0; PG(2:0/16:0)	1.698
PG 18:0; PG(9:0/9:0)	1.721
Phenyllactic acid	1.255
Phloretin	2.814
Prednisolone	1.069
Prednisone	1.197
Propofol .beta.-D-glucuronide	6.522
Prostaglandin I2	1.181
Pyrogallol-1-O-sulphate	1.602
Pyrogallol-2-O-glucuronide	1.858
Pyrogallol-2-O-sulphate	1.221
Retinyl beta-glucuronide	1.978
S-Adenosyl-L-methionine	2.521
Sagitolide A	1.607
Shikonin	1.308
Stachydrine	1.450
Styrene	2.667
Subaphylline	1.455
Sulfamethazine	4.562
Tamoxifen	1.028
Taurocholate	2.697
Tetrahydroaldosterone-3-glucuronide	1.458
TG 36:6; TG(12:2/12:2/12:2)	2.788
TG 46:10; TG(12:2/15:2/19:5)	2.187
Thromboxane B2	1.908
Trigonelline	1.088
Tryptophyl-Lysine	1.642
Tryptophyl-Phenylalanine	2.119
Valproic acid .beta.-D-glucuronide	1.095
ss-2-(4-Isopropylphenyl)-2-methylpropanal	1.562

VIP was calculated using PLS-DA analysis.
Abbreviation: VIP, Variable Importance in Projection

Tab. S21 The area under the ROC curve (AUC) of urinary metabolite based on lupus nephritis classification

Metabolite	AUC
(-)-Riboflavin	0.893
12-Hydroxy-13-O-D-glucuronoside-octadec-9Z-enoate	0.944
12-oxo-20-dihydroxy-leukotriene B4	0.918
1-Methylene-5.alpha.-androstan-3.alpha.-ol-17-one glucuronide	0.989
2,3-Dinor-6-keto-prostaglandin F1 a	0.959
2,3-Dinorthromboxane B1	0.983
2.alpha.-Hydroxymethylethisterone	0.867
2-Hydroxy-2,6,6-trimethylcyclohexanone	0.973
3-(3-oxo-1H-indol-2-ylidene)-1H-indol-2-one	0.907
3-Hydroxy-2-(4-methylbenzoyl)-4H-1-benzopyran-4-one	0.995
3-Methoxybenzenepropanoic acid	0.863
4,5-Dihydro-drospirenone-3-sulfate	0.962
5-(3',5')-Dihydroxyphenyl-gamma-valerolactone	0.863
7,8-dihydroxy-2H-chromen-2-one	0.995
Arg-Gly-Asp	0.861
Azelaic acid	0.998
Caffeic acid 4-sulfate	0.931
Cinncassiol C	0.952
Cinncassiol E	0.887
cis-3-Hexenyl pyruvate	0.991
Cymorcin monoglucoside	0.871
Cys-Val-2-Nal-Met	0.987
Daidzein 4'-O-glucuronide	0.850
Dimethyl sebacate	0.947
Epirosmanol	0.889
Icariside B8	0.882
Mammea A/BB	0.942
m-Hydroxybenzoylecgonine	0.979
Monobutylphthalate	0.858
N-(2-Furoyl)glycine	0.965
N-(5-Methyl-3-oxohexyl)alanine	0.862
Necrofibrin, human	0.979
Nifenazone	0.899
O-Desmethylmycophenolic acid	0.886
Olopatadine	0.986
o-Tyrosine	0.873
PC(16:0/16:1(9Z))	0.852
Perillic acid	0.964
Prostaglandin I3	0.850
Pyrogallol-1-O-sulphate	0.904
Pyrogallol-2-O-glucuronide	0.977
Pyrogallol-2-O-sulphate	0.858
Retinyl beta-glucuronide	0.977
Sapidolide A	0.882
Shikonin	0.862
Stachydrine	0.886
Sulfamethazine	1.000
Tamoxifen	0.904
TG 46:10; TG(12:3/15:2/19:5)	0.854
Thromboxane B3	0.890
Trigonelline	0.903
Tryptophyl-Lysine	0.888
Tryptophyl-Phenylalanine	0.980

The metabolites displayed in the table are based on area under ROC curve (AUCROC). Only the metabolites with AUC $\geq$ 0.85 are displayed. The 95% confidence interval is calculated using 500 bootstrappings.

[illegible]

Tab. S13 Metabolites showing Fold change>2 or <0.5 ([illegible] vs [illegible])

Metabolites	Fold Change	log2(FC)
(-)-Riboflavin	0.103	-3.278
(1R)-Nepetalic acid	0.427	-1.227
(2-Aminothiazol-4-yl)acetic acid	2.207	1.141
(3.alpha.)-Androst-2-en-17-one	2.297	1.199
(E)-10-Oxo-8-decenoic acid	0.433	-1.208
(S)-(+)-Citramalic acid	0.497	-1.008
(S)-[illegible] acid	0.497	-1.008
1-(3-Aminopropyl)-[illegible]	0.421	-1.249
11.alpha.-Hydroxyprogesterone .beta.-D-glucuronide	2.141	1.098
11-Methyl-7-oxatetracyclo[[illegible]]dodecane	12.211	3.610
12-Hydroxy-13-O-D-glucuronoside-octadec-9Z-enoate	0.099	-3.334
16alpha-hydroxydehydroepiandrosterone 3-sulfate	3.773	1.916
17.beta.-Estradiol 3-.beta.-D-glucuronide	0.470	-1.089
18-Oxo-deoxycorticosterone	0.399	-1.326
1H-Indole-1-carboxamide, 5-chloro-2,3-dihydro-3-methyl-N-[6-[(2-methyl-3-pyridinyl)oxy]-3-pyridinyl]-	6.557	2.713
1H-indole-3-carboxylic acid	0.430	-1.218
1-Hydroxy-2-pentanone	0.333	-1.586
1'-Hydroxymidazolam .beta.-D-glucuronide	738.305	9.528
1-Methylene-3.alpha.-androstan-3.alpha.-ol-17-one glucuronide	0.331	-1.597
2,3,6-Trimethylbenzaldehyde	0.428	-1.225
2,3-Dinor-8-iso-prostaglandin F1 a	0.273	-1.873
2,4(1H,3H)-Pyrimidinedione, 5-[4-[(2-chlorophenyl)-1-piperazinyl]-2-oxyethoxy]-	3.794	1.924
2,4-Dichlorobenzoic acid	0.488	-1.035
2,4-Dihydroxyacetophenone 5-sulfate	0.398	-1.329
2.alpha.-Hydroxymethyltestosterone	0.247	-2.020
2-Hydroxy-3-methylbutyric acid	2.010	1.007
2-Indolinone	0.414	-1.273
2-Methylacetate	4.288	2.107
2-Mercapto benzimidazole	0.357	-1.486
2-Methylbenzoic acid	2.377	1.249
3-[(3-oxo-1H-indol-2-ylidene)]-1H-indol-2-one	0.373	-1.422
3,4,5,6-Tetrahydrohippuric acid	0.426	-1.231
3.beta.,5.beta.-Tetrahydroaldosterone	2.201	1.138
3-Hydroxy-2-(4-methylbenzoyl)-4H-1-benzopyran-4-one	0.058	-4.102
3-Methoxybenzenepropanoic acid	0.328	-1.614
3-Methoxycyclohexanecarboxylic acid	2.529	1.339
4-(Ethoxymethyl)phenol	2.490	1.320
4,5-Dihydro-drospirenone-3-sulfate	0.090	-3.471
4-Formylbenzonitrile	0.400	-1.320
4-Hydroxy-2,3-dimethylaniline	0.255	-1.970
4-hydroxy-3-(sulfooxy)benzoic acid	0.280	-1.836
4-Hydroxy-5-(dihydroxyphenyl)-valeric acid-O-sulphate	0.266	-1.907
5,7-Dimethoxy-4'-hydroxyflavanone	0.311	-1.686
5.alpha.-Androstan-3.beta.-ol-17-one sulfate	2.283	1.191
5.beta.-Dihydrotestosterone	2.261	1.178
5-Heptanoylglycine	0.399	-1.326
5-Oxo-1-propyl-2-pyrrolidineacetic acid	0.381	-1.392
5-Phenylvaleric acid	0.311	-1.684
6-beta-hydroxyestriol	2.918	1.545
7-Methylxanthine	2.017	1.012
9-Oxo-nonanoic acid	0.434	-1.205
Acesulfame	0.047	-4.413
Acetyl-DL-carnitine	6.929	2.797
Acylcarnitine 10:0	2.114	1.080
Acylcarnitine 13:2	4.262	2.092
Acylcarnitine 21:0	20.808	4.379
Acylcarnitine 8:0	0.489	-1.032
Adrenosterone	2.084	1.060
AMP	0.478	-1.066
Androsterone glucuronide	2.430	1.281
Arg-Gly-Asp	0.333	-1.585
[illegible] A	0.422	-1.245
Azelaic acid	0.180	-2.472
Benzenesulfonic acid	179.895	7.491
Betonicine	0.376	-1.411
Caffeic acid 3-sulfate	0.200	-2.322
Caffeoylcholine	0.468	-1.095
[illegible]	3.633	1.861
Cinncassiol C	0.165	-2.597
Clozapine	5.606	2.487
Clozapine glucuronide	4.403	2.061
Codeine	2.334	1.223
Cortisol	3.213	1.684
Cortolone-3-glucuronide	2.562	1.357
cyclic N-Acetylserotonin glucuronide	37.336	5.223
Cyclohexyl acetate	2.097	1.068
Cyanin monoglucoside	0.476	-1.070
Dihydroxy-1H-indole glucuronide I	0.277	-1.853
DL-2-Fluorophenylglycine	0.243	-2.041
DL-3-Hydroxylysine	3.221	1.688
DL-Pyroglutamic acid	2.424	1.278
[illegible]	0.289	-1.790
[illegible] D	2.191	1.132
Estradiol	2.312	1.209
Ethyl imidazole-4-carboxylate	0.499	-1.002
[illegible]	0.459	-1.121
Garcinone C	2.377	1.250
Gibberellin A24	4.804	2.264
Gibberellin A53	21.239	4.410
Hippuric acid	0.456	-1.132
Hydroferulic acid	0.358	-1.481
[illegible] D2	0.331	-1.594
Lidocaine	13.262	3.730
L-Tryptophanamide	2.458	1.297
Lysine	2.224	1.153
LysoPA 18:2; LysoPA 18:2	3.512	1.812
LysoPI 18:0; LysoPI 18:0	3.468	1.794
Mammea A/BB	0.130	-2.941
Mesaconic acid	0.307	-1.703
Methyl 7-epi-12-hydroxyjasmonate glucoside	0.451	-1.150
Methylsyringin	2.714	1.440
Metribuzin	3.775	1.917
Mono-2-ethylhexyl phthalate	4.157	2.056
Monobutylphthalate	0.490	-1.029
Mycophenolic acid O-acyl-glucuronide	0.003	-8.383
N-(2-Methyl-3-oxohexanoyl)alanine	0.220	-2.185
N1-Acetylspermidine	0.427	-1.227
N-Undecanoylglycine	0.458	-1.132
Perillic acid	0.376	-1.412
PC 18:0; PC(2:0/18:0)	2.810	1.494
PC 18:0; PC(18:0/0:0)	2.942	1.557
Phloretin	21.664	4.437
Prednisolone phosphate	0.413	-1.276
Prednisone	4.022	2.008
Propofol .beta.-D-glucuronide	307.339	8.267
Pyrogallol-1-O-sulphate	0.187	-2.418
Pyrogallol-2-O-sulphate	0.133	-2.910
Retinyl beta-glucuronide	0.190	-2.396
S-Adenosyl-L-methionine	14.702	3.878
Sorbitol	0.473	-1.079
Stachydrine	0.204	-2.293
Styrene	18.705	4.225
Sulfadimidine	4.343	2.119
Tamoxifen	0.332	-1.590
Taurocholate	35.061	5.131
Thromboxane B2	0.274	-1.869
Trigonelline	0.233	-2.101
Tryptophyl-Lysine	0.189	-2.403
Tyramine-O-sulfate	0.467	-1.099
Valproic acid .beta.-D-glucuronide	3.783	1.921
cis-2,3-Dihydro-2-methylfuran	2.794	1.482
cis-3-[4-(isopropyl)phenyl]-2-methylpropanal	5.621	2.491

For paired fold change analysis, the algorithm first counts the total number of pairs with fold changes that are consistently above/below the specified FC threshold.
Abbreviation: [illegible]

Tab. S24 Metabolites with VIP >1 (Controls vs nonLN)

Metabolite	VIP
(-)-Riboflavin	1.535
(1'R)-Nepetalic acid	1.079
(5.alpha.)-Androst-2-en-17-one	1.285
1-(3-Aminopropyl)-4-aminobutanal	1.121
11-Methyl-7-oxatetracyclo[6.3.1.01,6.04,11]dodecane	3.045
12-Hydroxy-13-O-D-glucuronoside-octadec-9Z-enoate	2.308
16alpha-hydroxydehydroepiandrosterone 3-sulfate	2.157
1H-Indole-1-carboxamide, 6-chloro-2,3-dihydro-5-methyl-N-[6-[(2-methyl-3-pyridinyl)oxy]-3-pyridinyl]-	1.878
1'-Hydroxymidazolam .beta.-D-glucuronide	6.875
1-Methylene-5.alpha.-androstan-3.alpha.-ol-17-one glucuronide	1.405
2,3-Dinor-6-keto-prostaglandin F1 a	1.537
2,4(1H,3H)-Pyrimidinedione, 6-[4-(3-chlorophenyl)-1-piperazinyl]-3-cyclohexyl-	1.584
2,4-Dichlorobenzoic acid	1.800
2,4-Dihydroxyacetophenone 5-sulfate	1.060
2.alpha.-Hydroxymethylethisterone	1.715
2-Indolinone	1.189
2-Maleylacetate	1.583
2-Mercapto benzimidazole	1.769
3-(3-oxo-1H-indol-2-ylidene)-1H-indol-2-one	1.207
3,4,5,6-Tetrahydrohippuric acid	1.299
3.beta.,5.beta.-Tetrahydrocortisone	1.002
3-Hydroxy-2-(4-methylbenzoyl)-4H-1-benzopyran-4-one	3.070
3-Methoxybenzenepropanoic acid	1.703
4,5-Dihydro-drospirenone-3-sulfate	2.381
4-Hydroxy-2,6-dimethylaniline	2.086
4-hydroxy-3-(sulfooxy)benzoic acid	1.845
4-Hydroxy-5-(dihydroxyphenyl)-valeric acid-O-sulphate	2.009
5,7-Dimethoxy-4'-hydroxyflavanone	1.514
5.alpha.-Androstan-3.beta.-ol-17-one sulfate	1.585
5.beta.-Dihydrotestosterone	1.195
5-Heptenoylglycine	1.239
5-Oxo-1-propyl-2-pyrrolidineacetic acid	1.907
5-Phenylvaleric acid	1.481
6-beta-hydrocortisol	1.124
Acesulfame	1.995
Acetyl-DL-carnitine	1.896
Acylcarnitine 18:5	2.199
Acylcarnitine 21:5	3.314
Androsterone glucuronide	1.364
Arg-Gly-Asp	1.344
Asticolorin A	1.281
Azelaic acid	1.707
Benzenesulfonic acid	4.300
Caffeic acid 4-sulfate	1.902
Caffeoylcholine	1.260
Celastrol	1.949
Cinncassiol C	1.978
Clozapine	2.386
Clozapine glucuronide	2.385
Codeine	1.371
Cortisol	1.090
Cortolone-3-glucuronide	1.379
cyclic N-Acetylserotonin glucuronide	3.664
Cymarcin monoglucoside	1.098
Dihydroxy-1H-indole glucuronide I	1.396
DL-2-Fluorophenylglycine	2.216
DL-5-Hydroxylysine	1.065
Epirosmanol	1.473
Garcinone D	1.062
Gibberellin A64	1.703
Gibberellin A92	3.116
Hippuric acid	1.100
Hydroferulic acid	1.477
Icariside B8	1.727
Lidocaine	3.833
L-Propionylcarnitine	1.241
LysoPA 19:2; LysoPA 19:2	1.656
LysoPI 15:0; LysoPI 15:0	1.224
Mammea A/BB	2.087
Mesoxalic acid	1.759
Methyl 7-epi-12-hydroxyjasmonate glucoside	1.455
Methylsyringin	1.254
Metribuzin	1.277
Mono-2-ethylhexyl phthalate	1.123
Monobutylphthalate	1.054
Mycophenolic acid O-acyl-glucuronide	1.999
N-(5-Methyl-3-oxohexyl)alanine	2.047
N-Undecanoylglycine	1.254
Perillic acid	1.112
PG 18:0; PG(2:0/16:0)	1.521
PG 18:0; PG(9:0/9:0)	1.091
Phloretin	2.989
Prednisolone phosphate	1.140
Prednisone	1.658
Propofol .beta.-D-glucuronide	7.281
Pyrogallol-1-O-sulphate	2.196
Pyrogallol-2-O-sulphate	1.476
Retinyl beta-glucuronide	1.376
S-Adenosyl-L-methionine	2.819
Stachydrine	1.876
Styrene	3.024
Subaphylline	1.666
Tamoxifen	1.034
Taurocholate	3.121
Thromboxane B3	1.927
Trigonelline	1.706
Tryptophyl-Lysine	1.970
Tyramine-O-sulfate	1.740
Valproic acid .beta.-D-glucuronide	1.270
xi-2,3-Dihydro-3-methylfuran	1.247
xi-3-(4-Isopropylphenyl)-2-methylpropanal	1.336

VIP was calculated using PLS-DA analysis.
Abbreviation: LN, lupus nephritis; VIP, Variable Importance in Projection

Tab. S25 The area under the ROC curve (AUC) of urinary metabolite based on non-lupus nephritis classification

Metabolite	AUC
12-Hydroxy-13-O-D-glucuronoside-octadec-9Z-enoate	0.945
1-Methylene-5.alpha.-androstan-3.alpha.-ol-17-one glucuronide	1.000
2,3-Dinor-6-keto-prostaglandin F1 a	0.978
2,4-Dihydroxyacetophenone 5-sulfate	0.856
2.alpha.-Hydroxymethylethisterone	0.925
2-Mercapto benzimidazole	0.851
3-(3-oxo-1H-indol-2-ylidene)-1H-indol-2-one	0.925
3-Hydroxy-2-(4-methylbenzoyl)-4H-1-benzopyran-4-one	0.995
3-Methoxybenzenepropanoic acid	0.969
4,5-Dihydro-drospirenone-3-sulfate	0.985
4-Hydroxy-2,6-dimethylaniline	0.887
4-hydroxy-3-(sulfooxy)benzoic acid	0.878
5,7-Dimethoxy-4'-hydroxyflavanone	0.860
5-Phenylvaleric acid	0.971
Androsterone glucuronide	0.853
Azelaic acid	0.998
Caffeic acid 4-sulfate	0.893
Caffeoylcholine	0.891
DL-2-Fluorophenylglycine	0.918
Hippuric acid	0.875
Hydroferulic acid	0.856
Icariside B8	0.880
N-(5-Methyl-3-oxohexyl)alanine	0.947
Perillic acid	0.904
Phloretin	0.931
Pyrogallol-1-O-sulphate	0.938
Pyrogallol-2-O-sulphate	0.976
Retinyl beta-glucuronide	0.869
S-Adenosyl-L-methionine	0.971
Stachydrine	0.951
Tamoxifen	0.891
Thromboxane B3	0.876
Trigonelline	0.993
Tryptophyl-Lysine	0.927

The metabolites displayed in the table are based on area under ROC curve (AUCROC). Only the metabolites with AUC ≥ 0.85 are displayed. The 95% confidence interval is calculated using 500 bootstrappings.

[illegible]

Tab. S27 Correlation between bacterial genus and cytokines that showed significant difference between controls and SLE

Genus	Cytokine	r value	*P* value	relation
Bacteroides	MIP-1b	0.322	0.001	positive
Bacteroides	IP-10	0.330	0.001	positive
Bacteroides	IL-12	-0.339	0.001	negtive
Bacteroides	IL-17	0.377	0.000	positive
Bacteroides	Eotaxin	0.426	0.000	positive
Gardnerella	IL-5	0.332	0.001	positive
Gardnerella	IL-12	0.348	0.000	positive
Haemophilus	IL-13	0.317	0.002	positive
Haemophilus	IL-5	0.364	0.000	positive
Haemophilus	IL-12	0.367	0.000	positive
Megamonas	IP-10	0.368	0.000	positive
Phocaeicola	Eotaxin	0.304	0.002	positive
Phocaeicola	IL-2	-0.318	0.002	negtive
Phocaeicola	IL-8	0.327	0.001	positive
Phocaeicola	IL-13	-0.423	0.000	negtive
Phocaeicola	IL-12	-0.431	0.000	negtive
Pseudomonas	MIP-1b	-0.306	0.002	negtive
Pseudomonas	G-CSF	-0.321	0.001	negtive
Pseudomonas	IP-10	-0.389	0.000	negtive
Pseudomonas	IL-8	-0.512	0.000	negtive
Rothia	IL-2	0.360	0.000	positive
Rothia	IL-13	0.393	0.000	positive
Rothia	IL-5	0.449	0.000	positive
Rothia	IL-12	0.459	0.000	positive
Sphingomonas	IL-12	0.317	0.002	positive
Sphingomonas	IL-8	-0.356	0.000	negtive
Staphylococcus	IL-2	0.315	0.002	positive
Staphylococcus	IL-8	-0.323	0.001	negtive
Staphylococcus	IL-12	0.375	0.000	positive
Staphylococcus	IP-10	-0.387	0.000	negtive
Staphylococcus	IL-13	0.443	0.000	positive
Staphylococcus	IL-5	0.457	0.000	positive
Streptococcus	Eotaxin	-0.333	0.001	negtive
Streptococcus	IL-13	0.358	0.000	positive
Streptococcus	IP-10	-0.368	0.000	negtive

Spearman correlation evaluated the linear relationship between bacterial genus and cytokine.

Tab. S28 Correlation between bacterial genus and disease profiles in SLE patients

Genus	disease profile	*r* value	*P* value	relation
Bacteroides	Uric acid	0.428	0.002	positive
Haemophilus	Uric acid	0.364	0.010	positive
Megamonas	C3	-0.361	0.011	negtive
Streptococcus	IgG	0.349	0.014	positive
Phocaeicola	C3	-0.310	0.030	negtive

Spearman correlation evaluated the linear relationship between bacterial genus and disease profile.
